# Exploring neurocognitive factors and brain activation in adult cochlear implant recipients associated with speech perception outcomes—A scoping review

**DOI:** 10.3389/fnins.2023.1046669

**Published:** 2023-02-02

**Authors:** Loes Beckers, Nikki Tromp, Birgit Philips, Emmanuel Mylanus, Wendy Huinck

**Affiliations:** ^1^Cochlear Ltd., Mechelen, Belgium; ^2^Department of Otorhinolaryngology, Donders Institute for Brain, Cognition and Behaviour, Radboud University Medical Center, Nijmegen, Netherlands

**Keywords:** cochlear implants, neurocognition, scoping review, sensorineural hearing loss, postlingual, speech perception

## Abstract

**Background:**

Cochlear implants (CIs) are considered an effective treatment for severe-to-profound sensorineural hearing loss. However, speech perception outcomes are highly variable among adult CI recipients. Top-down neurocognitive factors have been hypothesized to contribute to this variation that is currently only partly explained by biological and audiological factors. Studies investigating this, use varying methods and observe varying outcomes, and their relevance has yet to be evaluated in a review. Gathering and structuring this evidence in this scoping review provides a clear overview of where this research line currently stands, with the aim of guiding future research.

**Objective:**

To understand to which extent different neurocognitive factors influence speech perception in adult CI users with a postlingual onset of hearing loss, by systematically reviewing the literature.

**Methods:**

A systematic scoping review was performed according to the PRISMA guidelines. Studies investigating the influence of one or more neurocognitive factors on speech perception post-implantation were included. Word and sentence perception in quiet and noise were included as speech perception outcome metrics and six key neurocognitive domains, as defined by the DSM-5, were covered during the literature search (Protocol in open science registries: 10.17605/OSF.IO/Z3G7W of searches in June 2020, April 2022).

**Results:**

From 5,668 retrieved articles, 54 articles were included and grouped into three categories using different measures to relate to speech perception outcomes: (1) Nineteen studies investigating brain activation, (2) Thirty-one investigating performance on cognitive tests, and (3) Eighteen investigating linguistic skills.

**Conclusion:**

The use of cognitive functions, recruiting the frontal cortex, the use of visual cues, recruiting the occipital cortex, and the temporal cortex still available for language processing, are beneficial for adult CI users. Cognitive assessments indicate that performance on non-verbal intelligence tasks positively correlated with speech perception outcomes. Performance on auditory or visual working memory, learning, memory and vocabulary tasks were unrelated to speech perception outcomes and performance on the Stroop task not to word perception in quiet. However, there are still many uncertainties regarding the explanation of inconsistent results between papers and more comprehensive studies are needed e.g., including different assessment times, or combining neuroimaging and behavioral measures.

**Systematic review registration:**

https://doi.org/10.17605/OSF.IO/Z3G7W.

## 1. Introduction

Cochlear implants (CIs) are considered an effective treatment for severe-to-profound sensorineural hearing loss, when hearing aids provide insufficient benefits. However, speech perception performance outcomes of this treatment are highly variable among adult CI listeners (Holden et al., 2013). Different biological and audiological factors, such as residual hearing before implantation and duration of hearing loss, only contribute to a small extent when explaining this variation (Zhao et al., 2020). A multicentre study using data from 2,735 adult CI users investigated how much variance in word perception outcomes in quiet could be explained by previously identified factors. When including 17 predictive factors (e.g., duration of hearing loss, etiology, being a native speaker, age at implantation, and preoperative hearing performance) in a linear regression model, the variance explained was only 0.12–0.21 (Goudey et al., 2021).

To decrease uncertainty, other factors, such as (neuro)cognition need to be considered. Neurocognitive factors are skills used to acquire knowledge and manipulate information and reasoning. In addition to bottom-up factors, top-down neurocognitive factors have been proposed to contribute to variation in postoperative speech perception (Baskent et al., 2016; Moberly et al., 2016a). In this context, top-down processing means that higher-order cognitive processes drive lower-order systems. For example, prior knowledge is used for processing incoming information from the senses such as speech (bottom-up information). Bottom-up processes are lower-order mechanisms that, in turn, can trigger additional higher-order processing (Breedlove and Watson, 2013). Interactions of top-down processes and neurocognitive functions with the incoming speech signal, have been shown to be highly important for distorted speech recognition (Davis and Johnsrude, 2007; Stenfelt and Rönnberg, 2009; Mattys et al., 2012). Given that speech signal output from a CI is distorted, neurocognitive mechanisms are needed for active and effortful decoding of this speech. This is thought to enable CI listeners to compensate for the loss of spectro-temporal resolution (Baskent et al., 2016; Moberly et al., 2016a). Several studies have investigated the association of neurocognitive factors and brain activation patterns with CI performance. These studies did not only use varying designs and methods, but also observed varying results. A literature review may help interpret and summarize these outcomes. After a preliminary search for existing reviews in PROSPERO and PubMed (June 2020) showed that these studies were not collected and evaluated in a review before, this scoping review was initiated.

**FIGURE 1:**
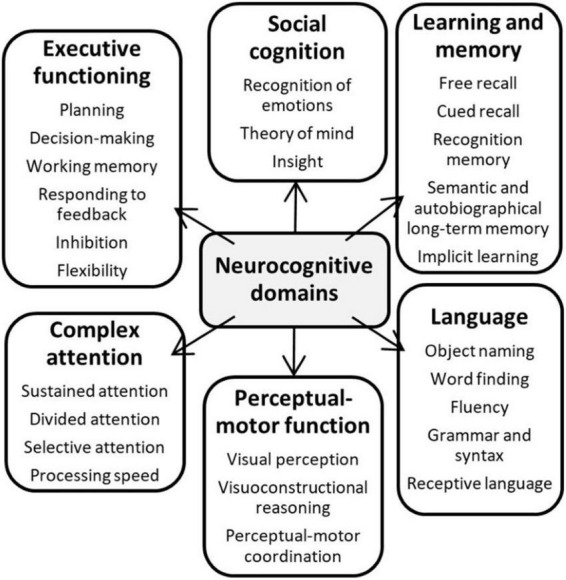
Key cognitive domains defined by the Diagnostic Statistical Manual of Mental Disorders, Fifth Edition (DSM-5). [Source: Sachdev et al. (2014)].

The objective of this scoping review is to gain understanding of which brain activation patterns and top-down neurocognitive factors are associated with speech perception outcomes in hearing-impaired adults after cochlear implantation. This is also done by exploring differences between poorer and better performers. When referring to top-down neurocognitive factors or mechanisms, we refer to the ones that can be classified under one of six neurocognitive domains, defined in the Diagnostic Statistical Manual of Mental Disorders, Fifth Edition (DSM-5); (1) complex attention, (2) executive function, (3) social cognition, (4) learning and memory, (5) perceptual-motor function, and (6) language (Figure 1; Sachdev et al., 2014).

1. **Complex attention** involves sustained attention, divided attention, selective attention, and processing speed. Attention is a state or condition of selective awareness or perceptual receptivity by which a single stimulus or task (sustained), or several (divided) are selected for enhanced processing, while possibly other irrelevant stimuli, thoughts, and actions are ignored (selective). Cortical regions that play an important role in attentional processes are the posterior parietal lobe and cingulate cortex (Breedlove and Watson, 2013).
2. **Executive function** includes planning, decision-making, working memory, responding to feedback, inhibition, flexibility, and non-verbal intelligence – all high-level control processes that manage other cognitive functions important for generating meaningful goal-oriented behavior. The frontal lobe is mainly involved in these processes (Breedlove and Watson, 2013).
3. **Social cognition** refers to cognitive processes involved in social behavior (Hogg and Vaughan, 2018). In other words, how people think about themselves and others and how these processes affect judgment and behavior in a social context, leading to socially appropriate or less appropriate behavior. These behaviors include the recognition of emotions, having theory of mind and insight (Sachdev et al., 2014).
4. **Learning and memory** include short-term memory, measured by free and cued recall, recognition memory, semantic and autobiographical long-term memory, and implicit learning. Learning is acquiring new and relatively enduring information, behavior patterns or abilities, because of practice or experience. Memory is the ability to store learned information and retrieve or reactivate it over time. Structures of the limbic system, the temporal and frontal cortex are mainly involved in memory formation, but plasticity within the brain also indicates learning (Breedlove and Watson, 2013).
5. **Perceptual-motor function** includes visual perception, visuoconstructional reasoning and perceptual-motor coordination (Sachdev et al., 2014). These are processes involved in movement and being able to interact with the environment.
6. **Language**, the most sophisticated structured system for communicating (Breedlove and Watson, 2013), encompasses skills needed for both language production (object naming, word finding, fluency, grammar and syntax) and language comprehension (receptive language and grammar and syntax). Areas involved in language processing are Broca’s area in the frontal lobe, along with the primary motor cortex, the supramarginal gyrus in the parietal cortex, and Wernicke’s area, primary auditory cortex and angular gyrus in the temporal cortex (Breedlove and Watson, 2013).

These domains are not mutually exclusive, meaning that some cognitive functions might be part of processes underlying other cognitive functions. For example, social cognitive skills involve executive functions, such as decision-making. In the same way, this review will explore which cognitive factors are involved in or part of speech perception processing in adult CI users, which can be classified as a neurocognitive factor under the language domain. Furthermore, CI users might recruit several alternative brain regions during auditory and speech perception. Identifying these activation patterns could pinpoint neurocognitive mechanisms that facilitate or constrain speech perception outcomes (Lazard et al., 2010). Therefore, in addition to studies including behavioral cognitive measures, studies using neuroimaging metrics will be explored.

In this review, speech perception outcomes encompass word or sentence perception in quiet and noise. Besides assessing CI performance, some studies use these speech perception outcome metrics to classify patients as good or poor performers (e.g., Suh et al., 2015; Kessler et al., 2020; Völter et al., 2021). However, there are no general guidelines for classifying good and poor performers, resulting in varying performance classification between studies. See for example, Kessler et al. (2020), divided good and poor performers based on sentence perception in noise. Other examples with respect to word perception in quiet are Völter et al. (2017), who used as cut-off scores >30 and <70% for, respectively poor and good performers, while Mortensen et al. (2006), opted for >60 and <96% limits. Suh et al. (2015) used 80% speech perception score to split between poor and good performers. Therefore, when discussing studies having implemented performance classification, their participants will be referred to as “better” and “poorer” performers in this review.

Discussing and summarizing the wide variety of studies investigating the association between neurocognitive factors and CI performance in a systematic scoping review might provide new insights and guide new research on this topic. Research in this field helps understand CI outcome variation and could be particularly valuable to improve care for poorer performing adult CI listeners. Being able to more accurately predict performance outcomes will facilitate managing their expectations. Furthermore, identification of the root causes of poorer performance could lead to the development of individualized aftercare.

## 2. Methods

### 2.1. Protocol and registration

The Preferred Reporting Items for Systematic Reviews and Meta-Analysis (PRISMA) was used for this systematic scoping review (Moher et al., 2009). A systematic scoping review was performed instead of a systematic literature review because of the variability in methods between the included studies. Therefore, this review does not include any meta-analysis or risk of bias assessment. Furthermore, Population, Concept, and Context (PCC) was used as the research question framework (Peters et al., 2015). The population being postlingually deaf adult CI users, the concept being speech perception outcomes, in the context of neurocognition. The protocol of this review was registered in the open science registries 10.17605/OSF.IO/Z3G7W.

### 2.2. Eligibility criteria

**FIGURE 2:**
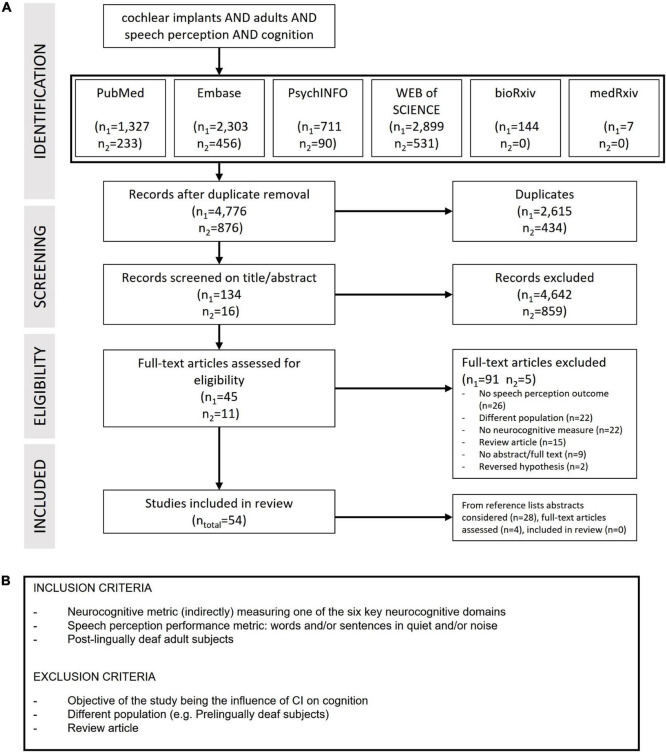
**(A)** Preferred reporting items for systematic reviews and meta-analysis (PRISMA) flowchart of the literature search and study selection. Last date of first search June 2020, numbers are indicated with n_1_. Last date of second search April 2022, numbers are indicated with n_2_. **(B)** Inclusion and exclusion criteria of the articles.

This review encompasses studies investigating the influence of one or more neurocognitive factors on speech perception after cochlear implantation. Word and sentence perception in quiet and noise were included as speech perception outcome metrics. Studies including participants listening in both unimodal (CI-only), bimodal (CI and hearing aid) and bilateral (CI both ears) conditions were eligible. To provide a complete overview, the six key neurocognitive domains as defined by the DSM-5 were covered during the literature search. No limitations on cognitive measures were implemented. Included study designs were cross-sectional studies, non-randomized control trials, quasi-experimental studies, longitudinal studies, prospective and retrospective cohort studies and meta-analysis performed in a clinical setting. Studies from publication year 2000 and onward were included. Furthermore, all studies involving children and adults with a prelingual onset of deafness were excluded. There were no restrictions on publication status or language of publication (Figure 2B).

### 2.3. Data sources and search strategy

Four scientific databases: PubMed, Embase, PsychInfo and Web of Science were searched. BioRxiv and medRxiv were used to search for any preprints. Terms and their synonyms related to the outcomes, predictive factors based on the DSM-5 neurocognitive domains and patient population were included in the search strategy. Thesauruses like MeSH and Emtree were used besides free-text terms in titles and abstracts. The search strategies for each database can be found in Supplementary Material Part A. Reference lists of articles were scanned for additional suitable studies. Systematic searches were conducted up to July 2020 and assisted by a trained librarian. In April 2022 a second search was performed using the same protocol.

### 2.4. Study selection

Literature screening was performed in two steps. First, the results of all databases were merged. Duplicates were removed using Rayyan QCRI systematics review app (Ouzzani et al., 2016) and Endnote (EndNote X9, 2013, Clarivate, Philadelphia, PA, USA). Second, two authors (LB and NT) blindly selected relevant studies by screening titles and abstracts based on the eligibility criteria in the same app. In case it was unclear from the title and abstract if an article should be included, the decision was made based on full-text. Any study selection conflict was resolved by discussion between two authors (LB and NT).

### 2.5. Data extraction and management

After screening all included publications, a custom data extraction form was used for data capturing, which was piloted before data collection commencement. The final form included details relating to study design, participants, eligibility criteria, hearing device, speech perception measurement, cognitive measurement, relation between cognitive measurement and outcome, analysis method, limitations, possible biases and the conclusion of the author.

## 3. Results

A total of 5,652 unique articles were retrieved. After screening titles, abstracts of 150 articles remained for full-text screening. Of these 150 articles, 96 were excluded based on reading the full-text. In 26 studies, there was no speech perception outcome reported or used in the relevant analysis (Giraud et al., 2000, 2001a,b; Gfeller et al., 2003; Oba et al., 2013; Berding et al., 2015; Finke et al., 2015; Jorgensen and Messersmith, 2015; Song et al., 2015b; Wang et al., 2015; McKay et al., 2016; Shafiro et al., 2016; Perreau et al., 2017; Amichetti et al., 2018; Butera et al., 2018; Bönitz et al., 2018; Cartocci et al., 2018; Lawrence et al., 2018; Moberly et al., 2018b; Patro and Mendel, 2018, 2020; Dimitrijevic et al., 2019; Chari et al., 2020; Zaltz et al., 2020; Schierholz et al., 2021; Abdel-Latif and Meister, 2022). Twenty-two studies were excluded based on population criteria, studies testing children and adults with prelingual onset of hearing loss (El-Kashlan et al., 2001; Most and Adi-Bensaid, 2001; Lyxell et al., 2003; Rönnberg, 2003; Middlebrooks et al., 2005; Doucet et al., 2006; Heydebrand et al., 2007; Rouger et al., 2007; Hafter, 2010; Li et al., 2013; Lazard et al., 2014; Bisconti et al., 2016; Anderson et al., 2017; Finke et al., 2017; Miller et al., 2017; Moradi et al., 2017; Purdy et al., 2017; McKee et al., 2018; Verhulst et al., 2018; Winn and Moore, 2018; Lee et al., 2019; Smith et al., 2019). In 22 studies, no neurocognitive measure was present (Meyer et al., 2000; Vitevitch et al., 2000; Wable et al., 2000; Giraud and Truy, 2002; Lachs et al., 2002; Lonka et al., 2004; Kelly et al., 2005; Debener et al., 2008; Tremblay et al., 2010; Winn et al., 2013; Moberly et al., 2014; Turgeon et al., 2014; Ramos-Miguel et al., 2015; Collett et al., 2016; Purdy and Kelly, 2016; Sterling Wilkinson Sheffield et al., 2016; Harris et al., 2017; Alemi and Lehmann, 2019; Balkenhol et al., 2020; Crowson et al., 2020; Naples and Berryhill McCarty, 2020; Lee et al., 2021). Fifteen reviews were excluded as they did not include an original study (Wilson et al., 2003, 2011; Mitchell and Maslin, 2007; Peterson et al., 2010; Aggarwal and Green, 2012; Anderson and Kraus, 2013; Lazard et al., 2013; Anderson and Jenkins, 2015; Baskent et al., 2016; Pisoni et al., 2016, 2017; Wallace, 2017; Oxenham, 2018; Bortfeld, 2019; Glennon et al., 2020). Two articles were excluded because they focused on a reversed hypothesis (the influence of CI on cognition) (Anderson and Jenkins, 2015; Nagels et al., 2019) and nine articles were excluded because no abstract and/or full-text paper was available. Fifty-four articles remained after full-text screening. From scanning the references lists of these papers, 28 abstracts were considered. After reading four full-text papers (Lee et al., 2001; Suh et al., 2009; Tao et al., 2014; Wagner et al., 2017), none of the articles were included, leading to 54 included articles (Figure 2A).

The selected articles were grouped into three categories: (1) Studies investigating brain activation patterns in CI users in relation to speech perception performance (*N* = 18), this includes articles assessing cross-modal activation, (2) Studies investigating performance on cognitive tests in relation to performance on speech perception tests (*N* = 17), and (3) Studies investigating the use of linguistic skills and information and the relationship with speech perception performance (*N* = 5). Note that some studies investigated both brain activation and cognitive and linguistic functions (*N* = 1), or cognitive and linguistic skills (*N* = 13). Each category of studies will be discussed below. An overview of these studies is shown in Supplementary Tables 1–4.

### 3.1. Brain activation

**FIGURE 3:**
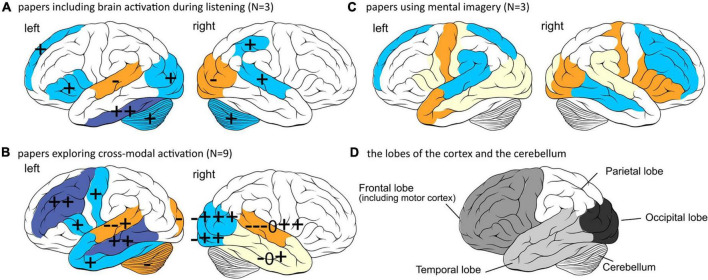
Regions of the cortex found to be activated in the papers related to speech perception outcomes. +, –, and 0 indicates a positive correlation, negative correlation, or null results, respectively found in an included paper. Accuracy of the depiction depends on accuracy of the reports, neuroimaging and analysis technique used in the papers. Top: left hemisphere, Bottom: right hemisphere. **(A)** The parts of the cortex found to be activated during auditory perception related to speech perception outcomes. Blue areas are found to be positively correlated and orange areas negative. **(B)** The parts of the cortex found to be activated during auditory perception, and visual perception. Blue areas are found to be mostly positively correlated and orange areas mostly negative. Yellow areas show conflicting results. The right amygdala (+), cingulate sulcus (+) and bilateral thalami (–) are not depicted because they are not located on the outside cortex. **(C)** The parts of the cortex found to be activated during speech imagery tasks preoperatively. Blue areas are found to be positively correlated and orange areas negative. Yellow areas show conflicting results. Since most of this data is from the same participant group, no signs are used to indicate findings per paper. Left and right medial temporal lobes including hippocampal gyrus are not depicted because they are not located on the outside cortex. **(D)** The lobes of the cortex (frontal, parietal, temporal, and occipital lobe) and the cerebellum. This image can be used as a guidance to read the text and interpret part **(A–C)** of the figure. The outline of the brain was drawn by Patrick J. Lynch, medical illustrator and C. Carl Jaffe, MD, cardiologist, https://creativecommons.org/licenses/by/2.5/.

Three of the 15 studies observed activation patterns during auditory or speech perception, whereas nine focused on cross-modal activation. Three papers used speech imagery tasks preoperatively instead of speech perception tasks. These studies are discussed below. To better understand the brain areas involved, data are visualized in Figure 3.

#### 3.1.1. Brain responses to auditory stimuli

Three studies divided their participants into better and poorer performers based on speech perception performance and explored the differences in brain activation while listening. These are Mortensen et al. (2006); Suh et al. (2015), Kessler et al. (2020) (see Table 1 for an overview) and are summarized below:

**TABLE 1:** Overview of included papers studying brain responses to auditory stimuli.

References, sample size	Method	Speech perception measure	Statistical test (y/n) indicating a power analysis	Key findings
Mortensen et al., 2006, Nbetter = 7 Npoorer = 5	PET during several speech and non-speech stimuli	SQ	*T*-test of high performing vs low performing group (n)	(+) The better performers showed more activation in the left inferior prefrontal and right anterior and posterior temporal cortex and the right cerebellum. (–) The poorer performers showed more activation in the left temporal areas *p* < 0.05.
Suh et al., 2015, *N* = 15	PET during noise–preoperatively	WQ + SQ	Mann-Whitney U test for difference in means (n)	(+) ITG and premotor area in better performers *p* = 0.0005. (–) Occipital area in poorer performers *p* = 0.01.
Kessler et al., 2020, *N* = 21 (see also Tables 4, 8, 10)	SPECT scan and EEG during semantic correct vs. incorrect sentences	WQ SQ + N	Independent *T*-test and difference images (n)	Sentence test groups: (+) Better performers show higher activation in the left occipital area and right temporal area (*p* < 0.001) during task. (–) Poorer performers show higher activation in the left and right frontal BA9 and left ITG (*p* < 0.001) during task.

For each paper sample size (N), neuroimaging method, speech perception outcome measure (WQ, words in quiet; SQ, sentences in quiet; SQ+N, sentences in quiet and noise); statistical test [including a report of a power analysis (y), yes; (n), no], and key findings are reported [(+), positive significant result; (–) negative significant result; *(ns)* *non-significant result*]. EEG, electroencephalography, ITG, inferior temporal gyrus, PET, positron emission tomography, SPECT, single-photon emission computed tomography. A more detailed version can be found in Supplementary Table 2.

First, Mortensen and colleagues showed alternative patterns of activation between better performers (96–100% word score in quiet) and poorer performers (<60% word score in quiet), while listening passively to a range of speech and non-speech stimuli. Better performers showed increased activity in the left inferior prefrontal, left and right anterior and posterior temporal cortex (auditory cortex), and the right cerebellum. Poorer performers only showed increased activity in the left temporal areas (*p* < 0.05) (Mortensen et al., 2006).

Second, Suh et al. (2015) measured preoperative brain activation during listening to noise and compared the results of a group of postoperative poorer and better (cutoff: 80%-word score) performers. Participants with higher activity in the inferior temporal gyrus and premotor areas (part of frontal cortex) became better performers (*p* = 0.005), and participants with higher activation in the occipital lobe (visual cortex) became poorer performers (*p* = 0.01) (Suh et al., 2015).

In the third study, Kessler et al. (2020), examined brain activation during a speech discrimination task consisting of correct and incorrect sentences. When dividing the group of participants into better and poorer performers [median split with cutoff +7.6 dB Signal to Noise Ratio (SNR) on a sentence test in noise], better performers showed significantly higher activity in the right parietal and temporal area and left occipital area (*p* < 0.001, uncorrected for multiple comparisons), and poorer performers significantly higher activation in the superior frontal areas (*p* < 0.001, uncorrected for multiple comparisons). Activity during resting state revealed that poorer performers had a higher activity in the right motor and premotor cortex and right parietal cortex, whereas better performers had higher activity in the left hippocampal area, left inferior frontal areas and left inferior temporal cortex (*p* < 0.001, uncorrected for multiple comparisons). The differences in activity between better and poorer performers in the bilateral temporal, frontal, parietal, and bilateral motor cortex were significantly positively correlated with performance on a monosyllabic word test and the MWT-B verbal intelligence test (*p* > 0.001, uncorrected and *p* < 0.05 FWE). There were also small positive correlations between this activity in the left temporal, parietal and occipital regions with working memory span scores, and activity in the left temporal lobe with a verbal learning task (only in testing without correction for multiple comparisons and not in tests including FWE) (Kessler et al., 2020).

#### 3.1.2. Responses to audio, visual and audio-visual stimuli indicating cross-modal activation

In individuals with hearing loss cross-modal activation occurs when two things are at play. (1) The visual cortex is involved in auditory perception. (2) The auditory cortex is also recruited and used to process visual stimuli instead of or in addition to auditory stimuli to understand speech (Bavelier and Neville, 2002). Several studies have investigated whether such reorganization occurs in postlingually deaf participants and whether it is related to postoperative speech perception performance, as this reorganization might limit these areas to return to their original functioning (see Table 2 for an overview). These studies are summarized below (* indicates whether the study reported sufficient power):

**TABLE 2:** Overview of included papers studying cross-modal activation.

References, sample size	Method	Speech perception measure	Statistical test, (y/n) indicating a power analysis reported	Key findings
Buckley and Tobey, 2010, *N* = 12	EEG (N1, Visual evoked potential) during presentation of visual gradients	WQ SN	Linear regression analysis of word and sentence scores against the amplitude of the N1 response. ROI: temporal lobe (y).	*(ns) r* = *0.1618, p* = *0.6155*
Sandmann et al., 2012, *N* = 11	EEG (P100, N150, P270) during presentation of visual checkerboard patters	WQ SN	Spearmans rank correlations between ERPs and speech perception (n).	WQ: (–) Right auditory cortex level 3 *r* = –0.78, *p* < 0.05, level 4: *r* = –0.75, *p* < 0.05 for right implanted participants SN: (+) Right auditory cortex level 3: (*ns) r* = *0.63, p* = *0.07*, level 4: *r* = 0.72, *p* < 0.05
Strelnikov et al., 2013, *N* = 10	PET during auditory and visual words vs. non-word presentation	WQ	Regression analysis and correlation analysis with family-wise error correction *p* < 0.05 (n).	(+) The right occipital cortex during rest: *r* = 0.9, during visual stimuli: *r* = 0.8 and audiovisual stimuli: *r* = 0.5, *p* < 0.05, In the left inferior frontal pole during rest *r* = 0.809, visual stimuli: *r* = 0.77 and audiovisual stimuli: *r* = 0.90 *p* < 0.05 (–) In the middle STG/STS and occipital cortex during rest: *r* = –0.9, visual stimuli: *r* = –0.8 and audiovisual stimuli: *r* = –0.7, *p* < 0.05
Song et al., 2015a, *N* = 10	PET during video with a speaker saying digits in auditory, visual and audiovisual condition congruent and incongruent – preoperatively	WQ	Correlation analysis between contrast images of each condition and word perception scores. Controlled for sex and age. P = 0.001 threshold (n).	(+) During congruent audiovisual stimuli the amygdala rho = 0.888, *p* = 0.008 (–) During congruent audiovisual stimuli the left rho = –0.826, *p* = 0.013 and right rho = –0.777, *p* = 0.019 occipital gyrus
Kim et al., 2016, *N* = 14	EEG (VEP) while patterned visual stimuli are presented	WQ	Spearman correlation analysis between words scores and amplitude and latency of P1 in ROIs: occipital and temporal electrodes, (y, but sample size insufficient).	(+) Larger P1 amplitude in occipital cortex *r* = 0.755, *p* = 0.001 Central visual field size *r* = 0.699, *p* = 0.009 (–) Larger P1 in right temporal cortex *r* = –0.736, *p* = 0.003
			Mann-whitney test to compare means per group.	(+) P1 in occipital cortex larger *p* = 0.013 in better performers (–) P1 in right temporal cortex smaller in better performers *p* = 0.002
Chen et al., 2016, *N* = 19	fNIRS during visual checkerboard stimuli and auditory stimuli	SQ + N	Pearsons correlation analysis between activation differences condition and SQ + N. ROI: right occipital cortex and left, right temporal cortex (n).	(+) *r* = 0.518, *p* = 0.027
Chen et al., 2017, *N* = 19	fNIRS during visual checkerboard stimuli and auditory word and reversed words	WQ SQ + N	Spearman correlation analysis between cross modal activation and speech recognition. ROI: temporal and occipital cortex (n).	WQ: (+) More cross modal plasticity for auditory than for visual stimuli *r* = 0.525, *p* = 0.021 SQ + N: (*ns)*
Zhou et al., 2018, *N* = 15	fNIRS during audio, visual and audiovisual speechreading	WQ, SQ + N	Pearson correlation between activation levels and speech test scores. ROI: STG/STS (n).	(–) Left STS and STG *r* = –0.668, *p* = 0.009
Han et al., 2019, *N* = 27	PET during noise, no instruction – preoperatively	WQ	Pearson correlation between change in brain metabolism (*p* = 0.001) and speech test scores (n).	(+) Dorsolateral and dorsomedial frontal areas *r* = 0.595, *p* > 0.001 (–) Superior occipital gyrus *r* = –0.538, *p* < 0.001
Sun et al., 2021, *N* = 94	MRI scan looking at gray matter—cortical reorganization	WQ	Clusters with random forest regression. Vector machine regression as a linear method (n).	(+) Left medial temporal cortex *r* = 0.42, *p* < 0.05 (–) Left superior temporal cortex *r* = –0.32, bilateral thalami *r* = –0.049, *p* < 0.05
Knopke et al., 2021, *N*_young50–70_ = 25, *N*_old<70_ = 23	White matter lesions with Fauzekas score	WQ SQ + N	Multiple linear regression analysis with backward elimination (n), df = 24 and 21.	(+) Lesions are a significant predictor of speech perception in quiet in younger group. 27.4%, *p* < 0.05 *(ns)* Older group
Layer et al., 2022, *N* = 17	EEG during visual, auditory and audiovisual “ki” and “ka”	WQ	Pearson correlation with Benjamin Hochberg procedure for multiple comparisons (n).	*(ns)* Left auditory cortex activation and speech perception. *r* = *0.27, p* = *0.29*

For each paper sample size (*N*), neuroimaging method, speech perception outcome measure (WQ, words in quiet; SN, sentences in noise; SQ+N, sentences in quiet and noise); statistical test [including a report of a power analysis (y), yes; (n), no], and key findings are reported [(+), positive significant result; (–) negative significant result; *(ns)*, *non-significant result*]. EEG, electroencephalography; fNIRS, functional near-infrared spectroscopy; MRI, magnetic resonance imaging; PET, positron emission tomography; ROI, region of interest; STG/STS, superior temporal gyrus/sulcus. A more detailed version can be found in Supplementary Table 2.

Six of the ten studies observed activation in the temporal lobe (auditory cortex) in response to visual stimuli and activation in the occipital lobe (visual cortex) in response to auditory stimuli [they used ROI (Regions Of Interest)] (Buckley and Tobey, 2010; Sandmann et al., 2012; Chen et al., 2016, 2017; Kim et al., 2016; Zhou et al., 2018). Buckley and Tobey (2010) did not find any significant correlation between activation in the temporal lobe in response to visual stimuli and word and sentence perception in noise (*r* = 0.1618, *p* = 0.6155*). On the contrary, Sandmann et al. (2012) did find activation in the right temporal cortex evoked by visual stimuli to significantly negatively correlate with word perception in quiet (*r* = –0.75, *p* < 0.05) and positively correlate with sentence perception in noise (*r* = 0.72, *p* < 0.05). Kim et al. (2016), also found that better performers (>60% word score in quiet) showed a significantly smaller P1 amplitude in response to visual stimuli compared to poorer performers (<40% word score) (*p* = 0.002*). Additionally, better performers showed larger P1 amplitudes in the occipital cortex (*p* = 0.013*). Both effects showed a correlation with a word intelligibility test (occipital: *r* = 0.755, *p* = 0.001; temporal: *r* = –0.736, *p* = 0.003*) (Kim et al., 2016). Zhou et al. (2018) confirmed these results and found a significant negative correlation between temporal cortex activation and word perception in quiet and sentences in quiet and noise (*r* = –0.668, *p* = 0.009). Along the same lines, correlations to sentence perception in quiet and noise revealed a higher activation in the visual cortex to be positively correlated, as opposed to higher activation in the auditory cortex induced by visual stimuli (*r* = 0.518, *p* = 0.027). It was found that if the beneficial activation in the visual cortex was higher than the activation in the auditory cortex induced by visual stimuli, speech perception was better (Chen et al., 2016). A follow-up analysis calculated the correlations of the continuous input streams of the different areas. It was found that CI users with significantly higher connectivity for auditory than visual stimuli performed better on a word perception test in quiet (*r* = 0.525, *p* = 0.021), but no correlation was found for a sentence perception test in quiet or noise. This might have facilitated auditory speech perception learning processes by supporting visual cues, such as lip reading (Chen et al., 2017).

Three out of ten studies analyzed whole brain activation in response to auditory, visual and audiovisual stimuli (Strelnikov et al., 2013; Song et al., 2015a; Layer et al., 2022). Strelnikov et al. (2013) also found significant negative correlations of temporal lobe activity with word perception in quiet (rest: *r* = 0.9, visual: *r* = 0.77, audiovisual: *r* = 0.7, *p* < 0.05) and positive correlations with posterior temporal cortex and occipital lobe activation (rest: *r* = 0.9, visual: *r* = 0.8, audiovisual: *r* = 0.5, *p* < 0.05). However, Song et al. (2015a) found a negative correlation between occipital lobe activation and word perception in quiet (left: rho = –0.826, *p* = 0.013, right: rho = –0.777, *p* = 0.019). Similarly, Layer et al. (2022) did not find a correlation between activation in the left temporal cortex in response to audiovisual stimuli with word perception in quiet (*r* = 0.27, *p* = 0.29). While the whole brain was observed in these studies, Strelnikov et al. (2013) found activation in the inferior frontal area to be positively correlated with word perception in quiet (rest: *r* = 0.809, visual: *r* = 0.77, audiovisual: *r* = 0.90, *p* < 0.05). This is in line with results from the previous section “3.1.1 Brain responses to auditory stimuli”. Song et al. (2015a) also observed activation in the right amygdala to be positively correlated with word perception in quiet (rho = –0.888, *p* = 0.008).

Lastly, one paper by Han et al. (2019) measured activity pre-implantation and found a significant negative correlation between activity in the superior occipital gyrus and postoperative word score in quiet (*r* = –0.538, *p* < 0.001), as well as a positive correlation with the dorsolateral and dorsomedial frontal cortex (*r* = 0.595, *p* > 0.001). No significant correlation was found with activity in the auditory pathway areas, the inferior colliculus, and the bilateral superior temporal gyrus.

Another way to consider cortical reorganization, focusing more on altered cortical structure than brain activity, is analyzing gray matter probabilities using Magnetic Resonance Imaging (MRI) pre-implantation. Researchers found that gray matter probability in the left superior middle temporal cortex (*r* = 0.42) and bilateral thalamus (*r* = –0.049, *p* < 0.05) significantly predicted postoperative word recognition in quiet (Sun et al., 2021). Similarly, Knopke et al. (2021) demonstrated that white matter lesions (captured using the Fazekas Score) predicted word perception scores in quiet after implantation in 50–70 year-old CI users, but not in older users. The white matter score explained 27.4% of the speech perception variance in quiet (*p* < 0.05, df = 24 and 21), but was not replicated for a sentence test in noise.

#### 3.1.3. Imaging during “mental auditory tasks” other than auditory/speech perception

A group of studies by Lazard et al. (2010, 2011) and Lazard and Giraud (2017) used “mental auditory tasks” to overcome the negative impact of hearing impairment pre-implantation. The tasks involved imagining words or sounds without auditory input. It was hypothesized that performance on these tasks would involve brain areas similar to the ones involved in auditory processing and therefore show good correlations with speech perception outcomes postoperatively. These studies are summarised below (see Table 3 for an overview):

**TABLE 3:** Overview of included papers studying brain activation during mental auditory tasks.

References, sample size	Method	Speech perception measure	Statistical test, (y/n) indicating a power analysis reported	Key findings
Lazard et al., 2010, *N* = 7	fMRI during a phonological rhyming task and word categorization task–preoperatively	WQ	Multiple regression analysis between fMRI data and phonological performance on a reading task, duration of deafness and hearing loss and word recognition scores (n).	(+) During the phonological task the left frontal, parietal, posterior temporal and occipital cortex (–) During the phonological task the anterior temporal, inferior frontal and right supramarginal gyrus. *p* < 0.001 uncorrected.
			Poorer vs. better performers based on Lafon test *t*-test.	(+) Dorsal regions and bilateral occipital regions more activated in better performers. (–) Bilateral ventral network (anterior temporal lobe, inferior frontal cortex and left temporal occipital junction) and right supramarginal gyrus more activated in poorer performers.
Lazard et al., 2011, *N* = 10	fMRI during a visual imaging task of colors and sounds–preoperatively	WQ	Regression analysis (n).	(+) During sound imagery activity in the left inferior frontal gyrus was positively correlated with speech perception *r* = 0.94, *p* = 0.0001.
			Poorer vs. better performers based on word perception *t*-test.	(+) The dorsal fronto-parietal and occipital regions more activated in better performers. (–) The ventral network (bilateral medial temporal lobes incl hippocampal gyrus) more activated in poorer performers.
Lazard and Giraud, 2017, *N* = 11	fMRI during visual rhyming decision task–preoperatively	WQ	Correlation between occipital-temporal coupling and speech perception (n).	(+) Better performers: left posterior STG/STS (–) Poorer performers: left and right fronto-parietal regions, left visual cortex, right posterior STS, right visual cortex. *p* < 0.001 uncorrected.

For each paper sample size (*N*), neuroimaging method, speech perception outcome measure (WQ, words in quiet), statistical test [including a report of a power analysis (y), yes; (n), no], and key findings are reported [(+), positive significant result, (–), negative significant result; *(ns)*, *non-significant result*]. fMRI, functional magnetic resonance imaging; STG/STS, superior temporal gyrus/sulcus. A more detailed version can be found in Supplementary Table 2.

Lazard et al. (2010) found preoperative imaging data can be used to distinguish future better (>70% word score in quiet) and poorer (<50% word score in quiet) performers based on a rhyming task recruiting phonological strategies during reading. Better performers relied on a dorsal phonological route (dynamic stimulus combination) during a written rhyming task, while poorer performers involved a ventral temporo-frontal route (global) and additionally recruited the right supramarginal gyrus. More specifically, they found a significant positive correlation between brain activation during the phonological task and post-CI word recognition in quiet in the left frontal, parietal, posterior temporal and bilateral occipital cortices. A negative correlation was found in the bilateral anterior temporal, inferior frontal cortex and right supramarginal gyrus. This indicates that poorer performers rely more on semantic information, bypassing the phoneme identification and better performers rely more on visual input (*P* < 0.001 uncorrected).

The same research group correlated preoperative imaging data measured during an auditory imagery task with post-CI word scores in quiet. This showed a decline in activity in the dorsal and frontoparietal cortex and an increase in the ventral cortical regions, right anterior temporal pole and hippocampal gyrus. Activation levels of the right posterior temporal cortex and the left insula were not significantly correlated, but activation levels of the inferior frontal gyrus were positively correlated with word scores in quiet (*r* = 0.94, *p* = 0.0001) (Figure 3C; Lazard et al., 2011).

Lastly, Lazard and Giraud (2017) used a visual phonological rhyming task, including non-words that are pronounced as words, and measured brain activity preoperatively. They correlated this with postoperative word scores in quiet and found that response time on the task (*r* = 0.60, *p* = 0.008) and reorganized connectivity across the bilateral visual, right superior temporal sulcus and the left superior parietal cortex/postcentral gyrus correlated significantly with poorer CI performance (*p* < 0.001). Slower response times were associated with increased activity in the frontoparietal regions and better CI performance. Based on these papers, the group of Lazard concluded that poorer performers use more semantic concepts of sounds instead of phoneme identification, even when not confronted with auditory input. Better performers seemed to be able to utilize additional visual input to support speech perception, as also seen in the studies investigating cross-modal plasticity.

### 3.2. Cognitive tasks

In this review, 31 studies used cognitive tests to assess one or more neurocognitive function(s) and related these outcomes to speech perception outcomes. The studies are described below. Table 4 summarizes time, type of speech perception measurements and related cognitive domain of the papers. Additionally, the sample size and whether a power analysis is reported are noted down. Note that most studies performed cognitive testing postoperatively. If a study performed cognitive assessment preoperatively this will be explicitly mentioned. All speech perception measures were performed postoperatively.

**TABLE 4:** Overview of included papers involving cognitive and language assessments.

References	Time of cognitive assessment		Speech perception measure				Cognitive domain (positive or negative outcome 0/−1/+1)							Sample size	Power reported (y/n)
	**Preoperative**	**Postoperative**	**Words**	**Sentences**	**Quiet**	**Noise**	**Complex attention**	**Executive function**	**Social cognition**	**Learning and memory**	**Perceptual-motor function**	**Language**	**General**		
Collison et al., 2004	–	x	x	–	x	–	–	0	–	–	–	0	–	15	–
Hay-McCutcheon et al., 2005	x	–	x	x	x	x	–	–	–	–	–	–1	–	34	–
Haumann et al., 2012	x	–	x	–	x	–	–	–	–	–	–	0	–	97	–
			–	x	–	x	–	–	–	–	–	1	–		–
Holden et al., 2013	x	–	x	–	–	–	–	0	–	0	–	0	–	92	–
Kaandorp et al., 2015	–	x	x	x	x	x	–	–	–	–	–	0	–	24	–
Finke et al., 2016	–	x	x	x	x	–	–	0	–	–	–	0/1	–	13	–
Moberly et al., 2016b	–	x	–	x	–	x	0	0/−1	–	–	–	–	–	30	–
Hua et al., 2017	–	x	x	–	x	–	0/−1	0/1	–	–	–	–	–	17	*n*
			–	x	–	x	0/1	0	–	–	–	–	–		–
Moberly et al., 2017a	–	x	x	x	x	x	–	0	–	–	–	0	–	30	–
Moberly et al., 2017b	–	x	–	x	x	x	–	0/−1/1	–	–	–	–	–	30	–
Moberly et al., 2017c	–	x	–	x	x	–	–	0/1	–	–	–	–	–	30	–
Kaandorp et al., 2017	–	x	x	x	x	x	–	–1	–	–	–	0/1	–	20	–
Mattingly et al., 2018	–	x	–	x	x	–	–	1	–	–	–	–	–	39	–
Moberly et al., 2018a	–	x	x	x	x	–	–	0/1	–	–	–	1	–	42	–
Moberly et al., 2018c	–	x	x	–	x	–	–	0/1	–	–	–	1	–	34	–
			–	x	x	–	–	0/1	–	–	–	0/1	–		–
Pisoni et al., 2018	–	x	x	–	x	–	–	0	–	0	–	0	–	25	–
			–	x	x	–	–	0/1	–	0/1	–	0	–		–
O’Neill et al., 2019	–	x	–	x	x	–	–	0/1	–	–	–	–	–	30	–
Hillyer et al., 2019	–	x	–	x	x	–	0	0/1	–	–	0	–	–	21	–
Moberly and Reed, 2019	–	x	–	x	x	–	–	0/1	–	–	–	1	–	41	–
Mussoi and Brown, 2019	–	x	–	x	–	x	0	1	–	–	–	–	–	20	–
Dingemanse and Goedegebure, 2019	–	x	–	x	x	–	–	0	–	–	–	–	–	50	–
			–	x	–	x	–	1	–	–	–	–	–		–
Tamati et al., 2020	–	x	–	x	x	–	–	1	–	1	–	–	–	21	–
Kessler et al., 2020	–	x	x	x	x	x	–	0	–	0	–	0	–	21	–
Skidmore et al., 2020	–	x	x	x	x	–	–	0	–	0	0	–	–	40	–
Tinnemore et al., 2020	–	x	–	x	x	–	0	0	–	–	–	–	–	10	–
Wazen et al., 2020	x	–	x	x	x	x	–	–	–	–	–	–	1	40	–
Zhan et al., 2020	x	–	x	x	x	x	–	0/−1/1	–	–	–	–	–	19	–
Bosen et al., 2021	–	x	–	x	x	–	–	1/0	–	–	–	0	–	20	–
Moberly et al., 2021	–	x	x	–	x	–	–	0/−1/1	–	–	–	1	–	18, 17, 16	–
			–	x	x	–	–	0/−1/1	–	–	–	0/1	–		–
Tamati et al., 2021	–	x	–	x	x	–	–	–	–	–	–	1	–	21	–
Tamati and Moberly, 2021	–	x	x	–	x	–	–	0/1	–	–	–	–	–	15	–
Völter et al., 2021	–	x	x	–	x	–	0/1	0/1	–	1	–	1/−1	–	19 + 15	–
Zucca et al., 2022	x	–	x	–	x	–	0/1	0	–	0	0	0	0	15	–
Ray et al., 2022	–	x	–	x	x	x	–	–	–	–	–	1	–	32	*
Walia et al., 2022	–	x	–	x	–	x	–	–	–	–	–	–	1	39	*n*
Luo et al., 2022	–	x	x	x	–	x	–	0/−1/1	–	–	–	–	–	14	–

Overview of the included papers that assess cognitive or language skills before or after implantation in adults and relate this with speech perception outcomes. For each paper it is indicated whether the cognitive or language assessment was performed preoperatively or postoperatively. Additionally it is indicated what speech perception outcome was considered, either words in quiet or noise or sentences in quiet or noise or both. Furthermore, it is shown whether a non-significant result for tests assessing a cognitive domain (0), a positive or relevant relationship (1) or a negative relationship (–1) was found. And lastly for each paper the sample size and if a power analysis is reported (y, yes and sufficient; n, yes and not sufficient for this test) are noted down. *Stated this is a robust approach regarding sample size.

#### 3.2.1. General cognitive measures

Three of the included papers used general (diagnostic) cognitive measures, not specifying which of the cognitive domains were measured by the task. These four more clinical tests, mostly used to detect early signs of Dementia (see Table 5a for an overview), are: (1) The Mini-Mental State Examination (MMSE) which did not significantly correlate with word perception in quiet (*r*^2^ = 0.061, *p* = 0.280, *N* = 15) (Zucca et al., 2022). (2) The Self-Administered Gerocognitive Examination (SAGE), where preoperative screening of cognitive functions significantly positively correlated with word recognition in quiet [*r*^2^(32) = 0.1955, *p* = 0.0025] and sentence perception in quiet [*r*^2^(32) = 0.1564, *p* = 0.0067] and noise [*r*^2^(32) = 0.1543, *p* = 0.007] (Wazen et al., 2020). (3) The Montreal Cognitive Assessment (MoCA), which was included in a multivariate model explaining variance in sentence perception performance in noise. Together with neuronal health measures, MoCA explained 64.5% of this variance (β = 2.06, *p* < 0.05, df = 29) (Walia et al., 2022). (4) The clock drawing test, a short subtest of SAGE, which did not show a significant relationship with word perception in quiet (*r*^2^ = 0.177, *p* = 0.058, *N* = 15) (Zucca et al., 2022).

**TABLE 5:** Overview of included papers (a) using general cognitive measures and (b) studying complex attention.

Cognitive measure	Speech measure	Statistical analysis	Results	References	*N*
**(a) General cognitive measures**					
MSSE	WQ	Mann-Whitney U test, regression analysis	*(ns) p* = *0.545; r^2^* = *0.061*, β = *0.247, p* = *0.280*	Zucca et al., 2022 *	15
SAGE	WQ	Linear correlation	(+) *r*^2^(32) = 0.1955, *p* = 0.0025 language *p* = 0.01, visuospatial *p* = 0.007, executive control *p* = 0.03, memory *p* = 0.02, reasoning *p* = 0.02	Wazen et al., 2020 *	40
	SQ + N	Linear correlation	(+) SQ: *r*^2^(32) = 0.1564, *p* = 0.0067, SN: *r*^2^(32) = 0.1543, *p* = 0.007	Wazen et al., 2020 *	40
MoCA	SN	Simple and multiple linear regression	In a model with a measure of neuronal health (Ecochg-TR) MoCA scores explained 64.5% of the variance β = 2.06 *p* < 0.05, or in interaction with Ecochg-TR β = 0.12 *p* < 0.05.df = 29	Walia et al., 2022	39
Clock drawing test	WQ	Mann-Whitney U test, regression analysis	*(ns) p* = *0.117; r^2^* = *0.177*, β = *0.421, p* = *0.058*	Zucca et al., 2022 *	15
**(b) Complex attention**					
**Attention**					
Leiter-3 attention sustained	SQ	Pearson correlation	*(ns) r* = *0.14*, non-word *r* = *0.29*	Moberly et al., 2016b	30
			*(ns)* Normal*: r* = *0.14*, non-word: *r* = *019*	Moberly et al., 2017b	30
	SN	Pearson correlation	*(ns)* Dyslexia test: *r* = *0.14*, HINT-C*: r* = *0.19*	Moberly et al., 2016b	30
			*(ns) r* = *0.19*	Moberly et al., 2017b	30
WJ-IV letter and number pattern matching, pair cancelation task	SQ	Pearson correlations, controlling for age	*(ns)*	Hillyer et al., 2019	21
ALAcog M3 attentional task	WQ	Rank ANOVA, DFA	(+) Cohen’s d = 1.12, *p* = 0.003 discriminant *r* = 0.50	Völter et al., 2021	34
TMT-B	WQ	Pearson correlations	(–) CI only: *r* = –0.52, *p* < 0.05, corrected for age: *r* = –0.53, *p* < 0.05 Bimodal: *r* = 0.75, *p* > 0.01, corrected for age: *r* = –0.67, *p* < 0.01	Hua et al., 2017	17
		Rank ANOVA	(+) Cohen’s d = 0.96, *p* = 0.018	Völter et al., 2021	
		Mann-Whitney U test, regression analysis	*(ns) p* = *0.087, r^2^* = *0.086*, β = −*0.370, p* = *0.119*	Zucca et al., 2022 *	15
	SN	Pearson correlations	(+) *r* = 0.55, *p* > 0.05 *(ns)* corrected for age *r* = 0.46	Hua et al., 2017	17
**Processing speed**					
WJ-IV letter and number pattern matching, pair cancelation task → see attention					
WAIS-III symbol search test	SN	Zero-order correlations	*(ns)*	Mussoi and Brown, 2019	20
NIH toolbox pattern comparison processing speed test	SQ	Generalized linear mixed-effects regression analysis	(+) *p* = 0.006 (for normal hearing, but no interaction, so same results for CI)	Tinnemore et al., 2020	10
WAIS-III coding test	SN	Zero-order correlations	*(ns)*	Mussoi and Brown, 2019	20
WJ-IV numbers reversed and pictures test	SQ	Pearson correlations, controlling for age	*(ns)*	Hillyer et al., 2019	21
TMT-A	WQ	Pearson correlations	*(ns)* CI only: *r* = −*0.27*, corrected for age: *r* = −*0.53* (*-*) Bimodal *r* = −0.60, *p* > 0.05 (*ns)* corrected for age: *r* = −*0.48*	Hua et al., 2017	17
		Rank ANOVA	*(ns) Cohen’s d = 0.8, p = 0.053*	Völter et al., 2021	34
		Mann-Whitney U test, regression analysis	(+) *r*^2^ = 0.236, β = –0.486, *p* = 0.035, *(ns) p* = *0.115*	Zucca et al., 2022 *	15
	SN	Pearson correlations	*(ns) r* = *0.19*, corrected for age: *r* = *0.16*	Hua et al., 2017	17

Overview of included papers using general cognitive measures and assessing complex attention. The task, speech perception outcome measure (W, words; S, sentence; Q, quiet; N, noise), statistical analysis, key finding [(+), positive significant result; (–) negative significant result; *(ns)*, *non-significant result*], reference and sample size (N) are reported. DFA, discriminant factor analysis; HINT, Hearing in Noise Test; MoCA, Montreal Cognitive Assessment; NIH, National Institutes of Health; SAGE, Self-administered Gerocognitive Examination, TMT-A/B, Trail Making Task version A or B; WAIS-III, Wechsler Adult intelligence scale III, WJ-IV, Woodcock-Johnson IV, *Cognitive measure preoperatively. A more detailed version can be found in Supplementary Table 4.

#### 3.2.2. Complex attention

##### 3.2.2.1. Attention

Attention is a state of selective awareness by which a single stimulus is selected for enhanced processing. Tasks assessing attention mainly involve target selection. For the papers included in this review, the “Leiter-3 sustained attention task,” “Woodcock-Johnson IV (WJ-IV) letter and number pattern matching task and the pair cancelation task”, and the “ALAcog M3 attentional task” are used (see Table 5b for an overview). These tests involve targets like figures, letters, numbers or repeated patterns on paper among a set of distractors (Moberly et al., 2016b, 2017b; Hillyer et al., 2019; Völter et al., 2021). Of these tasks, only performance on the ALAcog attentional task was significantly different between better and poorer performers on a word test in quiet (Cohen’s d = 1.12, *p* = 0.003) (Völter et al., 2021). The other tests showed no significant relationship with sentences in quiet or noise (Moberly et al., 2016b, 2017b; Hillyer et al., 2019).

Another task used in several studies to investigate attention is the Trail Making Task B (TMT-B). For this task the participant has to draw a “trail” of consecutive numbers and letters: 1-A-2-B-3-C etc. Across the studies that this task was used in, the results were inconsistent showing both significant negative and positive correlations with words in quiet (WQ: CI only: *r* = –0.52, *p* < 0.05, corrected for age: *r* = –0.53, *p* < 0.05, bimodal *r* = 0.75, *p* > 0.01 corrected for age: *r* = –0.67, *p* < 0.01; Cohen’s d = 0.96, *p* = 0.018) and positive (*r* = 0.55, *p* > 0.05) or non-significant correlations (when measured preoperatively: *p* = 0.087) with sentences in noise (Hua et al., 2017; Völter et al., 2021; Zucca et al., 2022).

##### 3.2.2.2. Processing speed

Processing speed is the time required to complete a mental task (Kail and Salthouse, 1994). To test this, one task is used that also assesses attention: the “WJ-IV letter and number pattern matching task and the pair cancelation task.” Furthermore, the “Wechsler Adult Intelligence Scale III (WAIS-III) symbol search.” the “NIH toolbox pattern comparison processing speed test”, the “WAIS-III coding test” and the “WJ-IV numbers reversed test” are used (see Table 5b for an overview) (Hillyer et al., 2019; Mussoi and Brown, 2019; Tinnemore et al., 2020). Of all of these tests only the pattern comparison test showed a significant relationship with sentences in quiet (*p* = 0.006) (Tinnemore et al., 2020).

Another task used to measure processing speed is the TMT-A. For this task the participant has to draw a trail from 1 to 25. When measured preoperatively, performance on TMT-A was a significant factor in regression analysis for words in quiet postoperatively (*r*^2^ = 0.236, *p* = 0.035) (Zucca et al., 2022). However, when TMT-A was measured postoperatively, performance was in general not significantly related to perception of words in quiet or sentences in noise (WQ: CI only non-significant *r* = –0.27, Bimodal *r* = –0.60, *p* > 0.05, corrected for age non-significant *r* = –0.48, SN *r* = 0.19; Cohen’s d = 0.8, *p* = 0.053) (Hua et al., 2017; Völter et al., 2021).

#### 3.2.3. Executive function

##### 3.2.3.1. Non-verbal intelligence

Non-verbal intelligence is an executive function that relates to thinking skills and problem-solving abilities that do not require language. “Ravens progressive matrices task,” the “WAIS-III matrix reasoning test,” “Leiter-3 visual pattern task,” “Test of Non-verbal Intelligence–3 (TONI-3) pointing to pictures” and the “Leiter-3 figure ground and form completion” were used to measure this (see Table 6 for an overview).

**TABLE 6:** Overview of included papers studying non-verbal intelligence.

Cognitive measure	Speech measure	Statistical analysis	Results	References	*N*
**Non-verbal intelligence**					
Ravens Progressive Matrices (RPM)	WQ	Pearson correlations	*(ns) r* = *0.196, p* = *0.421*	Zhan et al., 2020 *	19
			(+) Later in time for hard words: *r* = 0.68, *p* = 0.009 *(ns)* Q1 easy*: r* = −*0.09, p* = *0.372* hard*: r* = *0.32, p* = *0.180* Q4 easy: *r* = *0.14, p* = *0.371* hard*: r* = *0.47, p* = *0.048* TA easy*: r* = *0.26, p* = *0.371* hard: *r* = −*0.42, p* = *0.116*	Tamati and Moberly, 2021	15
		Partial correlation analysis, controlled for age	(+) *r* = 0.35, *p* < 0.05	Moberly et al., 2018a	42
		Correlational and regression analysis	(+) *r* = 0.64, *p* < 0.05, and additional value in model	Pisoni et al., 2018	25
		Linear regression analysis	(+) *r*^2^ = 0.325, *p* < 0.001, β = 0.570 (also mediated by age)	Moberly et al., 2018c	34
		ANOVA, spearmans rank-order correlations Divided in 3 groups	(+) Low-smrt group: rho = 0.52, *p* = 0.02 *(ns)* intermediate-smrt: *rho* = *0.33, p* = *0.10*, high-smrt*: rho* = *0.42, p* = *0.05*	Moberly et al., 2021	51
	SQ	Pearson correlations	*(ns) r* = *0.319, p* = *0.086*	O’Neill et al., 2019	30
			(+) PRESTO words: *r* = 0.41, *p* < 0.01, PRESTO sentence *r* = 0.47, *p* < 0.01, Harvard words *r* = 0.35, *p* < 0.05, Harvard sentence *r* = 0.46, *p* < 0.01	Mattingly et al., 2018	39
			*(ns)* PRESTO: *r* = *0.295, p* = *0.221*, Harvard standard: *r* = *0.208, p* = *0.392*, anomalous*: r* = *0.212, p* = *0.383*	Zhan et al., 2020 *	19
		Pearson correlations, DFA	Matrix coefficient = 0.35, rank 2, df = 10	Tamati et al., 2020	21
		Partial correlation analysis	(+) Harvard: *r*(26) = 0.53, *p* < 0.01, *(ns)* PRESTO	Moberly et al., 2017c	30
		Partial correlation analysis, controlled for age	(+) PRESTO words: *r* = 0.45, *p* < 0.01, PRESTO sentences: *r* = 0.47, *p* < 0.01, Harvard sentences *r* = 0.39, *p* < 0.05	Moberly et al., 2018a	42
		ANOVA, spearmans rank-order correlations Divided in 3 groups	(+) PRESTO High-smrt group: *r* = 0.52, *p* = 0.01 *(ns)* Harvard, low-smrt: *rho* = *0.30, p* = *0.13*, intermediate-smrt: *rho* = *0.44, p* = *0.05*, high-smrt*: rho* = *0.22, p* = *0.19* PRESTO: low-smrt: *rho* = *0.26, p* = *0.17*, intermediate-smrt: *rho* = *0.35, p* = *0.09.*	Moberly et al., 2021	51
		Correlational and regression analysis	(+) Harvard words: *r* = 0.71, *p* < 0.05, sentences: *r* = 0.60, *p* < 0.05 PRESTO words: *r* = 0.62, *p* < 0.05, PRESTO sentences: *r* = 0.68, *p* < 0.05, and additional value in model	Pisoni et al., 2018	25
		Linear regression analysis	(+) Harvard words: *r*^2^ = 0.291, β = 0.540, *p* = 0.001 PRESTO words: *r*^2^ = 0.357, β = 0.598, *p* < 0.001	Moberly et al., 2018c	34
		Blockwise multiple linear regression analysis	(+) Adding ravens to predict anomalous sentences β = 0.421, p = 0.08 *(ns)* meaningful: β = −*0.141*, *p* = *0.173*, df = 32	Moberly and Reed, 2019	41
	SQ WQ	Partial least squares regression	*(ns)*	Skidmore et al., 2020	40
	SQ + N	Pearson correlations	*(ns)* SQ*: r* = *0.253, p* = *0.295* SN*: r* = *0.167 p* = *0.493*	Zhan et al., 2020 *	19
WAIS-III matrix reasoning test	WQ	Non-parametric correlation and principal component measures.	*(ns)* when corrected for age	Holden et al., 2013 *	92
Leiter-3 visual pattern test	SQ + N	Pearson correlation	*(ns)* Dyslexia test: *r* = *0.33*, HINT-C: *r* = *0.26*, non-word: *r* = *0.33*	Moberly et al., 2016b	30
TONI-3 pointing pictures	WQ	Pearson correlations	*(ns) r* = *0.155*	Collison et al., 2004	15
Leiter-3 figure ground	SQ + N	Pearson correlation	*(ns)* Dyslexia test: *r* = *0.15*, HINT-C: *r* = *0.13*, non-word: *r* = *0.15*	Moberly et al., 2016b	30
Leiter-3 form completion	SQ + N	Pearson correlation	*(ns)* Dyslexia test: *r* = −*0.09*, HINT-C: *r* = −*0.16*, non-word: *r* = −*0.09*	Moberly et al., 2016b	30

Overview of included papers using general cognitive measures and assessing complex attention. The task, speech perception outcome measure (W, words; S, sentence; Q, quiet, N, noise), statistical, key finding [(+), positive significant result; (–) negative significant result; *(ns)*, *non-significant result*], reference and sample size (N) are reported. DFA, discriminant factor analysis, HINT, Hearing in Noise Test; TONI, Test of Non-verbal Intelligence; WAIS-III, Wechsler Adult intelligence scale III; *Cognitive measure preoperatively. A more detailed version can be found in Supplementary Table 4.

In this review, the Ravens task is used most frequently to measure non-verbal intelligence (Moberly et al., 2017c, 2018a,c, 2021; Mattingly et al., 2018; Pisoni et al., 2018; Moberly and Reed, 2019; O’Neill et al., 2019; Skidmore et al., 2020; Zhan et al., 2020; Tamati and Moberly, 2021; Tamati et al., 2021). The task is to pick the piece that fits within the pattern of a visual geometric matrix. A significant relationship between performance on the Ravens task and word perception in quiet was found in five out of six included papers (*r* = 0.35, *p* < 0.05; *r*^2^ = 0.325, *p* < 0.001; *r*^2^ = 0.64, *p* < 0.05; *r* = 0.196, *p* = 0.421) (Moberly et al., 2018a,c, 2021; Pisoni et al., 2018; Zhan et al., 2020; Tamati et al., 2021). However, it should be noted, that in a study by Tamati and Moberly (2021) this positive correlation was found after 10 trials of word perception, when the listener was adapted to the talker (*r* = 0.68, *p* = 0.009, df = 10). In a study by Moberly et al. (2021) this positive correlation was found for participants with low auditory sensitivity (*r* = 0.52, *p* = 0.02) (where auditory sensitivity was determined by Spectral-Temporally Modulated Ripple Test performance). Eight out of ten studies (some using the same group of participants) using this task reported a significant relationship between performance and sentence perception in quiet [*r*(26) = 0.53, *p* < 0.01; PRESTO words: *r* = 0.45, *p* < 0.01; *r* = 0.62, *p* < 0.05; *r* = 0.41, *p* < 0.01, and sentences: *r* = 0.47, *p* < 0.01; *r* = 0.68, *p* < 0.05; *r* = 0.47, *p* < 0.01; Harvard words: *r* = 0.71, *p* < 0.05; *r* = 0.35, *p* < 0.05 and sentences *r* = 0.39, *p* < 0.05; *r* = 0.60, *p* < 0.05; *r* = 0.46, *p* < 0.01; Adding Ravens score to a blockwise multiple linear regression analysis to predict anomalous sentences *p* = 0.08, df = 32] (Moberly et al., 2017c, 2018a,b, 2021; Mattingly et al., 2018; Pisoni et al., 2018; Moberly and Reed, 2019; O’Neill et al., 2019; Skidmore et al., 2020; Zhan et al., 2020). For one of these studies, Ravens task performance discriminated highly between two groups of better and poorer performers on sentences in quiet (Matrix coefficient = 0.35, rank 2, df = 10) (Tamati et al., 2020). In another study, Moberly et al. (2021) found that there was only a significant positive correlation between Ravens score and sentence perception in participants with high auditory sensitivity (*r* = 0.52, *p* = 0.01). Lastly, in another paper they found that there was only a predictive value of Ravens score with anomalous sentences and not meaningful sentences (*p* = 0.008, df = 32) (Moberly and Reed, 2019). The other tasks used to assess non-verbal intelligence did not show any significant results when related to speech perception performance (*r* = –0.16 to 0.33) (Collison et al., 2004; Holden et al., 2013; Moberly et al., 2016b).

##### 3.2.3.2. Working memory

Working memory is a buffer that holds memories accessible while a task is performed (Breedlove and Watson, 2013). It has been suggested that a linear relationship exists between the ambiguity of the speech stimulus and the working memory capacity needed, to decide what words were perceived (Rönnberg, 2003). Working memory can be assessed in different ways and using different modalities; visual (see Table 7 for an overview), auditory, audio-visual and verbal (see Table 8 for an overview).

**TABLE 7:** Overview of included papers studying visual working memory.

Cognitive measure	Speech measure	Statistical analysis	Results	References	*N*
**Visual working memory**					
Visual digit span	WQ	Pearson correlations	*(ns) r* = *0.269, p* = *0.265*	Zhan et al., 2020 *	19
			*(ns)* Q1 easy: *r* = −*0.15, p* = *0.350* hard*: r* = *0.19, p* = *0.246* Q4 easy: *r* = −*0.14, p* = *0.371* hard *r* = *0.17, p* = *0.269* TA easy: *r* = −*0.04 p* = *0.444* hard *r* = *0.04, p* = *0.448*	Tamati and Moberly, 2021	15
		Partial correlation analysis, controlled for age	*(ns) r* = *0.09*	Moberly et al., 2018a	42
		Linear regression analysis	*(ns) r*^2^ = *0.005*, β = *0.068, p* = *0.704*	Moberly et al., 2018c	34
		ANOVA, spearmans rank-order correlations divided in three groups	*(ns)* Low-smrt: *rho* = −*0.18, p* = *0.25*, intermediate-smrt: *rho* = *0.19, p* = *0.23*, high-smrt*: rho* = −*0.01, p* = *0.49*	Moberly et al., 2021	51
	SQ	Pearson correlations	*(ns)* Harvard standard: *r* = *0.333, p* = *0.163*, anomalous *r* = *0.232, p* = *0.339*, PRESTO*: r* = *0.418 p* = *0.075*	Zhan et al., 2020 *	19
			(+) Controlling for age: CI only *r* = 0.539, *p* = 0.016	Hillyer et al., 2019	21
		Partial correlation analysis	(+) Harvard *r*(26) = 0.40, *p* = 0.035, *(ns)* PRESTO	Moberly et al., 2017c	30
			*(ns)* Controlled for age*:* Harvard words *r* = *0.12*, sentences: *r* = *0.26* PRESTO words: *0.08* sentences: *r* = *0.17*	Moberly et al., 2018a	42
		Pearson correlations, DFA	Matrix coefficient = 0.00, rank 10, df = 10	Tamati et al., 2020	21
		Linear regression analysis	*(ns)* Harvard:*r*^2^ = *0.010*,β = *0.101, p* = *0.576*, PRESTO: *r*^2^ = *0.003*,β = *0.057, p* = *0.751*,	Moberly et al., 2018c	34
		Blockwise multiple linear regression analysis	*(ns)* Meaningful: β = −*0.010, p* = *0.910*, anomalous: β = *0.335, p* = *0.740*, df = 32	Moberly and Reed, 2019	41
		ANOVA, spearmans rank-order correlations divided in 3 groups	(+) PRESTO, Intermediate-smrt: rho = 0.49, p = 0.03, *(ns)* Harvard*:* low-smrt: *rho* = *0.11, p* = *0.34*, intermediate-smrt: *rho* = *0.44, p* = *0.05*, high-smrt*: rho* = −*0.05, p* = *0.42* PRESTO: low-smrt: *rho* = −*0.07, p* = *0.40*, high-smrt*: rho* = −*0.03, p* = *0.46*	Moberly et al., 2021	51
	WQ SQ	Partial least squares regression	*(ns)*	Skidmore et al., 2020	40
	SQ + N	Pearson correlations	*(ns)* SQ: *r* = *0.309, p* = *0.198* SN*: r* = *0.44, p* = *0.057*	Zhan et al., 2020 *	19
WJ-IV numbers reversed test and pictures→see processing speed Table 5					
Leiter-3 forward and reverse memory	SQ	Pearson correlation	*(ns) r* = *0.23*, non-word: *r* = *0.14*	Moberly et al., 2017b	30
			*(ns)* Non-words: *r* = *0.23, r* = *0.20*	Moberly et al., 2016b	30
	SN	Pearson correlation	*(ns)* HINT-C*: r* = *0.23, r* = *0.20*, Dyslexia: *r* = *0.23, r* = −*0.28*	Moberly et al., 2016b	30
			*(ns) r* = *0.13*	Moberly et al., 2017b	30
WAIS-III visual object span	WQ	Pearson correlations	*(ns) r* = *0.196, p* = *0.421*	Zhan et al., 2020 *	19
	SQ	Partial correlation analysis	*(ns)*, df = 26	Moberly et al., 2017c	30
		Pearson correlations	*(ns)* Harvard standard: *r* = *0.253, p* = *0.296*, anomalous *r* = *0.125, p* = *0.609*, PRESTO*: r* = *0.241, p* = *0.321*	Zhan et al., 2020 *	19
	WQ SQ	Partial least squares regression	*(ns)*	Skidmore et al., 2020	40
	SQ + N	Pearson correlations	*(ns)* SQ: *r* = *0.355, p* = *0.136* SN: *r* = *0.426, p* = *0.069*	Zhan et al., 2020 *	19
Visual letter span task	WN + SN	Pearson correlations	*(ns)* WN: *r* = −*0.27, p* = *0.35*, SN: *r* = −*0.11, p* = *0.71*	Luo et al., 2022	14
WAIS-III visual symbol span	WQ	Pearson correlations	(+) *r* = 0.599 *p* = 0.007	Zhan et al., 2020 *	19
	SQ	Partial correlation analysis	*(ns)*	Moberly et al., 2017c	30
		Pearson correlations	(+) Harvard standard: *r* = 0.541, *p* = 0.017, *(ns)* Harvard anomalous *r* = *0.345, p* = *0.148*, PRESTO*: r* = *0.443, p* = *0.057*	Zhan et al., 2020 *	19
	WQ SQ	Patial least squares regression	*(ns)*	Skidmore et al., 2020	40
	SQ + N	Pearson correlations	(+) SQ: *r* = 0.504, *p* = 0.028, SN: *r* = 0.486, *p* = 0.035	Zhan et al., 2020 *	19
Alacog 2-back test	WQ	Rank ANOVA, DFA	*(ns) Cohen’s d* = *0.5, p* = *0.22*	Völter et al., 2021	34
Alacog OSPAN	WQ	Rank ANOVA, DFA	(+) Cohen’s *d* = 1.01, *p* = 0.0068	Völter et al., 2021	34

Overview of included papers assessing visual working memory. The task, speech perception outcome measure (W, words; S, sentence; Q, quiet; N, noise), statistical analysis, key finding [(+), positive significant result; (–), negative significant result; *(ns)*, *non-significant result*], reference and sample size (N) are reported. DFA, discriminant factor analysis, OSPAN, Operation Span, WAIS-III, Wechsler Adult intelligence scale III; WJ-IV, Woodcock-Johnson IV; *Cognitive measure preoperatively. A more detailed version can be found in Supplementary Table 4.

**TABLE 8:** Overview of included papers studying auditory, audio-visual and verbal working memory.

Cognitive measure	Speech measure	Statistical analysis	Results	References	*N*
**Auditory working memory**					
Auditory digit span	WQ	Non-parametric correlation and principal component measures. Divided in six groups	*(ns)* when corrected for age	Holden et al., 2013 *	92
	WN	Pearson correlations	*(ns) r* = −*0.27, p* = *0.35*	Luo et al., 2022	14
	SQ	Pearson correlation	(+) *r* = 0.51, *p* = 0.03, after correcting for auditory resolution *(ns) r* = *0.39, p* = *0.08*	Bosen et al., 2021	20
	WQ SQ + N	Correlation analysis	*(ns)*, df = 28	Moberly et al., 2017a	30
	SN	Pearson correlations	*(ns) r* = −*0.21, p* = *0.48*	Luo et al., 2022	14
**Audio-visual working memory**					
Audio-visual digit span	WQ	Mann-Whitney U test, regression analysis	*(ns)* forward: *p* = *0.199; r*^2^ = *0.003*, β = *0.051*, *p* = *0.826*, backward: *p* = *0.382; r^2^* = *0.036*, β = *0.190, p* = *0.410*	Zucca et al., 2022 *	15
	SN	Zero-order correlations	(+) *r* = 0.573, *p* = 0.018	Mussoi and Brown, 2019	20
Cued modality working memory task	WN	Pearson correlations	(–) Auditory cued working memory: *r* = –0.54, *p* = 0.0047 Auditory uncued working memory: *r* = –0.60, *p* = 0.02 → after correcting for auditory resolution: *r* = –0.65, *p* = 0.03	Luo et al., 2022	14
	SN	Pearson correlations	(–) Auditory cued working memory: *r* = –0.66, *p* = 0.01 Auditory uncued working memory: *r* = –0.54, *p* = 0.0045	Luo et al., 2022	14
**Verbal working memory**				
Listening span	SQ + N	Pearson correlation	(+) SQ: *r* = 0.64, *p* < 0.01 non-word *r* = 0.68, *p* < 0.01, SN: *r* = 0.57, *p* < 0.01	Moberly et al., 2017b	30
Reading span	WQ	Pearson correlation also corrected for age	(–) Bimodal *r* = –0.71, *p* > 0.01, corrected for age *r* = 0.70, *p* < 0.01, *(ns)* CI only: *r* = *0.44*, corrected for age: *r* = *0.42*	Hua et al., 2017	17
		Spearman correlation coefficients	*(ns) rho* = *0.09, p* = *0.58*	Dingemanse and Goedegebure, 2019	50
	WN	Pearson correlations	*(ns) r* = −*0.05, p* = *0.86*	Luo et al., 2022	14
	SQ	Pearson correlation	*(ns) S*hort: *r* = −*0.3* non-word: *r* = −*0.02*	Moberly et al., 2017b	30
			(+) *r* = 0.430, *p* = 0.018	O’Neill et al., 2019	30
	SN	Pearson correlation also corrected for age	*(ns)*: *r* = −*0.48*, corrected for age: *r* = −*0.44*	Hua et al., 2017	17
		Pearson correlation	*(ns) r* = *0.1*	Moberly et al., 2017b	30
	WQ + N SN	Correlation analysis, regression analysis	(–) SN: *r* = –0.59, *p* = 0.006, SRTdiff: *r* = –0.57, *p* = 0.009, explained additional 46% *(ns)* WQ: *r* = *0.03*, WN: *r* = −*0.26*	Kaandorp et al., 2017	20
	SQ + N	Spearman correlation coefficients	(+) Words: *r* = 0.37, *p* = 0.011, Sentence: *r* = 0.38, *p* = 0.009	Dingemanse and Goedegebure, 2019	50
	SN	Pearson correlations	*(ns) r* = −*0.03, p* = *0.91*	Luo et al., 2022	14
SicSpan	WQ	Correlation analysis	*(ns)*, df = 11	Finke et al., 2016	13
	SQ	Correlation analysis	*(ns)*, df = 11	Finke et al., 2016	13
	SN	Independent *T*-test	*(ns)*	Kessler et al., 2020	21

Overview of included papers using assessing auditory, audio-visual, and verbal working memory. The task, speech perception outcome measure (W, words; S, sentence; Q, quiet; N, noise), statistical analysis, key finding [(+), positive significant result; (–), negative significant result; *(ns)*, *non-significant result*), reference and sample size (N) are reported. SicSpan, Size comparison Span; SRT, sound reception threshold, WAIS-III, Wechsler Adult intelligence scale III; *Cognitive measure preoperatively. A more detailed version can be found in Supplementary Table 4.

###### 3.2.3.2.1. Visual working memory

The “visual digit span task,” “Leiter-3 forward and reversed memory test, letters, and symbols,” “ALAcog 2-back test” and “Operation Span” (OSPAN) are used to assess visual working memory (Moberly et al., 2016b, 2017c, 2018c, 2021; Mattingly et al., 2018; Hillyer et al., 2019; Moberly and Reed, 2019; Skidmore et al., 2020; Tamati et al., 2020; Zhan et al., 2020; Tamati and Moberly, 2021; Völter et al., 2021; Luo et al., 2022).

The most used in the included literature is the visual digit span. Scores on this task showed no significant correlations with word perception in quiet (*r* = –0.14 to 0.448, *p* = 0.32–0.704) (Moberly et al., 2018a,c, 2021; Pisoni et al., 2018; Skidmore et al., 2020; Zhan et al., 2020; Tamati and Moberly, 2021). For sentence perception in quiet and noise, three out of nine papers found a significant correlation (Moberly et al., 2017c, 2018a,c, 2021; Hillyer et al., 2019; Moberly and Reed, 2019; Skidmore et al., 2020; Tamati et al., 2020; Zhan et al., 2020). More specifically, Moberly et al. (2017c) found a positive correlation with one of two sentence perception tasks in quiet [*r*(26) = 0.40, *p* = 0.035] and Hillyer et al. (2019) a positive correlation when corrected for age (*r* = 0.539, *p* = 0.016). Furthermore, digit span did not significantly discriminate between better and poorer performers on sentence perception in quiet (Matrix coefficient = 0.00, rank 10, df = 10) (Tamati et al., 2020). Lastly, Moberly et al. (2021), found a positive correlation with one of two sentence perception tasks in quiet for participants with an intermediate degree of auditory sensitivity (rho = 0.49, *p* = 0.03).

For similar span tests using pictures or objects, like in the forward and reversed memory test, letters, and symbols, performance showed a significant relationship with word and sentence perception in quiet and noise in one of seven papers (Moberly et al., 2016b, 2017c; Hillyer et al., 2019; Skidmore et al., 2020; Zhan et al., 2020; Luo et al., 2022). This paper showed a positive relationship between symbol and object span measured preoperatively and word perception in quiet and sentence perception in quiet and noise postoperatively (words: *r* = 0.599, *p* = 0.007, sentences in quiet *r* = 0.504, *p* = 0.028 and noise: *r* = 0.486, *p* = 0.035) (Zhan et al., 2020).

Lastly, the OPSAN and 2-back task were applied in one paper each. Performance on the 2-back did not differ significantly between better and poorer performers on a word task in quiet (Cohen’s d = 0.5, *p* = 0.22), but the OSPAN score was significantly worse for poorer performers (Cohen’s d = 1.01, *p* = 0.0068) (Völter et al., 2021).

###### 3.2.3.2.2. Auditory working memory

Similar tasks are used to measure working memory capacity with auditory stimuli instead of visual stimuli (Table 8). The digit span task is used in four of the included studies (r = –0.27, p = 0.35) (Holden et al., 2013; Moberly et al., 2017a; Bosen et al., 2021; Luo et al., 2022). Scores on these tasks were not significantly correlated to words nor sentences in quiet and noise when administered preimplantation (measured once) or postimplantation (Holden et al., 2013; Moberly et al., 2017b). Only one paper reported a significant correlation with sentence perception in quiet, but this effect disappeared when corrected for auditory sensitivity based on spectral or temporal resolution thresholds (r = 0.51, p = 0.03, after correcting for auditory resolution: r = 0.39, p = 0.08) (Bosen et al., 2021).

###### 3.2.3.2.3. Audio-visual working memory

Lastly, stimuli can be presented in the auditory and visual modality at the same time (Hillyer et al., 2019; Mussoi and Brown, 2019; Zucca et al., 2022; Table 8). The audiovisual digit span test was applied in three included studies, with no significant results when related to words in quiet (e.g., when measured preoperatively forward: r^2^ = 0.003, p = 0.826, backward: r^2^ = 0.036, p = 0.410), but a significant positive correlation with sentences in quiet (r = 0.539 p = 0.016) and noise (r = 0.573, p = 0.018) (Hillyer et al., 2019; Mussoi and Brown, 2019; Zucca et al., 2022).

Interestingly, Luo et al. (2022) used a cued modality working memory task, where participants needed to remember auditory digits and visual letters and recall one of them or both. In the cued conditions they were instructed beforehand what they needed to recall after the stimuli were presented, but no instruction was given in the uncued condition. They found that for both the cued and uncued auditory condition, the score on the task was significantly negatively correlated with sentence (cued: *r* = –0.66, *p* = 0.01, uncued: *r* = –0.54, *p* = 0.045) and word perception in noise (cued: *r* = –0.54, *p* = 0.047, uncued: *r* = –0.60, *p* = 0.02). However, after correcting for spectral and temporal resolution, only the significant negative correlation between auditory uncued performance on the working memory task and word perception in noise remained (*r* = –0.65, *p* = 0.03). The authors suggest this might be because the same underlying strategies are used, and because top-down correction using semantic information is not possible, unlike for sentence perception and cued working memory. However, this paper had small sample sizes and results should be interpreted with caution (Luo et al., 2022).

###### 3.2.3.2.4. Verbal working memory

Other working memory tasks involve more language perception skills. These measures are thought to assess verbal working memory more specifically, using both auditory and visual stimuli. Examples of such tasks are the “Reading Span task,” the “Size Comparison Span” (SicSpan) task and the “Listening span task” (Table 8; Finke et al., 2016; Hua et al., 2017; Kaandorp et al., 2017; Moberly et al., 2017b; Dingemanse and Goedegebure, 2019; O’Neill et al., 2019; Kessler et al., 2020). For the Reading or Listening span, participants must decide for each sentence they see or hear whether the sentence is semantically true or false, while retaining items that are presented in memory and recalling them after the true/false task.

Reading span was used most frequently in the included papers. For these studies, performance did not significantly correlate with word perception scores in quiet and noise in three papers (Kaandorp et al., 2017; Dingemanse and Goedegebure, 2019; Luo et al., 2022). A significant positive correlation was, however, found for bimodal listening to words in quiet (*r* = 0.71, *p* > 0.01) (Hua et al., 2017). In two out of six included papers there was a significant positive correlation between Reading span and sentence perception in quiet or noise (*r* = 0.430, *p* = 0.018; *r* = 0.38, *p* = 0.009) (Hua et al., 2017; Moberly et al., 2017b; Dingemanse and Goedegebure, 2019; O’Neill et al., 2019; Luo et al., 2022) and one paper showed a significant negative correlation (*r* = –0.57, *p* = 0.009) (Kaandorp et al., 2017).

Furthermore, only one paper implemented the Listening span and found performance to be significantly positively correlated with sentence perception in quiet and noise (quiet: *r* = 0.64, non-word quiet: *r* = 0.68, noise: *r* = 0.57, *p* < 0.01) (Moberly et al., 2017b). For SicSpan the two included studies found no significant relationship with sentence or word perception in quiet and noise (Finke et al., 2016; Kessler et al., 2020).

##### 3.2.3.3. Cognitive inhibition

Cognitive inhibition is the ability to suppress goal-irrelevant information. For example, being able to ignore background noise or lexical competitors. In the included papers two tasks were used to assess inhibitory control: the “Flanker task” and the “Stroop task” (see Table 9 for an overview). Both tasks contain congruent, incongruent, and neutral conditions. In the congruent condition, the participant must respond to a target where the rest of the properties of the trial are aligned with the required response. In the incongruent condition, the participant must respond to a target where the rest of the properties of the trial are opposite to the required response and in the neutral condition the rest of properties of the trial do not have the ability to evoke a response conflict (Zelazo et al., 2014; Knight and Heinrich, 2017).

**TABLE 9:** Overview of included papers studying cognitive inhibition and flexibility.

Cognitive measure	Speech measure	Statistical analysis	Results	References	*N*
**Cognitive inhibition**				
Stroop task	WQ	Pearson correlations	*(ns) r* = −*0.455, p* = *0.058*	Zhan et al., 2020 *	19
			(–) Later in time for hard words: *r* = –0.50, *p* = 0.044 *(ns)* Q1: easy *r* = −*0.22, p* = *0.317* hard *r* = −*0.27, p* = *0.197* Q4: *easy r* = −*0.05, p* = *0.430* TA: easy *r* = *0.14 p* = *0.371* hard *r* = −*0.58 p* = *0.072*	Tamati and Moberly, 2021	15
		Partial correlation analysis, controlled for age	*(ns) r* = *0.12*	Moberly et al., 2018a	42
		Linear regression analysis	*(ns) r*^2^ = *0.056, p* = *0.108*	Moberly et al., 2018c	34
		ANOVA, spearmans rank-order correlations divided in 3 groups	(–) High-smrt: rho = –0.49, *p* = 0.02, *(ns)* low-smrt: *rho* = *0.31, p* = *0.12*, intermediate-smrt: *rho* = *0.09, p* = *0.36*	Moberly et al., 2021	51
	SQ	Pearson correlations	*(ns)* Incongruent: harvard: standard *r* = −*0.321, p* = *0.193*, anomalous: *r* = −*0.319, p* = *0.197*, PRESTO *r* = −*0.301, p* = *0.224*	Zhan et al., 2020 *	19
			(–) Incongruent: non-word *r* = –0.43, *p* < 0.05 *(ns)* congruent: *r* = −*0.28*	Moberly et al., 2016b	30
			(–) Real words: *r* = –0.41, *p* < 0.05, non-words: *r* = –0.43, *p* < 0.05	Moberly et al., 2017b	30
		Partial correlation analysis, controlled for age	*(ns)* Harvard: words *r* = –*0.13*, sentences *r* = –*0.23*, PRESTO: words *r* = –*0.05*, sentences *r* = –*0.12*	Moberly et al., 2018a	42
		Pearson correlations, DFA	Control: matrix coefficient = –0.08, rank 7 Interference: matrix coefficient = 0.06, rank 8, df = 10	Tamati et al., 2020	10
		Linear regression analysis	*(ns)* Harvard: *r*^2^ = −*0.085, p* = *0.099*, PRESTO: *r*^2^ = *0.017, p* = *0.468*	Moberly et al., 2018c	34
		Blockwise multiple linear regression analysis	(–) Adding Stroop to the model to predict meaningful SQ: β = –0.259, *p* = 0.008, *(ns)* anomalous*:*β = *0.163, p* = *0.273*, df = 32	Moberly and Reed, 2019	41
		ANOVA, spearmans rank-order correlations divided in 3 groups	(–) Harvard sentences high-smrt: rho = –0.047, *p* = 0.03, *(ns)* Harvard*:* low-smrt: *rho* = *0.27, p* = *0.16*, intermediate-smrt: *rho* = −*0.35, p* = *0.09*, PRESTO: low-smrt: *rho* = *0.40, p* = *0.06*, intermediate-smrt*: rho* = −*0.40, p* = *0.07*, high-smrt*: rho* = −*0.35, p* = *0.08*	Moberly et al., 2021	51
	SN	Pearson correlations	(–) Incongruent: dyslexia:*r* = –0.41, *p* < 0.05, HINT-C: *r* = –0.43, *p* < 0.05 *(ns)* congruent: dyslexia: *r* = −*0.28*, HINT-C: *r* = −*0.36*	Moberly et al., 2016b	30
			(–) *r* = –0.43, *p* < 0.05	Moberly et al., 2017b	30
	WQ SQ	Patial least squares regression	*(ns)*	Skidmore et al., 2020	40
	SQ + N	Pearson correlations	(+) Incongruent: SQ: *r* = –0.484, *p* = 0.042 *(ns)* incongruent: SN: *r* = −*0.412, p* = *0.09*	Zhan et al., 2020 *	19
Flanker task	WQ	Rank ANOVA, DFA	(+) Cohen’s *d* = 0.58, *p* = 0.037, discriminant *r* = 0.21	Völter et al., 2021	34
	SN	Generalised linear mixed-effects regression analysis	*(ns)*	Tinnemore et al., 2020	10
**Flexibility**					
TMT-B → see attention Table 5					
NIH DCCS test	SQ	Generalized linear mixed-effects regression analysis	(+) Higher than average scores associated with speech recognition *p* = 0.006, estimate = 0.35	Tinnemore et al., 2020	10

Overview of included papers using general cognitive measures and assessing cognitive inhibition and flexibility. The task, speech perception outcome measure (W, words; S, sentence; Q, quiet; N, noise), statistical analysis, key finding [(+), positive significant result; (–), negative significant result; *(ns)*, *non-significant result*), reference and sample size (N) are reported. DCCS, dimensional change card sort; DFA, discriminant factor analysis, MoCA, Montreal Cognitive Assessment; NIH, National Institutes of Health, TMT-B, Trail Making Task version B; *Cognitive measure preoperatively. A more detailed version can be found in Supplementary Table 4.

Eleven included studies used the Stroop task, where response time was measured, and a lower value represented better performance on the task. Four of four studies show that performance on this task, both preoperatively and postoperatively, did not significantly correlate with or predict word perception in quiet (Moberly et al., 2018c; Skidmore et al., 2020; Zhan et al., 2020). Two exceptions exist which showed a significant negative correlation with word perception in quiet: (1) in a group having high auditory sensitivity (rho = –0.49, *p* = 0.02) (Moberly et al., 2021) (2) with word perception after ten trials of a task, when the listener has adapted to the speech (*r* = –0.50, *p* = 0.044) (Tamati and Moberly, 2021). Three of seven studies showed a significant negative relation with sentence perception tests in quiet (adding Stroop to the model to predict meaningful SQ *p* = 0.008, β = –0.259; *r* = –0.43, *p* < 0.05; *r* = –0.41, *p* < 0.05) (Moberly et al., 2016b, 2017b, 2018a,c; Moberly and Reed, 2019; Tamati et al., 2020; Zhan et al., 2020). Additionally, a significant negative correlation was found between Stroop task performance and sentence perception in quiet, for a group having high auditory sensitivity (rho = –0.047, *p* = 0.03) (Moberly et al., 2021). Furthermore, preoperative Stroop was significantly negatively correlated with postoperative sentence perception in quiet and noise (AzBio: Q *r* = –0.484, *p* = 0.042, N *r* = –0.412, *p* = 0.09, Harvard: standard *r* = –0.321, *p* = 0.193, anomalous *r* = –0.319, *p* = 0.197, PRESTO *r* = –0.301, *p* = 0.224) (Zhan et al., 2020) (Note that many of these studies including the Stroop task were performed in the same lab, some using the same participants, which might hamper generalizability).

Additionally, two included studies used the Flanker task. They showed that performance on this task significantly differed between better and poorer performers on a word task in quiet (Cohen’s d = 0.58, *p* = 0.037) (Völter et al., 2021). However, the performance did not significantly predict performance for sentence perception in quiet (Tinnemore et al., 2020).

##### 3.2.3.4. Flexibility

Flexibility, often referred to as executive control, encompasses functions related to planning and task switching. It is mostly found to be supported by the frontal lobe (Gilbert and Burgess, 2007), which seems to be more activated in better performers. The TMT-B is not only used to measure attention, but also executive control or flexibility. Additionally, the “NIH Toolbox Dimensional Change Card Sort Test” (DCCS) is used (see Table 9 for an overview). As discussed before, the TMT-B score showed inconsistent correlations with word or sentence perception in quiet (Hua et al., 2017; Völter et al., 2021; Zucca et al., 2022). The other task was only applied in one paper. Performance on the DCCS, which asks participants to match cards with a target card based on different properties, was found to be significant in a general linear model with sentences in quiet (*p* = 0.006) (Tinnemore et al., 2020).

#### 3.2.4. Social cognition

None of the included studies contained measures of social cognition related to speech perception outcomes.

#### 3.2.5. Learning and memory

Memory is the ability to store learned information and retrieve it over time. In the brain activation section, it was observed that the temporal and frontal cortex are recruited in better performers. Those areas are mainly involved in memory formation, and together with brain plasticity indicate learning (Breedlove and Watson, 2013). These skills can be measured in different ways. In the included papers this is done using recall tasks (Moberly et al., 2017a; Hillyer et al., 2019; Völter et al., 2021), learning tasks (Zucca et al., 2022), or both (Holden et al., 2013; Pisoni et al., 2018; Kessler et al., 2020; Skidmore et al., 2020; Tamati et al., 2020; Ray et al., 2022), mostly in the verbal domain (see Table 10a for an overview).

**TABLE 10:** Overview of included papers studying learning and memory and perceptual motor function.

Cognitive measure	Speech measure	Statistical analysis	Results	References	*N*
**(a) Learning and memory**					
**Recognition memory**					
WJ-IV picture recognition test	SQ	Pearson correlations, controlling for age	*(ns)*	Hillyer et al., 2019	21
**Verbal learning and recall**					
Auditory word recall (and delayed recall) task	WQ	Mann-Whitney U test, regression analysis	*(ns)* immediate*: p* = *0.343; r^2^* = *0.049*, β = *0.222, p* = *0.346* differite*: p* = *0.455: r^2^* = *0.110*, β = *0.331, p* = *0.154*	Zucca et al., 2022 *	15
		Rank ANOVA, DFA	(+) Delayed recall, Cohen’s d = 0.88, p = 0.04 discriminant *r* = 0.29 *(ns)* recall: *Cohen’s d* = *0.6, p* = *0.12*	Völter et al., 2021	34
	WQ + N SQ + N	Correlation analysis	*(ns)*	Moberly et al., 2017a	30
CERAD-plus test battery	WQ SQ + N	Independent *T*-test	*(ns)*	Kessler et al., 2020	21
CVLT –II	WQ	Non-parametric correlation and principal component measures. Divided in six groups	*(ns)* when corrected for age	Holden et al., 2013 *	92
		Correlational and regression analysis	(+) List B: *r* = 0.47, *p* < 0.05, added value to model	Pisoni et al., 2018	25
	SQ	Correlational and regression analysis	(+) List B: Harvard words *r* = 0.48, *p* < 0.05, sentences: *r* = 0.56, *p* < 0.05, PRESTO words: *r* = 0.52, *p* < 0.05, sentences *r* = 0.52, *p* < 0.05, List A trial five Harvard words: *r* = 0.46, *p* < 0.05 *(ns)* rest	Pisoni et al., 2018	25
		Pearson correlations, DFA	List B: matrix coefficient = 0.16, rank 5, discriminability: matrix coefficient = 0.12, rank 6, T1/T5: matrix coefficient = –0.04, rank 9, df = 10	Tamati et al., 2020	21
	WQ SQ	Patial least squares regression	*(ns)*	Skidmore et al., 2020	40
	SQ + N	Partial least squares regression with VIP (robust approach)	(+) Most important variables: short-delay cued recall, semantic clustering, subjective clustering, primacy recall and recall consistency (VIP more than one), refittet model 35.8% explained, Each variable explained more than 50% of the variance	Ray et al., 2022	32
**(b) Perceptual-motor function**					
WJ-IV visualization parts A and B	SQ	Pearson correlations, controlling for age	*(ns)*	Hillyer et al., 2019	21
Corsi block tapping test	WQ	Mann-Whitney U test, regression analysis	*(ns)* backward: *p* = *0.220; r^2^* = *0.103*, β = *0.284, p* = *0.156*, forward: *p* = *0.588;, r^2^* = *0.081*, β = *0.321, p* = *0.212*	Zucca et al., 2022 *	15
Block rotation task	SQ	Pearson correlations, controlling for age	*(ns)*	Hillyer et al., 2019	21

Overview of included papers assessing cognitive a) learning and memory and b) perceptual motor function. The task, speech perception outcome measure (W, words; S, sentence; Q, quiet; N, noise), statistical analysis; key finding [(+), positive significant result; (–), negative significant result; *(ns)*, *non-significant result*], reference and sample size (N) are reported. CVLT, California Verbal learning test; DFA, discriminant factor analysis, MoCA, Montreal Cognitive Assessment; WJ-IV, Woodcock-Johnson IV; *Cognitive measure preoperatively. A more detailed version can be found in Supplementary Table 4.

In five papers recall tasks were used (Moberly et al., 2017a; Hillyer et al., 2019; Kessler et al., 2020; Völter et al., 2021; Zucca et al., 2022). Performance scores did not significantly correlate with, predict or dissociate better and poorer performers on sentence perception in quiet or noise. However, there was a significant difference in the “ALAcog delay recall score” between CI users that had higher and lower performance on a word perception task (Cohen’s d = 0.88, *p* = 0.04) (Völter et al., 2021) as opposed to Zucca et al. (2022) who did not find a significant result when it was measured preoperatively (*p* = 0.343, *p* = 0.445).

Furthermore, the CVLT test battery was used in five included papers to assess verbal learning and recall (Holden et al., 2013; Pisoni et al., 2018; Skidmore et al., 2020; Tamati et al., 2020; Ray et al., 2022). The CVLT includes various short- and long-term recall tasks, and calculates several scores reflecting word recall strategies. In two out of five papers, scores on this task did not significantly correlate with word and sentence perception in quiet or noise when administered pre- or post-implantation (Holden et al., 2013; Pisoni et al., 2018; Skidmore et al., 2020). If a relationship was found, it was always a subtest of the battery. In these studies, (1) recall on list B, was positively correlated with words and sentence perception in quiet (WQ: *r* = 0.47 SQ: *r* = 0.56, *r* = 0.52, *P* < 0.05) (Pisoni et al., 2018), (2) sub-scores short delay cued recall, semantic clustering, subjective clustering, primacy recall and recall consistency were important predictors of sentence perception in quiet and noise (Ray et al., 2022) and (3) list B and Y/N discriminability could discriminate very little between better and poorer performers on a sentences in quiet task, and T1/T4 not at all (Tamati et al., 2020).

#### 3.2.6. Perceptual-motor function

Perceptual motor skills allow individuals to interact with the environment by combining the use of senses and motor skills. These skills are involved in many of the tasks discussed above. Only two papers used tasks that explicitly measure these skills (see Table 10b for an overview) (Hillyer et al., 2019; Zucca et al., 2022). Tasks used to measure this skill are the “WJ-IV visualization parts A and B,” the “corsi block tapping test” and the “block rotation task.” These tasks were only applied in one paper each and did not show any significant relation with speech perception performance (e.g., *r* = 0.081–0.103, *p* = 0.156–0.588) (Hillyer et al., 2019; Zucca et al., 2022).

### 3.3. Language

Many of the cognitive tasks mentioned above already include verbal ability assessments, for example verbal working memory, learning and recall. Although these different cognitive functions do not seem to correlate consistently with speech perception outcomes, it is valuable to explore what the included literature says about language skills in CI users and the relationship with speech perception outcomes. An overview of the studies including language assessments can be found in Table 4.

#### 3.3.1. Object naming and word finding (vocabulary)

Vocabulary is the language user’s knowledge of words. In the included papers vocabulary is assessed by picture naming tasks (Collison et al., 2004; Holden et al., 2013; Kaandorp et al., 2015; Völter et al., 2021), choosing synonym tasks (Collison et al., 2004; Kaandorp et al., 2015, 2017), discriminating real words from pseudowords (Kaandorp et al., 2017; Moberly et al., 2017a; Kessler et al., 2020; Tamati et al., 2020; Völter et al., 2021), by reporting the degree of familiarity with words (Pisoni et al., 2018; Skidmore et al., 2020; Bosen et al., 2021; Tamati et al., 2021), or describing the similarity or difference between two words (Holden et al., 2013) (see Table 11 for an overview). In three out of ten papers there was an indication of a relationship between vocabulary and speech perception outcomes. First, there was a difference in score on the Rapid Automatic Naming (RAN) task and a lexical decision task between a group of poorer and better performing CI users on a word perception task in quiet, where better performers had significantly higher RAN task scores (Cohen’s d = –0.82 to –1.34, *p* = 0.0021–0.031) and non-word discrimination task scores (Cohen’s d = –1.27, *p* = 0.0021) (Völter et al., 2021). Secondly, performance on a lexical decision task was found to be a significant predictor of the average score of both word and sentence perception in noise (*r* = 0.45, *p* = 0.047) (Kaandorp et al., 2017). Third, there was a significant correlation between performance on a word recognition test and sentences in quiet (*r* = 0.45, *p* < 0.05) (Pisoni et al., 2018).

**TABLE 11:** Overview of included papers studying vocabulary and verbal fluency.

Cognitive measure	Speech measure	Statistical analysis	Results	References	*N*
**Object naming and word finding (vocabulary)**					
Picture naming task	WQ	Pearson correlations	*(ns) r* = *0.501*	Collison et al., 2004	15
		Non-parametric correlation and PCA.	*(ns)* when corrected for age	Holden et al., 2013 *	92
		Rank ANOVA, DFA	(–) objects Cohen’s *d* = –1.28, *p* = 0.0026, Colors: Cohen’s *d* = –0.82, *p* = 0.031, letters: Cohen’s *d* = –1.25, *p* = 0.0026, numbers: Cohen’s *d* = –1.34, *p* = 0.0038, discriminant: *r* = 0.56	Völter et al., 2021	34
	WQ + N SQ	Regression modeling	*(ns)*	Kaandorp et al., 2015	24
Choosing a synonym task	WQ	Pearson correlations	*(ns)* Part of VCS see below	Collison et al., 2004	15
	WQ SQ + N	Regression modeling	*(ns)*	Kaandorp et al., 2015	24
		Correlation analysis	*(ns)* WQ: *r* = −*0.19*, WN: *r* = −*0.19*, SN: *r* = −*0.33*, SRTdiff: *r* = −*0.27*	Kaandorp et al., 2017	20
Word naming test	WQ SQ + N	Correlation analysis	*(ns)* WQ: *r* = −*0.02*, WN: *r* = −*0.03*, SN: *r* = *0.12*, SRTdiff: *r* = *0.18*	Kaandorp et al., 2017	20
Word vs non-word discrimination task	WQ	Correlation analysis	*(ns)*, df = 11	Finke et al., 2016	13
		Rank ANOVA, DFA	(–/+) Sensitivity Cohen’s *d* = –1.27, *p* = 0.0021 Discriminant *r* = 0.54, Response time existing words Cohen’s d = 0.85, *p* = 0.017,	Völter et al., 2021	34
	SQ	Correlation analysis	*(ns)*, df = 11	Finke et al., 2016	13
	WQ SQ + N	Correlation analysis, regression analysis	(+) SRTdiff: *r* = 0.45, *p* = 0.047, explained additional 36% in model *(ns)* WQ: *r* = −*0.25*, WN: *r* = *0.07*, SN: *r* = *038*	Kaandorp et al., 2017	20
	WQ SQ + N	Correlation analysis	*(ns)*, df = 28	Moberly et al., 2017a	30
WordFam-150 test	WQ	Correlation analysis	*(ns)*	Pisoni et al., 2018	25
	SQ	Correlation analysis	(+) PRESTO sentences *r* = 0.45, *p* < 0.05 *(ns)* Harvard words and sentences and PRESTO words	Pisoni et al., 2018	25
		Pearson correlation	*(ns) r* = *0.21, p* = *0.39*, corrected for age *r* = *0.16, p* = *0.50*	Bosen et al., 2021	20
	WQ SQ	Partial least squares regression	*(ns)*	Skidmore et al., 2020	40
WJ-III verbal comprehension section (VCS)	WQ	Pearson correlations	*(ns) r* = *0.286*	Collison et al., 2004	15
WAIS-III similarities test	WQ	Non-parametric correlation and PCA	*(ns)* when corrected for age	Holden et al., 2013 *	92
**Verbal fluency**					
Verbal Fluency Task	WQ	Correlation analysis	*(ns) r(11)* = *0.536, p* = *0.059*	Finke et al., 2016	13
		Rank ANOVA	(+) Cohen’s *d* = 0.80, *p* = 0.025	Völter et al., 2021	34
		Mann-Whitney U test, regression analysis	*(ns)* Phonemic: *p* = *0.218; r^2^* = *0.002*, β = *0.049, p* = *0.834* semantic*: p* = *0.052; r^2^* = *0.165*, β = *0.407, p* = *0.067*	Zucca et al., 2022 *	15
	SQ	Independent *T*-test	*(ns)*	Kessler et al., 2020	21
		Correlation analysis	*(ns) r(11)* = *0.518, p* = *0.061*	Finke et al., 2016	13

Overview of included papers assessing vocabulary and verbal fluency. The task, speech perception outcome measure (W, words, S, sentence; Q, quiet; N, noise), statistical analysis, key finding [(+), positive significant result; (–), negative significant result; *(ns)*, *non-significant result*), reference and sample size (N) are reported. DFA, discriminant factor analysis; PCA, principal component analysis; *Cognitive measure preoperatively. A more detailed version can be found in Supplementary Table 4.

#### 3.3.2. Verbal fluency

Verbal fluency is the readiness in which words are accessed and produced from one’s own long-term lexical knowledge. Four of the papers address verbal fluency (Finke et al., 2016; Kessler et al., 2020; Völter et al., 2021; Zucca et al., 2022) (see Table 11 for an overview). Performance on the verbal fluency tasks was assessed in four papers. In three out of four no significant relationship with word and sentence perception in quiet was found (Finke et al., 2016; Kessler et al., 2020; Zucca et al., 2022). Performance did significantly differ between better and poorer performers on a word perception task in quiet (Cohen’s d = 0.8, *p* = 0.025) (Völter et al., 2021).

#### 3.3.3. Speed of lexical and phonological access

Speed of lexical and phonological access represents how fast written text is generated into phonemes or meaningful speech. Speed of lexical and phonological access is assessed in ten of the included papers. Speed reading tasks of real and non-words and sentences, such as the “Test Of Word Reading Efficiency” (TOWRE) and the “Wide Range Achievement Test” (WRAT), are mostly used for this (Hay-McCutcheon et al., 2005; Moberly et al., 2018a,c, 2021; Pisoni et al., 2018; Moberly and Reed, 2019; Skidmore et al., 2020; Tamati et al., 2020, 2021) (see Table 12 for an overview).

**TABLE 12:** Overview of included papers studying speed of lexical and phonological access and degraded receptive language.

Cognitive measure	Speech measure	Statistical analysis	Results	References	*N*
**Speed of lexical and phonological access**					
TOWRE	WQ	Partial correlation analysis, controlled for age	(+) Words: *r* = 0.47, *p* < 0.01	Moberly et al., 2018a	42
		Correlational and regression analysis	(+) TOWRE words: *r* = 0.55, *p* < 0.05, TOWRE non-words: *r* = 0.41, *p* < 0.05.	Pisoni et al., 2018	25
		Linear regression analysis	(+) Words: *r*^2^ = 0.312, β = 0.558, *p* = 0.001, non-words: *r*^2^ = 0.173, β = 0.416, *p* = 0.014	Moberly et al., 2018c	34
		ANOVA, spearmans rank-order correlations divided in 3 groups	(+) Intermediate-smrt: rho = 0.48, *p* = 0.03, high-smrt: rho = 0.42, *p* = 0.05 *(ns)* low-smrt: *rho* = −*0.08, p* = *0.39*	Moberly et al., 2021	51
	SQ	Pearson correlations	(+) Real words and Harvard standard: *r* = 0.36, *p* = 0.015, real words and Harvard anomalous: *r* = 0.42, *p* = 0.004, total and Harvard standard: *r* = 0.35, *p* = 0.018, total and Harvard anomalous: *r* = 0.36, *p* = 0.016, real words and PRESTO words: *r* = 0.40, *p* = 0.006, total and PRESTO words: *r* = 0.47, *p* = 0.014	Tamati et al., 2021	48
		Partial correlation analysis, controlled for age	(+) Words and PRESTO words: *r* = 0.47, *p* < 0.01, words and PRESTO: *r* = 0.54, *p* < 0.01, non-words: *r* = 0.40, *p* < 0.05, words and Harvard words: words *r* = 0.37, *p* < 0.05, words and Harvard sentences: words *r* = 0.57, *p* < 0.05, non-words *r* = 0.45, *p* < 0.01	Moberly et al., 2018a	42
		Pearson correlations, DFA	Words: Matrix coefficient = 0.25, rank 3, Non-words: Matrix coefficient = 0.22, rank 4, df = 10	Tamati et al., 2020	21
		Correlational and regression analysis	(+) TOWRE words: Harvard sentences *r* = 0.47, *p* < 0.05, PRESTO words *r* = 0.41, *p* < 0.05, sentences *r* = 0.41, *p* < 0.05, TOWRE non-words: Harvard words r = 0.49, *p* < 0.05, sentences *r* = 0.48, *p* < 0.05, PRESTO sentences *r* = 0.48, *p* < 0.05 *(ns)* rest	Pisoni et al., 2018	25
		Linear regression analysis	(+) Words and Harvard words *r*^2^ = 0.175, β = 0.418, *p* = 0.015 words and PRESTO words *r*^2^ = 0.187, β = 0.435, *p* = 0.011	Moberly et al., 2018c	34
		Blockwise multiple linear regression analysis	(+) Adding TOWRE words to predict anomalous sentences β = 0.391, *p* = 0.010 *(ns)* meaningful: β = −*0.81, p* = *0.414*, df = 32	Moberly and Reed, 2019	41
		ANOVA, Spearmans rank-order correlations Divided in 3 groups	(ns) Harvard: low-smrt: rho = –0.23, *p* = 0.20, intermediate-smrt: rho = 0.35, *p* = 0.09, High-smrt: rho = –0.23, *p* = 0.20 PRESTO: low-smrt: rho = –0.31, p = 0.12, intermediate-smrt: rho = 0.30, *p* = 0.13, high-smrt: rho = 0.37, *p* = 0.07	Moberly et al., 2021	51
	WQ SQ	Partial least squares regression	*(ns)*	Skidmore et al., 2020	40
WRAT word reading	WQ SQ	Partial least squares regression	*(ns)*	Skidmore et al., 2020	40
Speechreading sentences	WQ SQ + N	Pearson correlations	(–) Young group: *r* = –0.872, *p* = 0.002 *(ns)* Older group: *r* = *0.0562, p* = *0.189*	Hay-McCutcheon et al., 2005 *	34
LEMO subtest of internal homophonic word reading	WQ	Rank ANOVA	(+) Cohen’s *d* = –1.23, *p* = 0.0039	Völter et al., 2021	34
Audiovisual non-word repetition task	WQ + N SQ + N	Correlation analysis	*(ns)*, df = 28	Moberly et al., 2017a	30
**Degraded receptive language**					
TRT	WQ	Rank ANOVA	(–) Periodic bars: Cohen’s *d* = –1.57, *p* = 0.00002, Floating bars: Cohen’s *d* = –1.25, *p* = 0.00021, Random dots: Cohen’s *d* = –0.94, *p* = 0.0021	Völter et al., 2021	34
	SN	Correlation analysis	SN unmmodulated: (–) TRT random dots *r* = –0.23 *P* = 0.036 *r*^2^ = 0.05, TRT random bars *r* = –0.27, *P* = 0.012, *r*^2^ = 0.07, SN modulated: TRT random dots *r* = –0.29, *P* = 0.007, *r*^2^ = 0.09, TRT random bars *r* = –0.28, *P* = 0.009, *r*^2^ = 0.08 SN fixed: (+) TRT random noise *r* = 0.26, *P* = 0.026, *r*^2^ = 0.07	Haumann et al., 2012 *	97
	WQ SQ	Correlation analysis	*(ns) WQ: r = –0.22, WN: r = –0.19, SN: r = –0.33, SRTdiff: r = –0.27*	Kaandorp et al., 2017	20
Fragmented sentences test	WQ	Linear regression analysis	(+) *r*^2^ = 0.157, β = 0.396, *p* < 0.001	Moberly et al., 2018c	34
	SQ	Linear regression analysis	*(ns)* Harvard: *r^2^* = *0.055*,β = *0.234, p* = *0.109* PRESTO: *r^2^* = *0.11*,β = *0.334, p* = *0.058*	Moberly et al., 2018c	34

Overview of included papers assessing speed of lexical and phonological access and degraded receptive language. The task, speech perception outcome measure (W, words; S, sentence; Q, quiet; N, noise), statistical analysis (including a report of a power analysis (y), yes; (n), no; df, degrees of freedom), key finding [(+), positive significant result, (–), negative significant result; *(ns)*, *non-significant result*), reference and sample size (N) are reported. DFA, discriminant factor analysis, TOWRE, Test of word reading efficiency; TRT, text reception threshold; *Cognitive measure preoperatively. A more detailed version can be found in Supplementary Table 4.

In eight papers, TOWRE was used. Participants had to read aloud as many words or non-words in a list in 45 seconds for this task. Some studies included the same participants, therefore, in three out of five study populations there were significant positive correlations between performance on the TOWRE word and non-word scores and word and sentence perception in quiet (*r* = 0.41–0.49, *p* < 0.05; adding model to predict anomalous sentences *p* = 0.010, df = 32) (Moberly et al., 2018a,c, 2021; Pisoni et al., 2018; Moberly and Reed, 2019; Skidmore et al., 2020; Tamati et al., 2021). More specifically, when performing a regression analysis, Moberly et al. (2018a) found that only TOWRE word and non-word score was related to word and sentence perception in quiet (WQ: *r*^2^ = 0.312, *p* = 0.001, non-words: *r*^2^ = 0.173, *p* = 0.014, SQ: Harvard: *r*^2^ = 0.175, *p* = 0.015, PRESTO: *r*^2^ = 0.187, *p* = 0.011). Lastly, Moberly et al. (2021), found a significant positive correlation with word perception in quiet for participants with an intermediate and high degree of auditory sensitivity (intermediate: rho = 0.48, *p* = 0.03, high: rho = 0.42, *p* = 0.05).

The remaining tasks used to study speed of lexical and phonological access: the WRAT, “preoperative speechreading of sentences,” “Lexical Model Oriented (LEMO) subtest of internal homophonic word reading” and non-word repetition task were only applied in one study each. The results varied and showed significant positive (Cohen’s d = –1.23, *p* = 0.0039) (Völter et al., 2021), negative (*r* = –0.872, *p* = 0.002) (Hay-McCutcheon et al., 2005) and non-significant relationships (Moberly et al., 2017a; Skidmore et al., 2020).

#### 3.3.4. Degraded receptive language

Degraded receptive language is captured with the Text Reception Threshold (TRT) task. This is a visual analog of the Speech Reception Threshold (SRT) task, where sentences are masked using different visual patterns. The participant needs to try and read the sentences and is scored based on the degree of masking at which they are able to repeat 50% of the words correctly. This task was used in three included studies (Haumann et al., 2012; Kaandorp et al., 2017; Völter et al., 2021) (see Table 12 for an overview). In one of the three papers the TRT was measured preoperatively (Haumann et al., 2012). Results of these papers assessing performance the TRT and a very similar fragmented sentences test task were highly variable: both non-significant results (Kaandorp et al., 2017), significantly positive (Cohen’s d = –0.94 to 1.57, *p* = 0.0021–0.0002; *r*^2^ = 0.157, *p* < 0.001) (Moberly et al., 2018c; Völter et al., 2021) and negative relations (SN modulated: TRT random dots *r* = –0.23, *r*^2^ = 0.05, *p* = 0.036, TRT random bars *r* = –0.27, *r*^2^ = 0.07, *p* = 0.012, SN modulated: TRT random dots *r* = –0.29, *r*^2^ = 0.09, *p* = 0.007, TRT random bars *r* = –0.28, *r*^2^ = 0.08, *p* = 0.009) (Haumann et al., 2012) were found.

## 4. Discussion

This scoping review aimed to provide a comprehensive overview of the current literature on the relationship between both neurocognitive factors and brain activation patterns, with speech perception outcomes in postlingually deafened adult CI users. Fifty-four papers were included and divided into three categories: (1) literature discussing different brain activation patterns in better and poorer CI performers, (2) literature relating performance on cognitive tasks to speech perception outcomes, and (3) literature relating performance on cognitive language tasks to speech perception outcomes.

### 4.1. Brain areas recruited in better CI performers

Overall, literature studying brain activation patterns in CI listeners demonstrated that better performers in quiet or noise showed increased activation in the left frontal areas and temporal cortex when passively listening to noise, speech and non-speech stimuli, and actively to semantically correct and incorrect sentences. The frontal lobe is thought to be involved in several speech-related functions, such as semantic generation, decision making and short-term memory (Mortensen et al., 2006; Strelnikov et al., 2013), while the temporal cortex is the main hub for auditory and speech processing. However, activity in the premotor cortex and parietal cortex showed less consistent links with performance. These areas are involved in planning movement and spatial attention, respectively (Breedlove and Watson, 2013).

Moreover, cross-modal activation in the visual occipital cortex during speech perception was seen in better performers. Conversely, visual stimuli activating the auditory temporal cortex was observed in poorer performers. This suggests that learning auditory speech perception with a CI is facilitated by visual cues, yet visual cues should not be the main input for the auditory cortex. In practice, provided the beneficial activation in the visual cortex is higher than the activation induced by visual stimuli in the auditory cortex, speech perception in quiet is more successful. This occurrence of cross-modal activation might be related to duration of deafness and plasticity postimplantation (Buckley and Tobey, 2010; Sandmann et al., 2012; Song et al., 2015a; Chen et al., 2016, 2017; Han et al., 2019).

Subsequently, Lazard et al. (2010, 2011) and Lazard and Giraud (2017) investigated both the involvement of the visual cortex during listening and cross-modal activation in the auditory cortex in CI candidates. They found that performance after implantation depended on activation of either the dorsal route or the ventral route during sound imagery tasks, indicating the use of phonological and speech sound properties, or the use of lexico-semantic properties, respectively. This confirmed the importance of maintaining both the temporal and occipital cortex for normal sound or language processing [such as phonological processing and the integration of visual cues (visemes) with phonological properties] even if the input is not auditory. It seems that if only fast semantic- or lexical-based strategies become the default during the time of deafness, it is hard to return to incorporating original slow speech sound-based strategies once implanted, which contributes to poorer performance. Future research may provide further insights into what causes CI listeners to use different speech perception strategies engaging different brain areas, leading to better or poorer outcomes.

### 4.2. Cognitive factors related to speech perception outcomes

Several observations were made from the literature studying cognitive performance in CI listeners and its association with speech perception outcomes.

First, non-verbal intelligence, assessed using the Ravens Matrices task, was positively related to word or sentence perception in quiet in most studies (9 out of 13) (Moberly et al., 2017c, 2018c, 2021; Mattingly et al., 2018; Pisoni et al., 2018; Moberly and Reed, 2019; O’Neill et al., 2019; Skidmore et al., 2020; Zhan et al., 2020; Tamati and Moberly, 2021; Tamati et al., 2021). The Ravens task is thought to, amongst other things, involve the ability of inducing abstract relations as well as working memory (Carpenter et al., 1990). Since it is suggested that several basic cognitive functions are involved in performing the Ravens task, it is unclear whether one of these cognitive functions underlie the observed relationship with speech perception performance (Mattingly et al., 2018). Studies that used other tasks to measure the same domain failed to provide additional evidence for the association of the cognitive subdomain non-verbal intelligence with speech perception outcomes (Collison et al., 2004; Holden et al., 2013; Moberly et al., 2016b).

Second, performance on auditory and visual working memory tasks was unrelated to speech perception outcomes in most studies (11 of 15) (Holden et al., 2013; Moberly et al., 2016b, 2017a,b,c, 2018c, 2021; Moberly and Reed, 2019; Skidmore et al., 2020; Tamati et al., 2020; Zhan et al., 2020; Bosen et al., 2021; Tamati and Moberly, 2021; Luo et al., 2022). When both modalities were combined in the working memory task, or more verbal aspects were added, significant correlations with word and sentence perception, both in quiet and noise, appeared to be more prevalent. However, there were only a limited number of studies assessing these types of working memory and thus more data is required to draw any conclusions. Interestingly, Luo et al. (2022), performed a more extensive verbal working memory task, including a cued and uncued working memory condition. When corrected for temporal and spectral resolution, only a significant positive correlation remained between performance in the uncued condition and word perception in noise, but not sentence perception in noise. This suggests that while top-down information is less available, as in the uncued condition of the working memory task (compared to the cued condition), similar working memory processes are at play as during word perception in noise (compared to sentence perception in noise). More research is needed to confirm whether a specific type of working memory is involved in particular speech perception tasks, in the same way that working memory is thought to be modality-specific (Park and Jon, 2018). Working memory processes would enable the listener to retain relevant information while listening to speech [as suggested by the ease of language understanding model (ELU) (Rönnberg et al., 2013)].

Third, cognitive inhibition was generally unrelated to word perception in quiet; a negative relationship was only observed in people with a high degree of auditory sensitivity or after adaptation to speech (Moberly et al., 2021; Tamati and Moberly, 2021). The relationship with sentence perception in quiet was less clear and in several papers negative relationships were observed (3 of 7) (Moberly et al., 2016b, 2017b, 2018a,c; Moberly and Reed, 2019; Tamati et al., 2020; Zhan et al., 2020). This possibly indicates that inhibiting information is engaged more in sentence perception compared to word perception. In theory, sentences contain more information than single words, and interfering information needs to be suppressed while items are retained in working memory (Rönnberg, 2003). It should be noted, however, that since most of these studies were performed in the same lab within the same participant sample, the results should be considered carefully. Additionally, only one main task, the Stroop task, was used to assess cognitive inhibition. It is possible that by implementing the Flanker task more often, different results might be observed, as both tasks measure different facets of inhibitory control (Knight and Heinrich, 2017).

Fourth, performance on standard recall tasks assessing the cognitive domain learning and memory in general did not to correlate with speech perception performance (Holden et al., 2013; Moberly et al., 2017a; Pisoni et al., 2018; Hillyer et al., 2019; Kessler et al., 2020; Skidmore et al., 2020; Tamati et al., 2020; Völter et al., 2021; Ray et al., 2022; Zucca et al., 2022). This is contrary to expectations, as the relevance of these skills is often emphasized in speech perception models (Rönnberg et al., 2013). One explanation for this discrepancy could be that these tasks are not reflective of the use of memory and learning for everyday speech perception. The fact that scores on subtests of the CVLT showed significant positive correlations with sentence perception in quiet and noise (Pisoni et al., 2018; Ray et al., 2022), might already give an example of a test or scoring method more representative of memory and learning skills involved in speech perception. Compared to simple word and picture recall tasks, this test calculates specific scores on, for example, semantic clustering or recall consistency.

Although these studies provide some indications, for many of the cognitive functions there is no or insufficient data to make any inferences. Often, one task is applied only within a single study or results are inconsistent. This is true for social cognition, the general cognitive measures, attention, processing speed, flexibility, audio-visual and verbal working memory, and perceptual-motor function. Moreover, most studies do not report any power analysis, which further increases the unreliability of results should the power be insufficient. One example where a greater sample size seemed to lead to clearer outcomes was for the general cognitive measures. These did not predict word perception in quiet with a sample size of 15 (Zucca et al., 2022). However, research including a larger sample size (df = 32) (Wazen et al., 2020) did show the effectiveness of a quick cognitive assessment for predicting sentence perception in quiet and noise preoperatively. Furthermore, it might be beneficial to consider social cognition, the only cognitive domain currently not covered in the literature. This domain might be of value to CI listeners, as better social skills might lead to more social exposure and therefore more listening practice (Knickerbocker et al., 2021). Therefore, it seems that it would be worthwhile to include this cognitive domain in future research.

### 4.3. Language skills related to speech perception outcomes

Language is the most interesting cognitive domain in the context of the current paper, as speech perception is part of this domain. According to the outcomes of the included papers, vocabulary was not associated with speech perception performance (7 out of 10 papers) (Collison et al., 2004; Kaandorp et al., 2015, 2017; Shafiro et al., 2016; Moberly et al., 2017a; Kessler et al., 2020; Tamati et al., 2020; Völter et al., 2021). For both verbal fluency and degraded language perception, only a few papers were included, which did not allow to make any inferences about these cognitive skills (Haumann et al., 2012; Finke et al., 2016; Kaandorp et al., 2017; Moberly et al., 2018c; Kessler et al., 2020; Völter et al., 2021; Zucca et al., 2022). The last skill, speed of lexical and phonological access, was often shown to be significantly positively correlated with word and sentence perception when assessed using TOWRE (3 of 5 study populations) (Moberly et al., 2018a,c, 2021; Pisoni et al., 2018; Moberly and Reed, 2019; Skidmore et al., 2020; Tamati et al., 2021). Overall, it seems that in adult CI users, rather than lexical knowledge, the ability to form words quickly and efficiently from phonemes or written text is crucial for speech perception outcomes (even if bottom-up information is incomplete).

### 4.4. Suggestions for future research

This literature overview points toward some cognitive factors predicting or failing to predict speech perception performance. Unfortunately, a considerable number of reviewed studies showed inconsistent results. As more studies are needed to validate the conclusions above, possible reasons for inconsistency and suggestions to improve future studies are provided:

First, tasks capture scores in different manners when evaluating a cognitive skill. For example, a significant positive correlation was found when measuring response time per trial in an attention task, but not when measuring accuracy within a prespecified time frame (Moberly et al., 2016b; Hillyer et al., 2019; Völter et al., 2021). Similarly, some tasks are more engaging compared to others which aim to assess the same cognitive skills. This might lead to differences in validity between these tests. For example, of two measures assessing attention, the TMT-B requires more use of semantic knowledge compared to pattern matching. Future studies might consider using different measures assessing the same cognitive skills, or one task under different conditions, to determine what feature of the task is relevant for assessing a certain cognitive skill in relation to speech perception outcomes.

Second, the time of assessment might influence results. Significant positive correlations of performance on the TMT-A with speech perception were found when measured preoperatively (Zucca et al., 2022), but similar measures performed postoperatively did not show such a relationship with speech perception outcomes (Hua et al., 2017; Völter et al., 2021). Performing the same cognitive test before and after implantation could provide more insight in this respect. Furthermore, it might provide more granular information on causal relationships, which is valuable for clinical purposes.

Third, the speech perception measures used and the mode of presentation might explain inconsistent findings. Many studies reported cognitive measures to be related to sentence perception outcomes (in noise), rather than word perception outcomes (in quiet). However, it is unclear whether adding noise to words or sentences causes particular cognitive skills to be engaged, as many studies measure words in quiet and sentences in noise only. Measuring all four possible conditions might also be important to create a general classification system for better and poorer performers, which in turn can help to better generalize results. For example, it has been observed that poorer performers in quiet are poorer performers in noise, but better performers in quiet might be poorer performers in noise (Walia et al., 2022). Understanding the underlying causes leading to either poor performance in quiet or noise is needed, as this might lead toward different treatment options. In addition to the speech perception task, the extent to which bottom-up information during this task is not accessible, might also lead to the use of different cognitive strategies. Therefore, including measures of auditory sensitivity (as in Moberly et al., 2021) might be valuable. Furthermore, presentation mode (whether speech is presented in CI alone, or best aided condition) should be clearly stated. Unfortunately, this is overlooked in many of the included papers. Therefore, based on the included literature, it is impossible to make any inferences regarding the influence of listening condition on the use of specific cognitive skills. Indicating the test conditions in detail, or even including different testing conditions in future studies, like Hua et al. (2017), might be insightful.

Fourth, as mentioned in the introduction, the different cognitive domains and factors are not independent of each other. In fact, some tasks are used specifically to measure two different cognitive factors. Therefore, results based on correlation analysis, whereby each of the cognitive task scores are correlated separately with speech perception outcomes, should be interpreted with caution. It might be more informative to more often use alternative statistical analyses, such as regression analysis, instead. This could reveal any mediation of specific cognitive factors or cognitive scores explaining more of the variance in speech perception outcomes. Furthermore, as discussed before, many of the included studies do not report their power calculations, nor do they provide all statistical values. Ensuring sufficient power and consistently reporting statistical values (including effect sizes and values of non-significant results) will improve interpretation of results.

Lastly, while it is useful to look at papers which investigate either brain activation patterns or performance on cognitive and language tasks, it could be highly valuable to combine both neuroimaging techniques and behavioral measures within studies. We believe this could be beneficial as these measures could validate each other, as well as provide information on underlying neurocognitive processes involved in the observed behavior.

### 4.5. Limitations

The main limitation of this scoping review is that the number of papers for several neurocognitive domains is limited and their methods and dependent/independent variables are highly variable. Conclusions, therefore, are only based on a limited number of papers that cover the same cognitive domain or function. Secondly, as stressed previously, many questions remain as to why results of different papers do not agree. Furthermore, no risk of bias assessment was performed. This makes the conclusions drawn prone to being influenced by biases, whether coming from the authors of the included literature or from interpretation by the authors of this scoping review. Implementing the above-mentioned suggestions could improve evidence in future research and bring more clarity on the topics discussed in this review.

## 5. Conclusion

In this scoping review, a comprehensive overview of literature on the relationship between cognitive factors and speech perception outcomes in adult CI users was given. This literature showed that the use of higher-order cognitive functions, recruiting the frontal cortex, the use of visual cues, recruiting the occipital cortex, and the temporal cortex still available for auditory processing, are beneficial for postlingually deafened adult CI users in relation to speech perception outcomes. Cognitive assessments indicate that performance on non-verbal intelligence tasks positively correlated with speech perception outcomes. Performance on auditory or visual working memory, learning, memory and vocabulary tasks were unrelated to speech perception outcomes and performance on the Stroop task unrelated to word perception in quiet. However, many uncertainties regarding the explanation of inconsistent results and the small number of studies limit the extent of these conclusions. Additional research is needed to validate current findings. Only then will they potentially be used as a guide for counseling and rehabilitating adult CI users.

## Author contributions

LB, BP, EM, and WH contributed to the conception and design of the study. LB and NT collected the data. LB, NT, BP, and WH curated and interpreted the data. LB wrote the first draft of the manuscript. NT, BP, EM, and WH revised the manuscript critically. All authors read and approved the submitted version.
