# Supplementary material for: Exploring neurocognitive factors and brain activation in adult cochlear implant recipients associated with speech perception outcomes—A scoping review
Source: Front Neurosci. 2023 Feb 2;17:1046669. doi: 10.3389/fnins.2023.1046669 (PMC9932917; doi:10.3389/fnins.2023.1046669)
Supplement: Supplementary file 1 [file Data_Sheet_1.pdf]

Supplementary Material

Supplementary materials part A - Search strategies

1.1 PubMed

((Cochlear implants [MeSH] OR Cochlear implantation [MeSH] OR Cochlear implant* [tiab] OR Cochlear prosthes* [tiab] OR Auditory prosthes*[tiab])
OR
((Hearing Aids [MeSH] OR Hearing aid* [tiab]) AND (severe [tiab] OR profound [tiab])))
AND
(Adult* [tiab] OR postlingual* [tiab] NOT(Child[MeSH] NOT Adult [MeSH]))
AND
(Auditory perception [MeSH] OR Hearing [MeSH] OR Speech perception [tiab] OR Auditory perception* [tiab] OR Hearing [tiab] OR Audition [tiab] OR Speech discrimination [tiab] OR Speech recognition [tiab] OR Speech intelligibility [tiab] OR speech-in-noise performance [tiab] OR auditory performance [tiab] OR Word recognition [tiab] OR Speech reception [tiab] OR Sentence recognition [tiab] OR Listening Effort [tiab] OR Speech processing [tiab] OR Speech understanding [tiab] OR Speech outcomes [tiab] OR speech performance [tiab] OR CVC words [tiab] OR phoneme discrimination [tiab])
AND

(Cognition [MeSH] OR Cognition* [tiab] OR Cognitive function* [tiab] OR Cognitive predictor* [tiab] OR Neurocognit* [tiab] OR Cognitive impairment* [tiab] OR Cognitive Abilities [tiab] OR Cognitive processing [tiab] OR Cognitive skills [tiab] OR Cognitive performance [tiab] OR Top-down processing [tiab] OR Cognitive factors [tiab]
OR
Executive function [MeSH] OR Strategic planning [MeSH] OR Decision making [MeSH] OR Memory, Short-term [MeSH] OR Inhibition, Psychological [MeSH] OR Feedback [MeSH] OR Feedback, sensory [MeSH] OR Reactive inhibition [MeSH] OR Executive function* [tiab] OR Executive control*[tiab] OR Strategic planning [tiab] OR Decision making [tiab] OR Working memor* [tiab] OR Short-term memor* [tiab] OR Shortterm memor* [tiab] OR Immediate recall* [tiab] OR Immediate memor* [tiab] OR Inhibition* [tiab] OR inhibitory control [tiab] OR Nonverbal reasoning [tiab] OR Reactive Inhibition [tiab]
OR
Attention [MeSH] OR Attention [tiab] OR Processing speed [tiab]
OR
Psychomotor performance [MeSH] OR Visual perception [MeSH] OR Psychomotor performance* [tiab] OR Visual motor coordination*[tiab] OR Perceptual motor performance*[tiab] OR Sensory motor performance* [tiab] OR Visual perception*[tiab] OR Perceptual motor function* [tiab] OR Visuoconstructional reasoning [tiab] OR Perceptual motor coordination [tiab]
OR
Linguistics [MeSH] OR Linguistic* [tiab] OR Context* [tiab] OR Object naming [tiab] OR Word finding [tiab]
OR
Learning [MeSH] OR Memory [MeSH] OR Mental recall [MeSH] OR Memory, long-term [MeSH] OR Recognition, Psychology [MeSH] OR Learning [tiab] OR Memory [tiab] OR

recall [tiab] OR Long term memor* [tiab] OR Remote memor* [tiab] OR Recognition [tiab] OR Plasticity [tiab]

OR

Theory of mind [MeSH] OR Theory of mind [tiab] OR social perception [tiab] OR emotion recognition [tiab])

1.2 EMBASE

((exp Cochlea prosthesis/ OR exp Cochlear implantation/ OR (Cochlear implant* OR Cochlear prosthes* OR Auditory prosthes*).ti,ab,kw.)

OR

((exp Hearing Aid/ OR Hearing aid*.ti,ab,kw.) AND ((severe OR profound).ti,ab,kw.)))

AND

((Adult* OR postlingual*).ti,ab,kw. NOT (exp Child/ NOT exp Adult/))

AND

(exp hearing/ OR (Speech perception OR Auditory perception* OR Hearing OR Audition OR Speech discrimination OR Speech recognition OR Speech intelligibility OR speech-in-noise performance OR auditory performance OR Word recognition OR Speech reception OR Sentence recognition OR Listening Effort OR Speech processing OR Speech understanding OR Speech outcomes OR speech performance OR CVC words OR phoneme discrimination) .ti,ab,kw.)

AND

(exp Cognition/ OR exp Cognition assessment/ OR (Cognition* OR Cognitive function* OR Cognitive predictor* OR Neurocognit* OR Cognitive impairment* OR Cognitive Abilities OR Cognitive processing OR Cognitive skills OR Cognitive performance OR Top-down processing OR Cognitive factors).ti,ab,kw.

OR

exp Strategic planning/ OR exp Decision making/ OR exp Inhibition psychological/ OR exp psychological feedback/ OR exp sensory feedback/ OR exp Nonverbal reasoning/ OR (Executive function* OR Executive control* OR Strategic planning OR Decision making OR Working memor* OR Short-term memor* OR Shortterm memor* OR Immediate recall* OR Immediate memor* OR Inhibition* OR inhibitory control OR Nonverbal reasoning OR Reactive Inhibition).ti,ab,kw.

OR

exp Attention/ OR (Attention OR Processing speed).ti,ab,kw.

OR

exp Psychomotor performance/ OR exp Vision/ OR (Psychomotor performance* OR Visual motor coordination* OR Perceptual motor performance* OR Sensory motor performance* OR Visual perception* OR Perceptual motor function* OR Visuoconstructional reasoning OR Perceptual motor coordination).ti,ab,kw.

OR

exp Linguistics/ OR (Linguistic* OR Context* OR Object naming OR Word finding).ti,ab,kw.

OR

(Learning OR Memory OR recall OR Long term memor* OR Remote memor* OR Recognition OR Plasticity).ti,ab,kw.

OR

(Theory of mind OR social perception OR emotion recognition).ti,ab,kw.)

1.3 PsycINFO

(((Cochlear implants/ OR (Cochlear implant* OR Cochlear prosthes* OR Auditory prosthes*).ti,ab,id.)
OR
((Hearing Aids/ OR Hearing aid*.ti,ab,id.) AND ((severe OR profound).ti,ab,id.)))
NOT
(child* OR prelingual*).ti,ab,id.)
AND
(Auditory perception/ OR (Speech perception OR Auditory perception* OR Hearing OR Audition OR Speech discrimination OR Speech recognition OR Speech intelligibility OR speech-in-noise performance OR auditory performance OR Word recognition OR Speech reception OR Sentence recognition OR Listening Effort OR Speech processing OR Speech understanding OR Speech outcomes OR speech performance OR CVC words OR phoneme discrimination).ti,ab,id.)
AND

(Cognitive processes/ OR (Cognition* OR Cognitive function* OR Cognitive predictor* OR Neurocognit* OR Cognitive impairment* OR Cognitive Abilities OR Cognitive processing OR Cognitive skills OR Cognitive performance OR Top-down processing OR Cognitive factors).ti,ab,id.
OR
Memory/ OR Feedback/ OR Response inhibition/ OR (Executive function* OR Executive control* OR Strategic planning OR Decision making OR Working memor* OR Short-term memor* OR Shortterm memor* OR Immediate recall* OR Immediate memor* OR Inhibition* OR inhibitory control OR Nonverbal reasoning OR Reactive Inhibition).ti,ab,id.
OR
Attention/ OR (Attention OR Processing speed).ti,ab,id.
OR
Motor performance/ OR Visual perception/ OR (Psychomotor performance* OR Visual motor coordination* OR Perceptual motor performance* OR Sensory motor performance* OR Visual perception* OR Perceptual motor function* OR Visuoconstructional reasoning OR Perceptual motor coordination).ti,ab,id.
OR
Linguistics/ OR (Linguistic* OR Context* OR Object naming OR Word finding).ti,ab,id.
OR
Learning/ OR recall learning/ OR Recognition learning/ OR (Learning OR Memory OR recall OR Long term memor* OR Remote memor* OR Recognition OR Plasticity).ti,ab,id.
OR
Theory of mind/ OR (Theory of mind OR social perception OR emotion recognition).ti,ab,id.)

1.4 WEB of SCIENCE

TS=
((("Cochlear implant*" OR "Cochlear prosthes*" OR "Auditory prosthes*")
OR
("Hearing aid*" AND (severe OR profound)))
NOT(child* OR prelingual*)

AND
(“Speech perception” OR “Auditory perception*” OR Hearing OR Audition OR “Speech discrimination” OR “Speech recognition” OR “Speech intelligibility” OR “speech-in-noise performance” OR “auditory performance” OR “Word recognition” OR “Speech reception” OR “Sentence recognition” OR “Listening Effort” OR “Speech processing” OR “Speech understanding” OR “Speech outcomes” OR “speech performance” OR “CVC words” OR “phoneme discrimination”)
AND

(Cognition* OR “Cognitive function*” OR “Cognitive predictor*” OR Neurocognit* OR “Cognitive impairment*” OR “Cognitive Abilities” OR “Cognitive processing” OR “Cognitive skills” OR “Cognitive performance” OR “Top-down processing” OR “Cognitive factors”
OR
“Executive function*” OR “Executive control*” OR “Strategic planning” OR “Decision making” OR “Working memor*” OR “Short-term memor*” OR “Shortterm memor*” OR “Immediate recall*” OR “Immediate memor*” OR Inhibition* OR “inhibitory control” OR “Nonverbal reasoning” OR “Reactive Inhibition”
OR
Attention OR “Processing speed”
OR
“Psychomotor performance*” OR “Visual motor coordination*” OR “Perceptual motor performance*” OR “Sensory motor performance*” OR “Visual perception*” OR “Perceptual motor function*” OR “Visuoconstructional reasoning” OR “Perceptual motor coordination”
OR
Linguistic* OR Context* OR “Object naming” OR “Word finding”
OR
Learning OR Memory OR recall OR “Long term memor*” OR “Remote memor*” OR Recognition OR Plasticity
OR
“Theory of mind” OR “social perception” OR “emotion recognition”))

1.5 bioRxiv and medRxiv

advanced search → full search: cognition cochlear implant adult → find all words
advanced search → full search: memory cochlear implant adult → find all words
advanced search → full search: attention cochlear implant adult → find all words
advanced search → full search: executive function cochlear implant adult → find all words

Supplementary material part B

2.1 Supplementary Table 1

Table 1. An overview of the speech perception measures used in the included papers that assess cognitive or language skills before or after implantation in adults and relate this with speech perception outcomes.

Table 1. Description of speech perception measures

Measure	Description	Included papers using the measure
Word perception measures		
Consonant Nucleus Consonant (CNC) words	Open set word recognition.	(Hay-McCutcheon et al., 2005; Buckley and Tobey, 2010; Holden et al., 2013; Zhou et al., 2018; Dingemanse and Goedegebure, 2019; Wazen et al., 2020; Sun et al., 2021)
Consonant vowel consonant (CVC) words	Open set word recognition.<br>Versions: isophonemic and gated, NVA list.	(Collison et al., 2004; Kaandorp et al., 2015, 2017)
Words in Noise test (WIN)	Monosyllabic words with an adaptive procedure with initial value of 10 dB SNR changing with steps of 1 dB.	(Luo et al., 2022)
Freiburg Monosyllabic word test (FMWT)	Open set word recognition.	(Sandmann et al., 2012; Finke et al., 2016; Chen et al., 2017; Kessler et al., 2020; Knopke et al., 2021; Völter et al., 2021; Layer et al., 2022)
Korean phonetically balanced monosyllabic words	Open set word recognition.	(Song et al., 2015; Kim et al., 2016; Han et al., 2019)
French monosyllabic words	Open set word recognition	(Lazard et al., 2011)
Lafon test	Open set word recognition using a list composed of three-phoneme monosyllabic words.	(Lazard et al., 2010, 2013)
Swedish phonemically balanced word list	Open set word recognition. Monosyllabic words in a carrier phrase.	(Hua et al., 2017)
French disyllabic words	Open set word recognition for French disyllabic words obtained from the commonly-used French speech therapist list developed by Fournier.	(Strelnikov et al., 2013)
Italian phonetically balanced disyllabic words	The lists were extracted from one of the tests that, at the time of the evaluation, were most commonly used to assess speech intelligibility in the Italian language.<br>Condition: quiet	(Zucca et al., 2022)
Digit Triplet Test (DTT)	Digits triplets were presented in quiet and LTASS masking noise. The test score in quiet is the percentage of digit-triplets repeated. In the noise condition the noise level when repetition was 50% was the test score.	(Kaandorp et al., 2015, 2017)
Hard and easy isolated words	Lexically easy, and lexically hard words, based on frequency and density characteristics of the PB/MRT corpus (Balota et al., 2007).	(Tamati and Moberly, 2021)
Sentence perception measures		
Central institute for the deaf (CID) words & sentences	Standardized open-set word and sentence test.	(Suh et al., 2015; Moberly et al., 2018b, 2018a, 2021; Pisoni et al., 2018; Skidmore et al., 2020; Zhan et al., 2020)
Words in long complex sentences	Five word highly meaningful sentences following a subject-predicate structure. (from HINT originally).	(Moberly et al., 2017b)
AzBio sentences	Sentences of 4-12 words, with different target words, score is percentage correct (Spahr et al., 2012).<br>Conditions: quiet & babble noise	(Hillyer et al., 2019; Wazen et al., 2020; Zhan et al., 2020; Luo et al., 2022; Ray et al., 2022; Walia et al., 2022)
Hearing in Noise Test (HINT)	A large set of sentence materials, uniform in length and representation of natural speech with adaptive sSRTs.<br>Conditions: Quiet and noise SNR, +10, +5, speech shaped noise	(Hay-McCutcheon et al., 2005; Buckley and Tobey, 2010; Moberly et al., 2016; Hua et al., 2017)
Words in sentences from HINT	Five-word, highly meaningful sentences	(Moberly et al., 2017a, 2017b)
Oldenburg matrix sentences (OLSA) test	All test sentences follow the same syntactical structure, but are semantically unpredictable.<br>Conditions: Quiet and noise	(Haumann et al., 2012; Sandmann et al., 2012; Chen et al., 2016, 2017; Knopke et al., 2021)

City University of New York (CUNY) sentences	Sentences representative of everyday conversation.<br>Conditions: quiet and noise	(Hay-McCutcheon et al., 2005; Zhou et al., 2018; Skidmore et al., 2020)
Perceptually Robust English Sentence Test Open-set (PRESTO) sentence test	High-variability complex sentences with percentage words correct and sentences correct (Gilbert et al., 2013).	(Moberly et al., 2017c, 2018a, 2018b, 2021; Mattingly et al., 2018; Pisoni et al., 2018; Skidmore et al., 2020; Tamati et al., 2020, 2021; Zhan et al., 2020; Bosen et al., 2021)
Harvard sentences	Long, complex and semantically meaningful sentences (IEEE) consisting of an imperative or declarative structure.<br>Conditions: standard or anomalous in quiet	(Moberly et al., 2017c, 2018b, 2018a, 2021; Mattingly et al., 2018; Pisoni et al., 2018; Skidmore et al., 2020; Zhan et al., 2020; Tamati et al., 2021)
IEEE sentences	Long, complex and semantically meaningful sentences.	(O'Neill et al., 2019; Tinnemore et al., 2020)
Quick Speech perception In Noise test (QuickSIN)	A nonadaptive test that computes the SNR (25-0 in steps of 5 dB) necessary for 50% correct key word recognition in a background of four-talker babble noise (Killion et al., 2004).	(Mussoi and Brown, 2019)
Hochmair Schulz Moser (HSM) sentences test	Everyday German sentences with speech shaped noise.	(Finke et al., 2016; Kessler et al., 2020)
Göttinger sentence test	A German sentence test in noise. The sentences are highly equivalent in their performance intensity curves for normal hearing participants and number of words and phonemes (Kollmeier and Wesselkamp, 1997).	(Kessler et al., 2020)
VU98 sentences in quiet and noise	Dutch sentences from newspapers (Versfeld et al., 2000).<br>Conditions: quiet and 40 dB noise.	(Kaandorp et al., 2015, 2017; Dingemanse and Goedegebure, 2019)
Dyslexia test sentences	Long syntactically complex sentences designed to assess comprehension of complex syntax in children with dyslexia. The sentences contain three types of syntax: compound clauses, subject-object and object-subject.	(Moberly et al., 2016)
Helen test	Participants are asked simple questions to which they must respond.	(Mortensen et al., 2006)
Nonsense sentences	Sentences consisting of an article noun, verb, article, noun or verb, article, noun preposition article noun. With three key words per sentence. In quiet condition.	(Moberly et al., 2016, 2017b; O'Neill et al., 2019)
Meaningful and anomalous sentences in quiet	Sentences that included semantic context and sentences without.	(Moberly and Reed, 2019)

2.2 Supplementary Table 2

Table 2. An overview of the papers included in the review measuring brain activation. For each paper subject details, the task, speech perception outcomes measure, statistical test and key findings are reported.

Table 2. Included articles measuring brain activation.
ns = nonsignificant results reported

**Article**	**Participants (sample size, mean age in years at testing, duration of CI use)**	**Cognitive measure**	**Speech perception measure**	**Statistical test, (y/n) indicating a power analysis**	**Key findings**
(Mortensen et al., 2006)	Nhigh performing =7 → 96-100% standard open-set score without lipreading. N low performing =5 → less than 60% open-set score without lipreading. Mean age= 48.4 (range 35.0-64.0) Duration of CI use = 3.4 years (range 0.6-10.0)	PET during several speech stimuli	Helen test	t-test of high performing vs low performing group (n)	(+) The better performers showed more activation in the left inferior prefrontal and right anterior and posterior temporal cortex and the right cerebellum. (-) The poorer performers showed more activation in the left temporal areas. $p<0.05$
(Buckley and Tobey, 2010)	N=12 Mean age= 53.0 (range 37.0-63.0) CI use = 4.9 years (range 0.5-14.0)	EEG (N1, VEP) during presentation of visual gradients	CNC words<br><br>HINT-Q, SNR 10, 5 sentences	Linear regression analysis of the word and sentence scores against the amplitude of the N1 response. ROI: temporal lobe. (y)	*(ns) r=0.1618, p=0.6155*
(Lazard et al., 2010)<br><br>fMRI preoperatively	N=7 (same participants as (Lazard et al., 2011, 2013) Mean age= 52.0 (range 31.7-73.0) CI use = 6 months	fMRI during a phonological rhyming task and word categorization task	Lafon test	Multiple regression analysis between fMRI data and phonological performance on a reading task, duration of deafness and hearing loss and word recognition scores. (n)	(+) During the phonological task the left frontal, parietal, posterior temporal and occipital cortex (-) During the phonological task the anterior temporal, inferior frontal and right supramarginal gyrus. $p<0.001$ uncorrected
				Poor vs good performers based on Lafon test t-test (n)	(+) Dorsal regions and bilateral occipital regions more activated in better performers. (-) Bilateral ventral network (anterior temporal lobe, inferior frontal cortex and left temporal occipital junction) and right supramarginal gyrus more activated in poorer performers.
(Lazard et al., 2011)	N=10	fMRI during a visual imaging		Regression analysis (n)	(+) During sound imagery activity in the left inferior

fMRI preoperatively	(same participants as (Lazard et al., 2010, 2013)) Mean age= 53.0 (range 25.5-73.0) CI use = 6 months	task of colours and sounds	Three phoneme monosyllabic French words		frontal gyrus was positively correlated with speech perception r=0.94, p=0.0001
				Poor vs good performers based on word perception t-test (n)	(+) The dorsal fronto-parietal and occipital regions more activated in better performers.<br>(-) The ventral network (bilateral medial temporal lobes incl hippocampal gyrus) more activated in poorer performers..
(Sandmann et al., 2012)	N=11 Mean age= 54.0 (range 38.0-69.0) CI use = 76.0 months (range 12.0-240.0)	EEG (P100, N150, P270) during presentation of visual checkerboard patters	Freiburg Monosyllabic word test (FMWT)<br><br>Oldenburg sentence test SNR 50% (OLSA)	Spearmans rank correlations between ERPs and speech perception (n)	FMWT:(-) Right auditory cortex level 3 r=-0.78, p<0.05, level 4: r=-0.75, p<0.05 for right implanted participants<br><br>OLSA: (+) Right auditory cortex level 3: *(ns) r=0.63, p=0.07*, level 4: r=0.72, p<0.05
(Strelnikov et al., 2013)	N=10 Mean age= 53.9 (range 35.0-81.0) CI use = 7.6 days (range 1.0 – 22.0)	PET during auditory and visual words vs nonword presentation	French disyllabic words	Regression analysis and correlation analysis with FEW correction p<0.05 (n)	(+) The right occipital cortex during rest: r=0.9, during visual stimuli: r=0.8 and audiovisual stimuli: r=0.5, p<0.05, In the left inferior frontal pole during rest r=0.809, visual stimuli: r=0.77 and audiovisual stimuli: r=0.90 p<0.05<br>(-) In the middle STG/STS and occipital cortex during rest: r=-0.9, visual stimuli: r=-0.8 and audiovisual stimuli: r=-0.7, p<0.05
(Song et al., 2015)	N=10 Mean age= 31.5 (range 19.0-47.0) CI use = 57.8 months (range 6.0-147.0)	PET during video with a speaker saying digits in auditory, visual and audiovisual condition congruent and incongruent - preoperatively	Korean phonetically balanced monosyllabic words	Correlation analysis between contrast images of each condition and word perception scores. Controlled for sex and age. P=0.001 threshold. (n)	(+) During congruent audiovisual stimuli the amygdala rho = 0.888, p=0.008<br>(-) During congruent audiovisual stimuli the left rho=-0.826, p=0.013 and right rho=-0.777, p=0.019 occipital gyrus
(Suh et al., 2015)	N=15 Poor vs good 80% CID words Mean age= 64.0 (range 60.0-80.0) CI use = 1 year	PET during noise	CID words<br>Sentence perception	Mann-Whitney U test for difference in means (n)	(+) inferior temporal gyrus and premotor area in better performers p=0.0005<br>(-) Occipital area in poorer performers p=0.01
(Kim et al., 2016)	N=14 divided in 2 based on score on the korean phonetically balanced word perception test. Above 60% (N=7) as good and below 40%	EEG (VEP) while patterned visual stimuli are presented	Korean phonetically balanced monosyllabic words	Spearman correlation analysis between words scores and amplitude and latency of P1 in ROIs ROI: occipital and temporal electrodes (n)	(+) Larger P1 amplitude in occipital cortex r=0.755, p=0.001<br>Central visual field size r=0.699, p=0.009<br>(-) Larger P1 in right temporal cortex r=-0.736, p=0.003
				Mann-whitney test to compare means per group (n)	(+) P1 in occipital cortex larger p=0.013 in better performers<br>(-) P1 in right temporal cortex smaller in better performers p=0.002

	(N=7) as poor<br>Mean age= 44.8 (range 22.0-68.0)<br>CI use = 78.8 months (range 23.0-102.0)				
(Chen et al., 2016)	N=19 (same participants as (Chen et al., 2017))<br>Mean age= 54.5 (range 24.0-77.0)<br>CI use = 5.03 years (range 0.5-16.0)	fNIRS during visual checkerboard stimuli and auditory stimuli	Oldenburg sentence test (OLSA) in quiet and noise	Pearsons correlation analysis between activation differences condition and OLSA<br>ROI: right occipital cortex & left, right temporal cortex (n)	(+) r=0.518, p=0.027
(Chen et al., 2017)	N=19 (same participants as (Chen et al., 2016))<br>Mean age= 54.5 (range 24.0-77.0)<br>CI use = 5.0 years (range 0.5-16.0)	fNIRS during visual checkerboard stimuli and auditory word and reversed word stimuli	Freiburg Monosyllabic word test (FMWT)<br><br>Oldenburg sentence test (OLSA) in quiet and noise	Spearman correlation analysis between cross modal activation and speech recognition.<br>ROI: temporal and occipital cortex (n)	(+) FMWT: More cross modal plasticity for auditory than for visual stimuli r=0.525, p=0.021<br><br>(*ns*)OLSA:
(Lazard and Giraud, 2017)<br><br>Cognitive measure preoperatively	N=11<br>Mean age= 49.0 (range 20.8-78.7)<br>CI use = 6 months	fMRI during visual rhyming decision task - preoperatively	Response time, not accuracy on French word perception	Correlation between occipital-temporal coupling and speech perception. (n)	(+) Better performers: left posterior STG/STS<br>(-) Poorer performers: left and right fronto-parietal regions, left visual cortex , right posterior STS, right visual cortex. p<0.001 uncorrected
(Zhou et al., 2018)	N=15<br>Mean age= 64.2 (range 46-79)<br>CI use = 63.5 months (range 14.0-95.0)	fNIRS during audio, visual and audiovisual speechreading	CNC words<br>CUNY sentences in quiet and noise	Pearson correlation between activation levels and speech test scores.<br>ROI: STS/STG (n)	(-) Left STS and STG r=-0.668, p=0.009
(Han et al., 2019)	N=27<br>Mean age= 44.5 (range 22.0-60.0)<br>CI use = 1 year	PET during noise, no instruction (preop)	Korean phonetically balanced monosyllabic words	Pearson correlation between change in brain metabolism (p=0.001) and speech test scores. (n)	(+) Dorsolateral and dorsomedial frontal areas r=0.595, p>0.001<br>(-)Superior occipital gyrus r=-0.538, p<0.001
(Kessler et al., 2020)	N=21<br>Divided in two groups based on performance Gottingen sentence test cut-off 7.6 dB SNR<br>Mean age= 62.1(range 30.0-80.0)<br>CI use = 99.4 months (range 11.0-346.0)	SPECT scan & EEG during semantic correct vs incorrect sentences<br>(See also cognitive test table)	Freiburg Monosyllabic word test (FMWT)<br>HSM sentence test in quiet and noise 10 dB<br>Göttingen sentence test with adaptive noise	Independent T-test and difference images (n)	Göttingen sentence test groups:<br>(+) Better performers show higher activation in the left occipital area and right temporal area (p<0.001) during task.<br>(-) Poorer performers show higher activation in the left and right frontal BA9 and left ITG (p<0.001) during task.
(Sun et al., 2021)	N=94	MRI scan looking at grey matter –	CNC words	Clusters with random forest regression	(+) Left medial temporal cortex r=0.42, p<0.05

	Mean age= not reported for this group<br>CI use = 183.2 months (range 40.0-352.0)	cortical reorganisation		Vector machine regression as a linear method (n)	(-) Left superior temporal cortex r=-0.32, bilateral thalami r=-0.049, p<0.05
(Knopke et al., 2021)	Nyoung50-70= 26<br>Nold<70=23<br>Mean age= 67.3 , standard deviation = 8.7<br>CI use = 6, 12 and 24 months	White matter lesions with Fauzekas score	Freiburg Monosyllabic word test (FMWT)<br><br>Oldenburg sentence test (OLSA) in quiet and noise	Regression analysis with backward elimination (n)	(+) Lesions are a significant predictor of speech perception in quiet in younger group. 27.4%, p<0.05<br>*(ns)* older group
(Layer et al., 2022)	N=17<br>Mean age= 59 (range 27-75)<br>CI use = 72.1 months (range 15.0-236.0)	EEG during visual, auditory and audiovisual “ki” and “ka” from Oldenburg logatome speech corpus	Freiburg Monosyllabic word test (FMWT)	Pearson correlation with Benjamin Hochberg procedure for multiple comparisons (n)	*(ns) L*eft auditory cortex activation and speech perception. *r=0.27, p=0.29*

2.3 Supplementary Table 3

Table 3. An overview of the cognitive tasks used in the included papers that assess cognitive or language skills before or after implantation in adults and relate this with speech perception outcomes.

Table 3. Description of cognitive measures

Measure	Description	Included papers using the measure
General cognitive tests		
Mini-Mental State Examination (MMSE)	Short cognitive screening test consisting of 11 items (Folstein et al., 1975).	(Zucca et al., 2022)
Self-administered Gerocognitive Examination (SAGE)	22 point gerocognitive examination detecting early dementia and mild cognitive impairment (Scharre et al., 2010).	(Wazen et al., 2020)
Clock drawing test	The participant needs to draw a clock. The drawing is scored on different criteria (Shulman, 1999).	(Zucca et al., 2022)
Montreal Cognitive Assessment (MoCA)	Short screening instrument for measuring cognitive decline.	(Walia et al., 2022)
Complex attention		
Attention and concentration		
Leiter-3 attention sustained	Participants need to cross out as many figures as possible on a piece of paper that matches a target figure shown at the top of the page.	(Moberly et al., 2016, 2017b)
Woodcock-Johnson IV (WJ-IV) Letter and number pattern matching, pair cancellation task	The participant locates and circles two identical letter patterns or numbers in a row of six patterns or numbers.<br>The participant locates and marks a repeated pattern as quickly as possible within 1 minute time limit.	(Hillyer et al., 2019)
ALAcog M3 attentional task	The participant has to identify a target letter by clicking as fast as possible and ignore distractors (Völter et al., 2017).	(Völter et al., 2021)
Trail Making Test-B (TMT-B) featured in: Alacog test battery	The participant must connect circles from 1-A-2-B-3-C etc. The time is measured (Völter et al., 2017).	(Hua et al., 2017; Völter et al., 2021; Zucca et al., 2022)
Processing speed		
Woodcock-Johnson IV (WJ-IV) Letter and number pattern matching, pair cancellation task	See attention and concentration	See attention and concentration
Wechsler Adult intelligence scale III (WAIS-III) symbol search test	The participant must view rows of symbols and target symbols, and mark whether the target symbols appear in each row. As many sets as possible in 2 minutes.	(Mussoi and Brown, 2019)
National Institutes of Health (NIH) toolbox pattern comparison processing speed test	Participants must identify whether two visual patterns are the same or not by pressing a button. Patterns were identical or varied based on colour or parts of an object (Cognition assessment using the NIH Toolbox, 2013).	(Tinnemore et al., 2020)
Wechsler Adult intelligence scale III (WAIS-III) coding test	The participant needs to transcribe a digit-symbol code using a key. The task is time-limited.	(Mussoi and Brown, 2019)
Woodcock-Johnson IV (WJ-IV) numbers reversed and pictures test	The participant needs to recall a row of numbers or pictures in reversed order.	(Hillyer et al., 2019)
Trail Making Test-A (TMT-A) featured in: Alacog test battery	The participant must connect circles from 1-25 etc. The time is measured (Völter et al., 2017).	(Hua et al., 2017; Völter et al., 2021; Zucca et al., 2022)
Executive function		
Nonverbal intelligence		
Ravens Progressive Matrices (RPM)	The participant must identify the missing piece of a pattern within 3x3 matrices of geometric designs (Raven, 2000).	(Moberly et al., 2017c, 2018a, 2018d, 2021; Mattingly et al.,

		2018; Pisoni et al., 2018; Moberly and Reed, 2019; O'Neill et al., 2019; Skidmore et al., 2020; Tamati et al., 2020, 2021; Zhan et al., 2020; Tamati and Moberly, 2021)
Wechsler Adult intelligence scale III (WAIS-III) matrix reasoning test	The participant has to view an array of pictures with one missing and determine which picture of five options is the missing one.	(Holden et al., 2013)
Leiter-3 visual pattern test	Participants need to determine which shape comes next in a pattern of shapes.	(Moberly et al., 2016)
Test Of Nonverbal Intelligence-3 (TONI-3) pointing pictures	The participant has to identify the relation between abstract figures, which will be manipulated.	(Collison et al., 2004)
Leiter-3 figure ground	Participants need to find a target object within a bigger picture.	(Moberly et al., 2016)
Leiter-3 form completion	Participants need recognize the complete image of the target that is broken into different components.	(Moberly et al., 2016)
Working memory		
Visual		
Visual digit span Featured in: WJ-IV, WAIS-III.	Participants must repeat a row of digits presented to them, either in a forward or backward manner. The digit string was either increased until the participant was unable to remember correct numbers in correct sequence (Hillyer et al., 2019). Or items with increasing amounts of digits were scored.	(Moberly et al., 2017c, 2018a, 2018c, 2021; Moberly and Reed, 2019; Skidmore et al., 2020; Tamati et al., 2020, 2021; Zhan et al., 2020; Tamati and Moberly, 2021)
Woodcock-Johnson IV (WJ-IV) numbers reversed test and pictures	See processing speed.	See processing speed.
Leiter-3 forward and reverse memory	Participants need to remember the order of a set of pictures presented in forward or backward manner.	(Moberly et al., 2016, 2017b)
Wechsler Adult intelligence scale III (WAIS-III) visual object span	Participants must repeat a row of objects presented to them, either in a forward or backward manner.	(Moberly et al., 2017c; Skidmore et al., 2020; Zhan et al., 2020)
Visual letter span task	The participant had to remember the order of a set of letters (A,C,E,F,H,I,L, O and R). The span was defined as the length of the longest list of letters correctly recalled at least two times.	(Luo et al., 2022)
Wechsler Adult intelligence scale III (WAIS-III) visual symbol span	Participants must repeat a row of symbols presented to them, either in a forward or backward manner.	(Moberly et al., 2017c; Skidmore et al., 2020; Zhan et al., 2020)
Alacog 2-back test	Participants have to respond when a letter shown was the same as the second last (Völter et al., 2017).	(Völter et al., 2021)
Alacog Operation Span task (OSPAN)	A dual task where letters had to be memorized while at the same time equations had to be solved (Völter et al., 2017).	(Völter et al., 2021)
Auditory		
Auditory digit span Featured in: WAIS-III, MoCA	Participants must repeat a row of digits presented to them, either in a forward or backward manner.	(Holden et al., 2013; Moberly et al., 2017a;

		Bosen et al., 2021; Luo et al., 2022)
Auditory-visual		
Audiovisual digit span<br>Featured in: WJ-IV, WAIS-III.	Participants must repeat a row of digits presented to them, either in a forward or backward manner.	(Hillyer et al., 2019; Mussoi and Brown, 2019; Zucca et al., 2022)
Cued modality working memory task	Participants were cued or not cued to remember either auditory digits or visual letters or both (Luo et al., 2022).	(Luo et al., 2022)
Verbal		
Listening span	Participants must decide for sentences that they hear whether they are true or false. After each judgement a letter is presented. Then recall of these letters is asked (Moberly et al., 2017b).	(Moberly et al., 2017b)
Reading span	Participants must decide whether sentences that they see are true or false. After each judgement a letter was presented that they needed to recall, or they needed to remember the first or last letter of the sentences later.	(Hua et al., 2017; Kaandorp et al., 2017; Moberly et al., 2017b; Dingemanse and Goedegebure, 2019; O'Neill et al., 2019; Luo et al., 2022)
Size comparison span task (SicSpan)	Participants have to answer the question whether one item is bigger than the other. Between these questions a semantically related word is presented that participants have to remember (Sörqvist et al., 2010).	(Finke et al., 2016; Kessler et al., 2020)
Cognitive inhibition		
ALAcog Flanker task<br>Featured in: Alacog and NIH toolbox	Participants respond to a target, flanked by arrow pointers above, and underneath pointing in the same direction, which is the compatible Flanker or pointing into different directions which is the incompatible Flanker (Völter et al., 2017).	(Tinnemore et al., 2020; Völter et al., 2021)
Stroop task	Participant need to respond to the colour of a text and not to the text itself, which are names of colours. Stimuli can be congruent, where the colour of the text is the same as the word (e.g. "green" written in green ink) or incongruent where the text is a different colour than the word (e.g. "green" written in blue ink) (Moberly et al., 2016).	(Moberly et al., 2016, 2017b, 2018b, 2018a, 2021; Moberly and Reed, 2019; Skidmore et al., 2020; Tamati et al., 2020, 2021; Zhan et al., 2020; Tamati and Moberly, 2021)
Flexibility		
Trail Making Test – B (TMT-B)<br>Featured in: Alacog	See attention and concentration	See attention and concentration
National Institutes of Health (NIH) toolbox dimensional change card sort (DCCS) test	Participants are asked to match a series of pictures to target pictures based on one dimension. This dimension switches over time (Zelazo et al., 2014b).	(Tinnemore et al., 2020)
Social cognition		
Not applicable		
Learning and memory		
Recognition memory		
Woodcock-Johnson IV (WJ-IV) picture recognition test	The participant needs to recognize a subset of previously presented pictures amongst distracting pictures.	(Hillyer et al., 2019)
Verbal learning and recall		
Auditory word recall (and delayed recall) task<br>Featured in: MoCA, Rey 15 words auditory learing test (RAVLT), Alacog	The participant needs to recall a set of words presented to them.	(Völter et al., 2021; Zucca et al., 2022)

CERAD-plus test battery	A testbattery including verbal fluence tests, naming tests, mini mental state examination and word learning.	(Kessler et al., 2020)
California Verbal learning test (CVLT) -II	Participants need to recall al list of 16 words (list A) five times. The words belong to 4 semantic categories. Then an interference list (list B) is presented that needs to be recalled. This list shares two of four semantic categories with list A. After this, participants are asked to recall list A (short-delay free recall). Then a short-delay cued recall task is performed. From these scores additional scores are calculated that reflect leaning slope, recall consistency, primacy recall, recency recall, serial clustering, semantic clustering and subjective clustering (Ray et al., 2022).	(Holden et al., 2013; Pisoni et al., 2018; Skidmore et al., 2020; Tamati et al., 2020; Ray et al., 2022)
Perceptual motor function		
Woodcock-Johnson IV (WJ-IV) visualization parts A and B	The participant needs to identify parts that form a complete target shape and needs to identify two block patters that match a target pattern..	(Hillyer et al., 2019)
Corsi block tapping test	Participants need to remember which of nine blocks on a screen lighted up and repeat the pattern. This also involves working memory.	(Zucca et al., 2022)
Block rotation task	Participants need to match a target figure consisting of 3D blocks and match it with the corresponding figure that is rotated.	(Hillyer et al., 2019)
Language		
Object naming & word finding (vocabulary)		
Picture naming task Featured in/versions: Expressive vocabulary test (EVT), Peabody Picture Vocabulary Test (PPVT), MoCA linguistic naming, Rapid automatic naming (RAN), WJ-III Verbal Comprehension Section, WAIS vocabulary test.	Participants view a set of pictures, symbols, letters or digits and have to name, describe or provide a synonym for them.	(Collison et al., 2004; Holden et al., 2013; Kaandorp et al., 2015; Völter et al., 2021)
Choosing a synonym task Featured in/versions: WJ-III Verbal Comprehension Section, Groningen intelligence test II vocabulary size subtest.	Participants have to identify a synonym of a given word.	(Collison et al., 2004; Kaandorp et al., 2015, 2017)
Word naming test	Recognizing and pronouncing a word (de Groot et al., 2002).	(Kaandorp et al., 2017)
Word vs nonword discrimination task Featured in/versions: Merfachwahl wortschatz inteligenz test (MWT-B), Lexical Decision task (LDT)	Participants have to indicate whether a stimulus that is presented is either a real word or a nonword.	(Finke et al., 2016; Kaandorp et al., 2017; Moberly et al., 2017a; Kessler et al., 2020; Völter et al., 2021)
WordFam-150 test	Participants have to indicate how familiar they are with the words presented to them.	(Pisoni et al., 2018; Skidmore et al., 2020; Bosen et al., 2021; Tamati et al., 2021)
Woodcock-Johnson III (WJ-III) Verbal Comprehension Section (VCS)	Participants have to do a verbal comprehension, picture vocabulary, synonyms, antonyms, analogies task.	(Collison et al., 2004)
Wechsler Adult intelligence scale III (WAIS-III) similarities test	Participants need to describe if two words are the same.	(Holden et al., 2013)
Verbal fluency		

Verbal Fluency Task Featured in/versions: Regensburger wortflussigkeits test (RWT), Phonological, Semantic, Alacog	Participants have 60 seconds to verbally list as many things as possible from one category. The category can be semantic (animals/fruits), or phonological (all words that begin with a particular letter).	(Finke et al., 2016; Kessler et al., 2020; Völter et al., 2021; Zucca et al., 2022)
Speed of Lexical and phonological access		
Test of word reading efficiency (TOWRE)	Participants need to read as many real words and non-words from lists as possible within 45 seconds.	(Moberly et al., 2018a, 2018d, 2021; Pisoni et al., 2018; Moberly and Reed, 2019; Skidmore et al., 2020; Tamati et al., 2020, 2021)
Wide Range Achievement Test (WRAT) word reading task	The participant needs to read aloud a list of letters or words.	(Skidmore et al., 2020; Tamati et al., 2021)
Speechreading sentences	CNC words, the HINT and CUNY sentence test were presented before implantation in auditory only, visual and audiovisual condition. Participants needed to respond how much they perceived for each condition.	(Hay-McCutcheon et al., 2005)
Lexical Model Oriented (LEMO) subtest of internal homophonic word reading	Participants need to internally read non-words and decide if it sounds like a real word or not.	(Völter et al., 2021)
Audiovisual non-word repetition task	Participants see and hear a talker saying a non-word and the participant needed to repeat the word immediately.	(Moberly et al., 2017a)
Degraded receptive language		
Text reception Threshold (TRT)	The TRT is a visual analogue of the SRT test, where visual sentences are degraded by different kinds of patterns. The score of the participant is based on the degree of degradation for a 50% word perception score (Zekveld et al., 2007).	(Haumann et al., 2012; Kaandorp et al., 2017; Völter et al., 2021)
Fragmented sentences test	Visually degraded meaningful sentences were briefly presented on a screen. The participant needed to read as much of the sentence as possible (Feld and Sommers, 2009).	(Moberly et al., 2018b)

2.4 Supplementary Table 4

Table 4. An overview of the papers included in the review performing cognitive tests. For each paper subject details, the task, speech perception outcomes measure, statistical test and key findings are reported.

Table 1. Included articles measuring cognitive or language skills
ns = nonsignificant results reported

**Article**	**Participants (sample size, mean age in years at testing, duration of CI use)**	**Cognitive measure**	**Speech perception measure**	**Statistical test, (y/n) indicating a power analysis**	**Key findings**
(Collison et al., 2004)	N=15<br>Mean age= 55.0 (range 34-68)<br>CI use = 5.7 years (range 0.8-12.0)	EVT picture naming	CVC words	Pearson correlations (n)	*(ns) r=0.501*
		TONI-3 pointing pictures	CVC words		*(ns) r=0.155*
		WJ-III Verbal Comprehension Section (VCS)	CVC words		*(ns) r=0.286*
(Hay-McCutcheon et al., 2005)<br><br>Cognitive measure preoperatively	Nage>65=17<br>Nage39-53 = 17<br>Mean age= 59.4 (range 39-83)<br>CI use = 6, 12 and 24 months	Speechreading CUNY sentences in auditory, visual, audiovisual conditions	CNC words<br>HINT in quiet and +10 dB SNR<br>CUNY in quiet	Pearson correlations (n)	(-)Young group: r=-0.872, p=0.002<br>*(ns)* Older group: *r=0.0562, p=0.189*
(Haumann et al., 2012)<br><br>Cognitive measure preoperatively	Nears=97<br>Mean age= 58.1, standard deviation=14.7<br>CI use = 6 months	Text Reception Threhold (TRT) with OLSA sentences	Oldenburg sentence test (OLSA) in noise	Correlation analysis and coefficient of determination as defined by Everitt to be the square of the correlation coefficient. (n)	OLSA unmodulated noise: (-) TRT random dots r=-0.23 P=0.036 r^2=0.05, TRT random bars r=-0.27, P=0.012, r^2=0.07,SN modulated: TRT random dots r=-0.29, P=0.007, r^2=0.09,TRT random bars r=-0.28, P=0.009, r^2=0.08 SN fixed: (+) TRT random noise r=0.26, P=0.026, r^2=0.07
(Holden et al., 2013)<br><br>Cognitive measure preoperatively	N=92<br>Divided in 6 groups based on rank order percentage 0-<10%, 10-<25%, 25-<50%, 50-<75%, 75-<90%, 90-<100% CNC word score<br>Mean age= 57.4 (range 23-83)<br>CI use =mostly 2,4,6 and 9 weeks, 3,6,9, 12, 15,18, 21 and 24 months	WAIS-III forward & backward digit span– preoperatively	CNC words	Non-parametric correlation and principal component measures. All measures taken together (n)	*(ns)* when corrected for age
		WAIS-III picture naming vocabulary test– preoperatively			
		WAIS-III matrix reasoning test– preoperatively			
		WAIS-III similarities– preoperatively			
		Calefornia Verbal Learning test (CVLT) – preoperatively			
(Kaandorp et al., 2015)	N=24<br>Mean age= 59.0 (range 30-82)<br>CI use =1=3.1 years (range 1.1-6.6)	Peabody Picture Vocabulary Test (PPVT)	CVC NVA words in quiet<br>VU98 sentences<br>DTT quiet and noise	Regression modelling both vocabulariy tests taken together (n)	*(ns)*
		Groningen intelligence test II			

		– vocabulary size subtest			
(Finke et al., 2016)	N=13<br>Mean age= 60.0 (range 43.0-75.0)<br>CI use =30.0 months (range 14.0-58.0)	Size comparison span task (SicSpan)	Freiburg Monosyllabic word test (FMWT)	Correlation analysis, df=11 (n)	*(ns)*
			HSM sentence test		*(ns)*
		Regensburger wortflussigkeits test (RWT)	Freiburg Monosyllabic word test (FMWT)		*(ns) r(11)=0.536, p=0.059*
			HSM sentence test		*(ns) r(11)=0.518, p=0.061*
		Merfachwahl wortschatz inteligenz test (MWT-B)	Freiburg Monosyllabic word test (FMWT)		*(ns)*
			HSM sentence test		*(ns)*
(Moberly et al., 2016)	N=30<br>(same participants as (Moberly et al., 2017b, 2017a))<br>Mean age= 68.4 (range 50.0-82.0)<br>CI use = 7.2 years (range 1-30)	Leiter-3 figure ground	Dyslexia test words in long complex sentences in noise	Pearson correlation (n)	*(ns) r=0.15*
			HINT-C words in short highly constrained meaningful sentences in noise		*(ns) r=0.13*
			Non-word sentences in quiet		*(ns) r=0.15*
		Leiter -3 form completion	Dyslexia test words in long complex sentences in noise		*(ns)r=-0.09*
			HINT-C words in short highly constrained meaningful sentences in noise		*(ns) r=-0.16*
			Non-word sentences in quiet		*(ns) r=-0.09*
		Leiter -3 visual pattern test	Dyslexia test words in long complex sentences in noise		*(ns) r=0.33*
			HINT-C words in short highly constrained meaningful sentences in noise		*(ns)r=0.26*
			Non-word sentences in quiet		*(ns)r=0.33*
		Leiter -3 attention sustained	Dyslexia test words in long complex sentences in noise		*(ns) r=0.14*
			HINT-C words in short highly constrained meaningful sentences in noise		*(ns) r=0.14*, noise: *r=0.19*
			Non-word sentences in quiet		*(ns)* non-word *r=0.29*
		Leiter -3 forward and reverse memory	Dyslexia test words in long complex sentences in noise		*(ns)* forward: *r=0.23* reverse: *r=-0.28*
			HINT-C words in short highly constrained meaningful sentences in noise		*(ns)* forward: *r=0.23* reverse: *r=0.20*
			Non-word sentences in quiet		*(ns)* forward: *r=0.23* reverse: *r=0.20*
		Stroop task	Dyslexia test words in long complex sentences in noise		(-) incongruent:r=-0.41, p<0.05,<br>*(ns)* congruent: *r=-0.28,*
			HINT-C words in short highly constrained meaningful sentences in noise		(-) incongruent: r=-0.43, p<0.05<br>*(ns)* congruent: *r=-0.36*
			Non-word sentences in quiet		(-) incongruent: r=-0.43, p<0.05 *(ns)* congruent: *r=-0.28*

(Hua et al., 2017)	N=17<br>Mean age= 53.5 (range 28-75)<br>CI use = 5.3 years (range 2.0 – 9.0)	Trail Making Test-A (TMT-A)	Swedish phonetically balanced words in quiet	Pearson correlations (also corrected for age) (y, but not sufficient)	*(ns)* CI only: *r=-0.27*, corrected for age: *r=-0.53* (-) Bimodal r=-0.60, p>0.05 *(ns)* corrected for age: *r=-0.48*
			HINT		*(ns) r=0.19*, corrected for age: *r=0.16*
		Trail Making Test-B (TMT-B)	Swedish phonetically balanced words in quiet		(-) CI only: r=-0.52, p<0.05, corrected for age: r=-0.53, p<0.05 Bimodal r=0.75, p>0.01 → corrected for age: r=-0.67, p<0.01
			HINT		(+) r=0.55, p>0.05 *(ns)* corrected for age r=0.46
		Reading Span	Swedish phonetically balanced words in quiet		(-) Bimodal r=-0.71, p>0.01 , corrected for age r=0.70, p<0.01, *(ns)* CI only: *r=0.44*, corrected for age: *r=0.42*
			HINT		*(ns)*: *r=-0.48*, corrected for age: *r=-0.44*
(Moberly et al., 2017a)	N=30<br>(same participants as (Moberly et al., 2016, 2017b))<br>Mean age= 68.4 (range 50.0-82.0)<br>CI use = 7.2 years (range 1.0-30.0)	Auditory digit span	Words and sentences in quiet and noise	Correlation analysis, df=28 (n)	*(ns)*
		Serial recall of words	Words and sentences in quiet and noise		*(ns)*
		Lexical decision task	Words and sentences in quiet and noise		*(ns)*
		Audiovisual non-word recognition	Words and sentences in quiet and noise		*(ns)*
(Moberly et al., 2017b)	N=30<br>(same participants as (Moberly et al., 2016, 2017a))<br>Mean age= 68.4 (range 50.0-83.0)<br>CI use = 7.2 years (range 1.0-30.0)	Reading span	Words in long complex sentences	Pearson correlation between Reading Span and Listening Span and sentence recognition scores (n)	*(ns) r=0.1*
			HINT-C words in short highly constrained meaningful sentences in noise		*(ns) r=-0.3*
			Non-word sentences in quiet		*(ns) r=-0.02*
		Leiter-3 attention sustained	Words in long complex sentences		*(ns): r=0.14*
			HINT-C words in short highly constrained meaningful sentences in noise		*(ns) r=0.19*
			Non-word sentences in quiet		*(ns) r=019*
		Listening Span	Words in long complex sentences		(+) r=0.64, p<0.01
			HINT-C words in short highly constrained meaningful sentences in noise		(+) r=0.57, p<0.01
			Non-word sentences in quiet		(+) r=0.68, p<0.01
		Leiter-3 forward and reverse memory	Words in long complex sentences		*(ns) r=0.23*
			HINT-C words in short highly constrained meaningful sentences in noise		*(ns) r=0.13*
			Non-word sentences in quiet		*(ns) r=0.14*
		Stroop task	Words in long complex sentences		(-) r=-0.41, p<0.05, also r with LSPAN IC→ LSPAN → SP
			HINT-C words in short highly constrained		(-) r=-0.43, p<0.05

			meaningful sentences in noise		
			Non-word sentences in quiet		(-): r=-0.43, p<0.05
(Moberly et al., 2017c)	N=30<br>Mean age= 67.3, standard deviation =8.1<br>CI use = 7.5 (range 1.0-30.0) , standard deviation =7.2	WAIS-III Visual digit span	PRESTO quiet	Partial correlation analysis, df=26 (n)	*(ns)*
			Harvard sentences quiet		(+) r(26)=0.40, p=0.035
		WAIS-III Visual object span	PRESTO quiet		*(ns)*
			Harvard sentences quiet		*(ns)*
		WAIS-III Visual symbol span	PRESTO quiet		*(ns)*
			Harvard sentences quiet		*(ns)*
		Ravens Progressive Matrices (RPM)	PRESTO quiet		*(ns)*
			Harvard sentences quiet		(+) r(26)=0.53, p<0.01,
(Kaandorp et al., 2017)	N=20 (4 poor performers on SIN excluded)<br>Mean age= 64.0 (range 50.0-80.0)<br>CI use = 3.3 years (range 1.0-8.0)	Vocabulary test – choosing a synonym	NVA CVC + VU98 Sentences in noise + DTT = averaged score	Correlation analysis and regression analysis. (n)	*(ns)* VU98: *r=-0.19,* WN: *r=-0.19,* SN: *r=-0.33,* SRTdiff: *r=-0.27*
		Lexical decision test	NVA CVC + VU98 Sentences in noise + DTT = averaged score		(+) SRTdiff: r=0.45, p=0.047, explained additional 36% in model *(ns)* VU98: *r=-0.25,* WN: *r=0.07,* SN: *r=038,*
		Word naming test	NVA CVC + VU98 Sentences in noise + DTT = averaged score		*(ns)* WQ: *r=-0.02,* WN: *r=-0.03,* SN: *r=0.12,* SRTdiff: *r=0.18*
		Text Reception Threshold (TRT)	NVA CVC + VU98 Sentences in noise + DTT = averaged score		*(ns)* WQ*: r=-0.22,* WN: *r=-0.19,* SN: *r=-0.33,* SRTdiff: *r=-0.27*
		Reading Span	NVA CVC + VU98 Sentences in noise + DTT = averaged score		(-) SN: r=-0.59, p=0.006, SRTdiff: r=-0.57, p=0.009, explained additional 46% *(ns)* WQ: *r=0.03,* WN: *r=-0.26*
(Mattingly et al., 2018)	N=39<br>Mean age= 67.5 (range 50.0-83.0)<br>CI use = 7.4 years (range 1.5-34.0)	Ravens Progressive Matrices (RPM)	PRESTO	Pearson correlations (n)	(+)words: r=0.41, p<0.01, sentence r=0.47, p<0.01,
			Harvard sentences		(+)words r=0.35, p<0.05, sentence r=0.46, p<0.01
(Moberly et al., 2018a)	N=42<br>(same participants as(Moberly et al., 2018b, 2021; Tamati et al., 2020))<br>Mean age= 67.3 (range 50.0-83.0)<br>CI use = 7.2 years, standard deviation = 6.7	WAIS-III visual digit span	PRESTO	Partial correlation analysis, controlled for age (n)	*(ns)* Controlled for age*:* words: *0.08* sentences: *r=0.17*
			Harvard sentences		*(ns)* Controlled for age*:* words *r=0.12,* sentences: *r=0.26*
			CID words		*(ns) r=0.09*
		Stroop task	PRESTO		*(ns)* words *r=-0.05,* sentences *r=-0.12*
			Harvard sentences		*(ns)* words *r=-0.13,* sentences *r=-0.23,*
			CID words		*(ns) r=0.12*
		TOWRE	PRESTO		(+) Words and PRESTO words, r=0.47, p<0.01, words and PRESTO r=0.54, p<0.01, non-words r=0.40, p<0.05, words and
			Harvard sentences		(+)Harvard words and words r=0.37, p<0.05, words and Harvard sentences: words r=0.57, p<0.05, non-words r=0.45, p<0.01
			CID words		(+) Words r=0.47, p<0.01
		Ravens Progressive Matrices (RPM)	PRESTO		(+)words: r=0.45, p<0.01, sentences: r=0.47, p<0.01,
			Harvard sentences		(+)sentences r=0.39, p<0.05
			CID words		(+) r=0.35, p<0.05

(Moberly et al., 2018b)	N=34 (same participants as(Moberly et al., 2018a, 2021; Tamati et al., 2020)) Mean age= 69.0 (range 50.0-80.0) CI use = 7.1 years (range 1.5-34.0)	WAIS-III visual digit span	Harvard sentences	Linear regression analysis (n)	*(ns)* $r^2=0.010$, $\beta=0.101$, $p=0.576$,
			PRESTO		*(ns)* $r^2=0.003$, $\beta=0.057$, $p=0.751$
			CID words		*(ns)* $r^2=0.005$, $\beta=0.068$, $p=0.704$
		Stroop task	Harvard sentences		*(ns)* $r^2=-0.085$, $p=0.099$,
			PRESTO		*(ns)* $r^2=0.017$, $p=0.468$
			CID words		*(ns)* $r^2=0.056$, $p=0.108$
		TOWRE	Harvard sentences		(+)words and PRESTO words $r^2=0.187$, $\beta=0435$, $p=0.011$
			PRESTO		(+) Words and Harvard words $r^2=0.175$, $\beta=0.418$, $p=0.015$
			CID words		(+) Words $r^2=0.312$, $\beta=0.558$, $p=0.001$ non-words $r^2=0.173$, $\beta=0.416$, $p=0.014$
		Ravens Progressive Matrices (RPM)	Harvard sentences		(+)words: $r^2=0.291$, $\beta=0.540$, $p=0.001$
			PRESTO		(+)words: $r^2=0.357$, $\beta=0.598$, $p<0.001$
			CID words		(+) $r^2=0.325$, $p<0.001$, $\beta=0.570$ (also mediated by age)
		Fragmented Sentences	Harvard sentences		*(ns)* $r^2=0.055$, $\beta=0.234$, $p=0.109$
			PRESTO		*(ns)* $r^2=0.11$, $\beta=0.334$, $p=0.058$
			CID words		(+) $r^2=0.157$, $\beta=0.396$, $p<0.001$
(Pisoni et al., 2018)	N=25 Mean age= 68.0 (range 53.0-81.0) CI use = 7.5, standard deviation = 6.7	CVLT-II	CID words	Correlational analysis and regression analysis (n)	(+) List B: $r=0.47$, $p<0.05$, added value to model
			Harvard sentences		(+) List B: words $r=0.48$, $p<0.05$, sentences: $r=0.56$, $p<0.05$ List A trial 5 and words: $r=0.46$, $p<0.05$. *(ns)* rest
			PRESTO		(+) List B: words: $r=0.52$, $p<0.05$, sentences $r=0.52$, $p<0.05$, *(ns)* rest
		Ravens Progressive Matrices (RPM)	CID words		(+) $r=0.64$, $p<0.05$, and additional value in model
			Harvard sentences		(+) words: $r=0.71$, $p<0.05$, sentences: $r=0.60$, $p<0.05$ and additional value in model
			PRESTO		(+) words: $r=0.62$, $p<0.05$, sentences: $r=0.68$, $p<0.05$, and additional value in model
		TOWRE	CID words		(+) TOWRE words: $r=0.55$, $p<0.05$, TOWRE nonwords: $r=0.41$, $p<0.05$.
			Harvard sentences		(+) TOWRE words: sentences $r=0.47$, $p<0.05$, TOWRE nonwords: words $r=0.49$, $p<0.05$, sentences $r=0.48$, $p<0.05$, *(ns)* rest
			PRESTO		(+) TOWRE words: words $r=0.41$, $p<0.05$, sentences $r=0.41$, $p<0.05$, TOWRE nonwords: sentences $r=0.48$, $p<0.05$ *(ns)* rest

		WordFam-150 test	CID words		*(ns)*
			Harvard sentences		*(ns)* words and sentences
			PRESTO		(+)sentences r=0.45, p<0.05 *(ns)* words
(O'Neill et al., 2019)	N=30<br>Mean age= 61.5 (range 20.0-80.0)<br>CI use = 10.4 years (range 1.0-28.0)	Reading span task	Average score of nonsense and context sentences together	Pearson correlations (n)	(+) r=0.430, p=0.018
		Ravens Progressive Matrices (RPM)	Average score of nonsense and context sentences together		*(ns) r=0.319, p=0.086*
(Hillyer et al., 2019)	N=21<br>Mean range = 52.0-88.0<br>CI use = 34.4 months (range 10.0-79.0)	WJ-IV visual number and picture span	AzBIO sentences in quiet	Pearson correlations, controlling for age (n)	(+) Controlling for age: CI only r=0.539, p=0.016
		WJ-IV spatial relations A & B visualization	AzBIO sentences in quiet		*(ns)*
		WJ-IV block rotation	AzBIO sentences in quiet		*(ns)*
		WJ-IV letter pattern matching, number pattern matching, pair cancellation	AzBIO sentences in quiet		*(ns)*
		WJ-IV numbers reversed test	AzBIO sentences in quiet		*(ns)*
(Moberly and Reed, 2019)	N=41<br>Mean age= 67.1 (range 50.0-83.0)<br>CI use = 7.3 years (range 1.5-34)	WAIS-III visual digit span forward and backward	Meaningful and anomalous sentences in quiet	Blockwise multiple linear regression analysis 2 blocks<br>% correct meaningful sentences as dependent measure<br>Block 1 BU: covariates + anomalous sentence scores<br>Block 2 TD: neurocognitive measures. df=32<br>(n)	*(ns)* Meaningful: *β=-0.010, p=0.910,* anomalous: *β=0.335, p=0.740*, df=32
		TOWRE	Meaningful and anomalous sentences in quiet		(+) Adding TOWRE words to predict anomalous sentences β=0.391, p=0.010 *(ns)* meaningful: *β=-0.81, p=0.414*, df=32
		Stroop task	Meaningful and anomalous sentences in quiet		(-) Adding Stroop to the model to predict meaningful SQ β=-0.259, p=0.008, *(ns)* anomalous:, *β=0.163, p=0.273*, df=32
		Ravens Progressive Matrices (RPM)	Meaningful and anomalous sentences in quiet		(+) Adding ravens to predict anomalous sentences β=0.421, p=0.08 *(ns)* meaningful: *β=-0.141, p=0.173* , df=32
(Mussoi and Brown, 2019)	N=20<br>Mean age= 51.3(range 50.0-83.0)<br>CI use = 4.0 years (1.2-9.2 range)	WAIS-III symbol search task	Quick SIN	Zero-order correlations (n)	*(ns)*
		WAIS-III coding task	Quick SIN		*(ns)*
		WAIS-III audiovisual digits span	Quick SIN		(+) r=0.573, p=0.018
(Dingemanse and Goedegebure, 2019)	N=50<br>Mean age= 63.0 (range 29.0-89.0)<br>CI use = at least 1 year	Reading span	CNC	Spearman correlation coefficients (n)	*(ns) rho=0.09, p=0.58*
			VU98 sentences in quiet and noise		(+) Words: r=0.37, p=0.011, Sentence: r=0.38, p=0.009
(Tamati et al., 2020)	N=21<br>(same participants as(Moberly et al., 2018a, 2018b, 2021))<br>Mean age= 62.1 (range 45.0-67.0)<br>CI use = 5.7 years (range 1.0-11.0)	WAIS-III visual digit span	PRESTO	Pearson correlations, discriminant function analysis (df=10) (n)	Matrix coefficient= 0.00, rank 10
		Computerized Stroop task	PRESTO		Control: Matrix coefficient= -0.08, rank 7<br>Interference: Matrix coefficient= 0.06, rank 8
		CVLT	PRESTO		List B: Matrix coefficient= 0.16, rank 5<br>Y/N discriminability: Matrix coefficient= 0.12, rank 6

					T1/T5: Matrix coefficient= -0.04, rank 9
		TOWRE	PRESTO		Words: Matrix coefficient= 0.25, rank 3<br>Nonwords: Matrix coefficient= 0.22, rank 4
		Ravens Progressive Matrices (RPM)	PRESTO		Matrix coefficient= 0.35, rank 2
(Kessler et al., 2020)	N=21<br>Divided in two groups based on performance Gottingen sentence test cut-off 7.6 dB SNR<br>Mean age= 62.2 (range 30.0-80.0)<br>CI use =115.2 months (range 13.0-492.0)	Size comparison span task (SicSpan)	Freiburg Monosyllabic word test (FMWT)<br>HSM sentence test in quiet and 10 dB noise<br>Göttingen sentence test with adaptive noise	Independent T-test (n)	*(ns)*
		Merfachwahl wortschatz inteligenz test (MWT-B)	Freiburg Monosyllabic word test (FMWT)<br>HSM sentence test in quiet and 10 dB noise<br>Göttingen sentence test with adaptive noise		*(ns)*
		CERAD-plus verbal test battery	Freiburg Monosyllabic word test (FMWT)<br>HSM sentence test in quiet and 10 dB noise<br>Göttingen sentence test with adaptive noise		*(ns)*
		Regensburger wortflussigkeits test (RWT)	Freiburg Monosyllabic word test (FMWT)<br>HSM sentence test in quiet and 10 dB noise<br>Göttingen sentence test with adaptive noise		*(ns)*
(Skidmore et al., 2020)	N=40<br>Mean age= 67.0 (range 48.0-83.0)<br>CI use = at least 1 year	Visual digit span	Harvard sentences<br>PRESTO<br>CUNY auditory & audiovisual<br>CID-words	Partial least squares regression (n)	*(ns)*
		Visual object span	Harvard sentences<br>PRESTO<br>CUNY auditory & audiovisual<br>CID-words		*(ns)*
		Visual symbol span	Harvard sentences<br>PRESTO<br>CUNY auditory & audiovisual<br>CID-words		*(ns)*
		Stroop task	Harvard sentences<br>Presto<br>CUNY auditory & audiovisual<br>CID-words		*(ns)*
		Ravens Progressive Matrices (RPM)	Harvard sentences<br>PRESTO<br>CUNY auditory & audiovisual<br>CID-words		*(ns)*
		TOWRE	Harvard sentences<br>PRESTO<br>CUNY auditory & audiovisual<br>CID-words		*(ns)*
		CVLT-II	Harvard sentences<br>PRESTO<br>CUNY auditory & audiovisual<br>CID-words		*(ns)*
		Wordfam	Harvard sentences<br>PRESTO		*(ns)*

			CUNY auditory & audiovisual<br>CID-words		
		WRAT wordreading	Harvard sentences<br>PRESTO<br>CUNY auditory & audiovisual<br>CID-words		*(ns)*
(Tinnemore et al., 2020)	N=10<br>Mean age= 57.2 (range 21.0-78.0)<br>CI use = at least 1 year	NIH toolbox flanker inhibitory control and attention test	IEEE audiovisual sentences in 6 conditions:<br>With distractors or secondary tasks x accented/unaccented	Generalised linear mixed-effects regression analysis (n)	(+) p=0.006 (for normal hearing, but no interaction, so same results for CI)
		NIH toolbox pattern comparison processing speed test	IEEE audiovisual sentences in 6 conditions:<br>With distractors or secondary tasks x accented/unaccented		*(ns)*
		NIH toolbox dimension change card sort	IEEE audiovisual sentences in 6 conditions:<br>With distractors or secondary tasks x accented/unaccented		(+) Higher than average scores associated with speech recognition p=0.006, estimate=0.35
(Wazen et al., 2020)<br><br>Cognitive measure preoperativley	N=40<br>Mean age= 78.2 (range 65.0-97.0)<br>CI use =12 months	Self-administered Gerocognitive Examination (SAGE)	CNC words	Linear correlation (n)	(+) $r^2(32)=0.1955$, p=0.0025<br>language p=0.01<br>visuospatial p=0.007<br>executive control p=0.03<br>memory p=0.02<br>reasoning p=0.02
			AzBio in quiet and mutlitalker babble noise		(+) quiet : $r^2(32)=0.1564$, p=0.0067<br>noise: $r^2(32)=0.1543$, p=0.007
(Zhan et al., 2020)<br><br>Cognitive measure preoperatively	N=19<br>Mean age= 67.8 (range 49.0-82.0)<br>CI use =6 months	WAIS visual digit span	AzBio in quiet and noise	Pearson correlations (n)	*(ns)* quiet: *r=0.309, p=0.198* noise*: r=0.44, p=0.057*
			CID words		*(ns) r=0.269, p=0.265*
			Harvard sentences		*(ns)* standard: *r=0.333, p=0.163,* anomalous *r=0.232, p=0.339*
			PRESTO		*(ns) r=0.418 p=0.075*
		WAIS visual object span	AzBio in quiet and noise		*(ns)* quiet: *r=0.355, p=0.136* noise: *r=0.426, p=0.069*
			CID words		*(ns) r=0.196, p=0.421*
			Harvard sentences		*(ns)* standard: *r=0.253, p=0.296,* anomalous *r=0.125, p=0.609*
			PRESTO		*(ns): r=0.241, p=0.321*
		WAIS visual symbol span	AzBio in quiet and noise		(+) quiet: r=0.504, p=0.028, noise: r=0.486, p=0.035
			CID words		(+) r=0.599 p=0.007
			Harvard sentences		(+)standard: r=0.541, p=0.017, *(ns)* anomalous *r=0.345, p=0.148*
			PRESTO		*(ns) r=0.443, p=0.057*
		Stroop task	AzBio in quiet and noise		(+) Incongruent: quiet: r=-0.484, p=0.042<br>*(ns)* incongruent: noise: *r=-0.412, p=0.09*
			CID words		*(ns) r=-0.455, p=0.058*
			Harvard sentences		*(ns)* incongruent: standard *r=-0.321, p=0.193,* anomalous: *r=-0.319, p=0.197,*

			PRESTO		*(ns)* incongruent: *r=-0.301, p=0.224*
		Ravens Progressive Matrices (RPM)	AzBio in quiet and noise		*(ns)* quiet: *r=0.253, p=0.295* noise: *r=0.167 p=0.493*
			CID words		*(ns) r=0.196, p=0.421*
			Harvard sentences		*(ns)* standard: *r=0.208, p=0.392* , anomalous: *r=0.212, p=0.383*
			PRESTO		*(ns) r=0.295, p=0.221,*
(Bosen et al., 2021)	N=20 Mean age= 59.8 (range 22.0-76.0) CI use =10.8 years (range 1.0-19.0)	Auditory digit span	PRESTO	Pearson correlation (n)	(+) r=0.51, p=0.03, after correcting for auditory resolution *(ns) r=0.39, p=0.08*
		WordFam	PRESTO		*(ns) r=0.21, p=0.39,* corrected for age *r=0.16, p=0.50*
(Moberly et al., 2021)	N=51 Divided in 3 groups based on SMRT performance high >2.1 RPO N=18, intermediate>1.3 N=17, low<1.3 N=16 (same participants as(Moberly et al., 2018a, 2018b; Tamati and Moberly, 2021)) Mean age= 66.8 (range 45.0-87.0) CI use =6.7 years (range 1.0-34.0)	Visual digit span	CID words	ANOVA & Spearmans rank-order correlations (n)	*(ns)* Low-smrt: *rho=-0.18, p=0.25,* intermediate-smrt: *rho=0.19, p=0.23,* high-smrt: *rho=-0.01, p=0.49*
			Harvard sentences		*(ns)* low-smrt: *rho=0.11, p=0.34,* intermediate-smrt: *rho=0.44, p=0.05,* high-smrt: *rho=-0.05, p=0.42*
			PRESTO		(+)Intermediate-smrt: rho=0.49, p=0.03, *(ns)* low-smrt: *rho=-0.07, p=0.40,* high-smrt: *rho=-0.03, p=0.46*
		Computerised Stroop task	CID words		(-) High-smrt: rho=-0.49, p=0.02, *(ns)* low-smrt: *rho=0.31, p=0.12,* intermediate-smrt: *rho=0.09, p=0.36*
			Harvard sentences		(-)High-smrt: rho=-0.047, p=0.03, *(ns)* low-smrt: *rho=0.27, p=0.16,* intermediate-smrt: *rho=-0.35, p=0.09,*
			PRESTO		*(ns)* low-smrt: *rho=0.40, p=0.06,* intermediate-smrt: *rho=-0.40, p=0.07,* high-smrt: *rho=-0.35, p=0.08*
		Ravens Progressive Matrices (RPM)	CID words		(+) Low-smrt group: rho=0.52, p=0.02 *(ns)* intermediate-smrt: *rho=0.33, p=0.10,* high-smrt: *rho=0.42, p=0.05*
			Harvard sentences		*(ns)* low-smrt: *rho=0.30, p=0.13,* intermediate-smrt: *rho=0.44, p=0.05,* high-smrt: *rho=0.22, p=0.19*
			PRESTO		(+)High-smrt group: r=0.52, p=0.01 *(ns)* low-smrt: *rho=0.26, p=0.17,* intermediate-smrt: *rho=0.35, p=0.09*
		TOWRE	CID words		(+) intermediate-smrt: rho=0.48, p=0.03, high-smrt: rho=0.42, p=0.05 *(ns)* low-smrt: *rho=-0.08, p=0.39,*
			Harvard sentences		*(ns)* low-smrt: *rho=-0.23, p=0.20,* intermediate-smrt: *rho=0.35, p=0.09,*

					High-smrt: rho=-0.23, p=0.20
			PRESTO		(ns) low-smrt: rho=-0.31, p=0.12, intermediate-smrt: rho=0.30, p=0.13, high-smrt: rho=0.37, p=0.07
(Tamati et al., 2021)	N=48<br>Mean age= 66.8 (range 45.0-83.0)<br>CI use =at least 1 year	TOWRE	Harvard sentences	Pearson correlations<br><br>(additionally WRAT word reading, WordFam, WAIS visual digit span, computerized Stroop task, Ravens Progressive Matrices (RPM) correlated with TOWRE) (n)	(+) Real words and Harvard standard r=0.36, p=0.015, real words and Harvard anomalous r=0.42, p=0.004, total and Harvard standard r=0.35, p=0.018, total and Harvard anomalous r=0.36, p=0.016,
			PRESTO		(+) Real words and PRESTO words r=0.40, p=0.006, total and PRESTO words r=0.47, p=0.014
(Tamati and Moberly, 2021)	N=15<br>Mean age= 66.9 (range 39.0-87.0)<br>CI use =7.2 years (range 1.0-14.0)	WAIS visual digit span	Hard and easy word recognition over time	Pearson correlations (n)	(ns) Q1 easy: r=-0.15, p=0.350 hard: r=0.19, p=0.246 Q4 easy: r=-0.14, p=0.371 hard r=0.17, p=0.269 TA easy: r=-0.04 p=0.444 hard r=0.04, p=0.448
		Computerised Stroop task	Hard and easy word recognition over time		(-) Later in time for hard words: r=-0.50, p=0.044 (ns) Q1: easy r=-0.22, p=0.317 hard r=-0.27, p=0.197 Q4: easy r=-0.05, p=0.430 TA: easy r=0.14 p=0.371 hard r=-0.58 p=0.072
		Ravens Progressive Matrices (RPM)	Hard and easy word recognition over time		(+) Later in time for hard words: r=0.68, p=0.009 (ns) Q1 easy: r=-0.09, p=0.372 hard: r=0.32, p=0.180 Q4 easy: r=0.14, p=0.371 hard: r=0.47, p=0.048 TA easy: r=0.26, p=0.371 hard: r=-0.42, p=0.116
(Völter et al., 2021)	Npoorer=15<br>Nbetter=19<br><br>Divided by High <70% and low>30% score on Freiburg Monosyllabic word test (FMWT)<br>Mean age low= 71.6, high=67.0 (range 46.0-85.0)<br>CI use low= 4.9, high=6.0 years (range 1.0-20.0)	ALAcog M3 attentional task	Freiburg Monosyllabic word test (FMWT)	Rank ANOVA & discriminant function analysis (n)	(+) Cohen's d=1.12, p=0.003 Discriminant r=0.50
		ALAcog recall and delayed recall	Freiburg Monosyllabic word test (FMWT)		(+) Delayed recall, Cohen's d = 0.88, p=0.04 Discriminant r=0.29 (ns) recall: Cohen's d=0.6, p=0.12
		Alacog 2-back test	Freiburg Monosyllabic word test (FMWT)		(ns) Cohen's d=0.5, p=0.22
		Alacog Operation Span task (OSPAN)	Freiburg Monosyllabic word test (FMWT)		(+) Cohen's d=1.01, p=0.0068
		ALAcog flanker task	Freiburg Monosyllabic word test (FMWT)		(+) Cohen's d=0.58, p=0.037, Discriminant r=0.21
		ALAcog Trail Making Test-A (TMT-A)	Freiburg Monosyllabic word test (FMWT)		(ns) Cohen's d=0.8, p=0.053
		ALAcog Trail Making Test-B (TMT-B)	Freiburg Monosyllabic word test (FMWT)		(+) Cohen's d=0.96, p=0.018
		Verbal fluency test	Freiburg Monosyllabic word test (FMWT)		(+) Cohen's d=0.80, p=0.025,
		Text Reception Threshold (TRT)	Freiburg Monosyllabic word test (FMWT)		(-) Periodic bars Cohen's d=-1.57, p=0.00002,

					Floating bars Cohen's d=-1.25, p=0.00021, Random dots Cohen's d=-0.94, p=0.0021
		Lexical decision test	Freiburg Monosyllabic word test (FMWT)		(-/+) Sensitivity Cohen's d=-1.27, p=0.0021 Discriminant r=0.54, Response time existing words Cohen's d=0.85, p=0.017,
		Lexical Model Oriented (LEMO) subtest of internal homophonic word reading	Freiburg Monosyllabic word test (FMWT)		(+)Cohen's d=-1.23, p=0.0039
		Rapid automatic namig (RAN)	Freiburg Monosyllabic word test (FMWT)		(-) objects Cohen's d=-1.28, p=0.0026, Colours: Cohen's d=-0.82, p=0.031Letters: Cohen's d=-1.25, p=0.0026 Numbers: Cohen's d=-1.34, p=0.0038, Discriminant: r=0.56
(Zucca et al., 2022)<br><br>Cognitive measure preoperatively	N=15 Mean age= 65.0 (range 50.0-80.0) CI use =12 months	Mini-Mental State Examination (MMSE)	Words in quiet	Regression analysis (n)	*(ns) p=0.545; r^2=0.061, β=0.247, p=0.280*
		Clock drawing test	Words in quiet		*(ns) p=0.117; r^2=0.177, β=0.421, p=0.058*
		Rey 15 words auditory learning test (RAVLT)	Words in quiet		*(ns)* immediate*: p=0.343; r^2=0.049, β= 0.222, p=0.346* differite*: p=0.455: r^2=0.110, β=0.331, p=0.154*
		Digit Span test	Words in quiet		*(ns)* forward: *p=0.199; r^2=0.003, β=0.051, p=0.826,* backward: *p=0.382; r^2=0.036, β=0.190, p=0.410*
		Corsi block tapping test	Words in quiet		*(ns)* backward: *p=0.220; r^2=0.103, β=0.284, p=0.156,* forward: *p=0.588;, r^2=0.081, β=0.321, p=0.212*
		Verbal fluency test (phonemic & semantic)	Words in quiet		*(ns)* Phonemic: *p=0.218; r^2=0.002, β=0.049, p=0.834* semantic*: p=0.052; r^2=0.165, , β=0.407, p=0.067*
		Trail Making Test-A & B (TMT-A & -B)	Words in quiet		(+) TMT-A r^2=0.236, β=-0.486 , p=0.035, *(ns) p=0.115 (ns)* TMT-B *p=0.087, r^2=0.086, β =-0.370, p=0.119*
(Ray et al., 2022)	N=32 Mean age= 68.5, standard deviation = 11.1 CI use = 6 months	CVLT-II	AzBio in quiet and +10dB babble noise	Partial least squares regression with VIP, (robust approach regarding sample size)	(+) Most important variables: short-delay cued recall, semantic clustering, subjective clustering, primacy recall and recall consistency. (VIP more than 1), Refittet model 35.8% explained, Each variable explained more than 50% of the variance
(Walia et al., 2022)	N=39 Mean age= 71.4 standard deviation = 16.4 CI use = 3 months	MoCA	Azbio sentence +10 dB in noise	Simple and multiple linear regression analyses, df=29 (y, but not sufficient)	In a model with a measure of neuronal health (Ecochg-TR) MoCA scores explained 64.5% of the variance β=2.06 p<0.05, or in

					interaction with Ecochg-TR β=0.12 p<0.05.
(Luo et al., 2022)	N=14<br>Mean age= 71.8 (range 63.0-82.0)<br>CI use =8.9 years (range 2.0 – 14)	Auditory digit span	Cued Modality working memory task	Pearson correlations (n)	*(ns)* WN: *r=-0.27, p=0.35* SN*: (ns) r=-0.21, p=0.48*
		Visual letter span	Cued Modality working memory task		*(ns)* WN: *r=-0.27, p=0.35,* SN: *r=-0.11, p=0.71*
		Reading span	Cued Modality working memory task		*(ns)* WN: *r=-0.05, p=0.86* SN: *r=-0.03, p=0.91*
		Cued Modality working memory task	Azbio sentences with babble noise		(-) Auditory cued working memory: r=-0.66, p=0.01<br>Auditory uncued working memory: r=-0.54, p=0.0045
			Words in noise		(-) Auditory cued working memory: r =-0.54, p=0.0047<br>Auditory uncued working memory: r=-0.60, p=0.02<br>→ after correcting for auditory resolution: r=-0.65, p=0.03

References supplementary material

Balota, D. A., Yap, M. J., Cortese, M. J., Hutchison, K. A., Kessler, B., Loftis, B., et al. (2007). The English Lexicon Project.

Bosen, A. K., Sevich, V. A., and Cannon, S. A. (2021). Forward digit span and word familiarity do not correlate with differences in speech recognition in individuals with cochlear implants after accounting for auditory resolution. *Journal of Speech, Language, and Hearing Research* 64, 3330–3342. doi: 10.1044/2021_JSLHR-20-00574.

Buckley, K. A., and Tobey, E. A. (2010). Cross-Modal Plasticity and Speech Perception in Pre-and Postlingually Deaf Cochlear Implant Users. *Ear Hear* 32, 2–15.

Chen, L. C., Puschmann, S., and Debener, S. (2017). Increased cross-modal functional connectivity in cochlear implant users. *Sci Rep* 7. doi: 10.1038/s41598-017-10792-2.

Chen, L. C., Sandmann, P., Thorne, J. D., Bleichner, M. G., and Debener, S. (2016). Cross-modal functional reorganization of visual and auditory cortex in adult cochlear implant users identified with fNIRS. *Neural Plast* 2016. doi: 10.1155/2016/4382656.

Cognition assessment using the NIH Toolbox (2013). Department of Medical Social Sciences.

Collison, E. A., Munson, B., and Carney, A. E. ; (2004). Relations among linguistic and cognitive skills and spoken word recognition in adults with cochlear implants. *J Speech Lang Hear Res* 47, 469–508.

de Groot, A. M. B., Borgwaldt, S., Bos, M., and van den Eijnden, E. (2002). Lexical decision and word naming in bilinguals: Language effects and task effects. *J Mem Lang* 47, 91–124. doi: 10.1006/jmla.2001.2840.

Dingemanse, G. J., and Goedegebure, A. (2019). The Important Role of Contextual Information in Speech Perception in Cochlear Implant Users and Its Consequences in Speech Tests. *Trends Hear* 23. doi: 10.1177/2331216519838672.

Feld, J. E., and Sommers, M. S. (2009). Lipreading, Processing Speed, and Working Memory in Younger and Older Adults. *Journal of Speech, Language, and Hearing Research* 52, 1555–1565. doi: 10.1044/1092-4388(2009/08-0137).

Finke, M., Büchner, A., Ruigendijk, E., Meyer, M., and Sandmann, P. (2016). On the relationship between auditory cognition and speech intelligibility in cochlear implant users: An ERP study. *Neuropsychologia* 87, 169–181. doi: 10.1016/j.neuropsychologia.2016.05.019.

Folstein, M. F., Folstein, S. E., and Mchugh, P. R. (1975). “MINI-MENTAL STATE” A PRACTICAL METHOD FOR GRADING THE COGNITIVE STATE OF PATIENTS FOR THE CLINICIAN*. Pergamon Press.

Gilbert, J. L., Tamati, T. N., and Pisoni, D. B. (2013). Development, reliability, and validity of PRESTO: A new high-variability sentence recognition test. *J Am Acad Audiol* 24, 26–36. doi: 10.3766/jaaa.24.1.4.

Han, J. H., Lee, H. J., Kang, H., Oh, S. H., and Lee, D. S. (2019). Brain plasticity can predict the cochlear implant outcome in adult-onset deafness. *Front Hum Neurosci* 13. doi: 10.3389/fnhum.2019.00038.

Haumann, S., Hohmann, V., Meis, M., Herzke, T., Lenarz, T., and Büchner, A. (2012). Indication Criteria for Cochlear Implants and Hearing Aids: Impact of Audiological and Non-Audiological Findings. *Audiol Res* 2, e12. doi: 10.4081/audiores.2012.e12.

Hay-McCutcheon, M. J., Pisoni, D. B., and Kirk, K. I. (2005). Audiovisual speech perception in elderly cochlear implant recipients. *Laryngoscope* 115, 1887–1894. doi: 10.1097/01.mlg.0000173197.94769.ba.

Hillyer, J., Elkins, E., Hazlewood, C., Watson, S. D., Arenberg, J. G., and Parbery-Clark, A. (2019). Assessing cognitive abilities in high-performing cochlear implant users. *Front Neurosci* 13. doi: 10.3389/fnins.2018.01056.

Holden, L. K., Finley, C. C., Firszt, J. B., Holden, T. A., Brenner, C., Potts, L. G., et al. (2013). Factors affecting open-set word recognition in adults with cochlear implants. *Ear Hear* 34, 342–360. doi: 10.1097/AUD.0b013e3182741aa7.

Hua, H., Johansson, B., Magnusson, L., Lyxell, B., and Ellis, R. J. (2017). Speech recognition and cognitive skills in bimodal cochlear implant users. *Journal of Speech, Language, and Hearing Research* 60, 2752–2763. doi: 10.1044/2017_JSLHR-H-16-0276.

Kaandorp, M. W., Smits, C., Merkus, P., Festen, J. M., and Goverts, S. T. (2017). Lexical-Access Ability and Cognitive Predictors of Speech Recognition in Noise in Adult Cochlear Implant Users. *Trends Hear* 21. doi: 10.1177/2331216517743887.

Kaandorp, M. W., Smits, C., Merkus, P., Goverts, S. T., and Festen, J. M. (2015). Assessing speech recognition abilities with digits in noise in cochlear implant and hearing aid users. *Int J Audiol* 54, 48–57. doi: 10.3109/14992027.2014.945623.

Kessler, M., Schierholz, I., Mamach, M., Wilke, F., Hahne, A., Büchner, A., et al. (2020). Combined Brain-Perfusion SPECT and EEG Measurements Suggest Distinct Strategies for Speech Comprehension in CI Users With Higher and Lower Performance. *Front Neurosci* 14. doi: 10.3389/fnins.2020.00787.

Killion, M. C., Niquette, P. A., Gudmundsen, G. I., Revit, L. J., and Banerjee, S. (2004). Development of a quick speech-in-noise test for measuring signal-to-noise ratio loss in normal-hearing and hearing-impaired listeners. *J Acoust Soc Am* 116, 2395–2405. doi: 10.1121/1.1784440.

Kim, M. B., Shim, H. Y., Jin, S. H., Kang, S., Woo, J., Han, J. C., et al. (2016). Cross-Modal and intra-modal characteristics of visual function and speech perception performance in postlingually deafened, cochlear implant users. *PLoS One* 11. doi: 10.1371/journal.pone.0148466.

Knopke, S., Bauknecht, H. C., Gräbel, S., Häußler, S. M., Szczepek, A. J., and Olze, H. (2021). White matter lesions as possible predictors of audiological performance in adults after cochlear implantation. *Brain Sci* 11. doi: 10.3390/brainsci11050600.

Kollmeier, B., and Wesselkamp, M. (1997). Development and evaluation of a German sentence test for objective and subjective speech intelligibility assessment. *J Acoust Soc Am* 102, 2412–2421. doi: 10.1121/1.419624.

Layer, N., Weglage, A., Müller, V., Meister, H., Lang-Roth, R., Walger, M., et al. (2022). The timecourse of multisensory speech processing in unilaterally stimulated cochlear implant users revealed by ERPs. *Neuroimage Clin* 34. doi: 10.1016/j.nicl.2022.102982.

Lazard, D. S., and Giraud, A. L. (2017). Faster phonological processing and right occipito-temporal coupling in deaf adults signal poor cochlear implant outcome. *Nat Commun* 8. doi: 10.1038/ncomms14872.

Lazard, D. S., Giraud, A. L., Truy, E., and Lee, H. J. (2011). Evolution of non-speech sound memory in postlingual deafness: Implications for cochlear implant rehabilitation. *Neuropsychologia* 49, 2475–2482. doi: 10.1016/j.neuropsychologia.2011.04.025.

Lazard, D. S., Lee, H. J., Gaebler, M., Kell, C. A., Truy, E., and Giraud, A. L. (2010). Phonological processing in post-lingual deafness and cochlear implant outcome. *Neuroimage* 49, 3443–3451. doi: 10.1016/j.neuroimage.2009.11.013.

Luo, X., Azuma, T., Kolberg, C., and Pulling, K. R. (2022). The effects of stimulus modality, task complexity, and cuing on working memory and the relationship with speech recognition in older cochlear implant users. *J Commun Disord* 95. doi: 10.1016/j.jcomdis.2021.106170.

Mattingly, J. K., Castellanos, I., and Moberly, A. C. (2018). Nonverbal reasoning as a contributor to sentence recognition outcomes in adults with cochlear implants. *Otology and Neurotology* 39, e956–e963. doi: 10.1097/MAO.0000000000001998.

Moberly, A. C., Castellanos, I., Vasil, K. J., Adunka, O. F., and Pisoni, D. B. (2018a). "product" Versus "process" Measures in Assessing Speech Recognition Outcomes in Adults with Cochlear Implants. *Otology and Neurotology* 39, e195–e202. doi: 10.1097/MAO.0000000000001694.

Moberly, A. C., Harris, M. S., Boyce, L., and Nittrouer, S. (2017a). Speech recognition in adults with cochlear implants: The effects of working memory, phonological sensitivity, and aging. *Journal of Speech, Language, and Hearing Research* 60, 1046–1061. doi: 10.1044/2016_JSLHR-H-16-0119.

Moberly, A. C., Houston, D. M., and Castellanos, I. (2016). Non-auditory neurocognitive skills contribute to speech recognition in adults with cochlear implants. *Laryngoscope Investig Otolaryngol* 1, 154–162. doi: 10.1002/lio2.38.

Moberly, A. C., Houston, D. M., Harris, M. S., Adunka, O. F., and Castellanos, I. (2017b). Verbal working memory and inhibition-concentration in adults with cochlear implants. *Laryngoscope Investig Otolaryngol* 2, 254–261. doi: 10.1002/lio2.90.

Moberly, A. C., Lewis, J. H., Vasil, K. J., Ray, C., and Tamati, T. N. (2021). Bottom-Up Signal Quality Impacts the Role of Top-Down Cognitive-Linguistic Processing During Speech Recognition by Adults with Cochlear Implants. *Otol Neurotol* 42, S33–S41. doi: 10.1097/MAO.0000000000003377.

Moberly, A. C., Pisoni, D. B., and Harris, M. S. (2017c). Visual working memory span in adults with cochlear implants: Some preliminary findings. *World J Otorhinolaryngol Head Neck Surg* 3, 224–230. doi: 10.1016/j.wjorl.2017.12.003.

Moberly, A. C., and Reed, J. (2019). Making sense of sentences: Top-down processing of speech by adult cochlear implant users. *Journal of Speech, Language, and Hearing Research* 62, 2895–2905. doi: 10.1044/2019_JSLHR-H-18-0472.

Moberly, A. C., Vasil, K. J., Wucinich, T. L., Safdar, N., Boyce, L., Roup, C., et al. (2018b). How does aging affect recognition of spectrally degraded speech? *Laryngoscope* 128, S1–S16. doi: 10.1002/lary.27457.

Mortensen, M. V., Mirz, F., and Gjedde, A. (2006). Restored speech comprehension linked to activity in left inferior prefrontal and right temporal cortices in postlingual deafness. *Neuroimage* 31, 842–852. doi: 10.1016/j.neuroimage.2005.12.020.

Mussoi, B. S. S., and Brown, C. J. (2019). Age-Related Changes in Temporal Resolution Revisited: Electrophysiological and Behavioral Findings from Cochlear Implant Users. *Ear Hear* 40, 1328–1344. doi: 10.1097/AUD.0000000000000732.

O'Neill, E. R., Kreft, H. A., and Oxenham, A. J. (2019). Cognitive factors contribute to speech perception in cochlear-implant users and age-matched normal-hearing listeners under vocoded conditions. *J Acoust Soc Am* 146, 195–210. doi: 10.1121/1.5116009.

Pisoni, D. B., Broadstock, A., Wucinich, T., Safdar, N., Miller, K., Hernandez, L. R., et al. (2018). Verbal learning and memory after cochlear implantation in postlingually deaf adults: Some new findings with the CVLT-II. *Ear Hear* 39, 720–745. doi: 10.1097/AUD.0000000000000530.

Raven, J. (2000). The Raven's Progressive Matrices: Change and Stability over Culture and Time. *Cogn Psychol* 41, 1–48. doi: 10.1006/cogp.1999.0735.

Ray, C., Pisoni, D. B., Lu, E., Kronenberger, W. G., and Moberly, A. C. (2022). Preoperative Visual Measures of Verbal Learning and Memory and their Relations to Speech Recognition after Cochlear Implantation. *Ear Hear* 43, 993–1002. doi: 10.1097/AUD.0000000000001155.

Sandmann, P., Dillier, N., Eichele, T., Meyer, M., Kegel, A., Pascual-Marqui, R. D., et al. (2012). Visual activation of auditory cortex reflects maladaptive plasticity in cochlear implant users. *Brain* 135, 555–568. doi: 10.1093/brain/awr329.

Scharre, D. W., Chang, S.-I., Murden, R. A., Lamb, J., Beversdorf, D. Q., Kataki, M., et al. (2010). Self-administered Gerocognitive Examination (SAGE) A Brief Cognitive Assessment Instrument for Mild Cognitive Impairment (MCI) and Early Dementia. Available at: www.sagetest.

Shulman, K. I. [(1999). CLOCK!DRAWING] IS IT THE IDEAL COGNITIVE SCREENING TEST<.

Skidmore, J. A., Vasil, K. J., He, S., and Moberly, A. C. (2020). Explaining Speech Recognition and Quality of Life Outcomes in Adult Cochlear Implant Users: Complementary Contributions of Demographic, Sensory, and Cognitive Factors. *Otology and Neurotology* 41, e795–e803. doi: 10.1097/MAO.0000000000002682.

Song, J. J., Lee, H. J., Kang, H., Lee, D. S., Chang, S. O., and Oh, S. H. (2015). Effects of congruent and incongruent visual cues on speech perception and brain activity in cochlear implant users. *Brain Struct Funct* 220, 1109–1125. doi: 10.1007/s00429-013-0704-6.

Sörqvist, P., Ljungberg, J. K., and Ljung, R. (2010). A sub-process view of working memory capacity: Evidence from effects of speech on prose memory. *Memory* 18, 310–326. doi: 10.1080/09658211003601530.

Spahr, A. J., Dorman, M. F., Litvak, L. M., Wie, S. van, Gifford, R. H., Loizou, P. C., et al. (2012). Development and Validation of the AzBio Sentence Lists.

Strelnikov, K., Rouger, J., Demonet, J. F., Lagleyre, S., Fraysse, B., Deguine, O., et al. (2013). Visual activity predicts auditory recovery from deafness after adult cochlear implantation. *Brain* 136, 3682–3695. doi: 10.1093/brain/awt274.

Suh, M. W., Park, K. T. ae, Lee, H. J., Lee, J. H. o., Chang, S. O., and Oh, S. H. a. (2015). Factors Contributing to Speech Performance in Elderly Cochlear Implanted Patients: An FDG-PET Study: A Preliminary Study. *J Int Adv Otol* 11, 98–103. doi: 10.5152/iao.2015.424.

Sun, Z., Seo, J. W., Park, H. J., Lee, J. Y., Kwak, M. Y., Kim, Y., et al. (2021). Cortical reorganization following auditory deprivation predicts cochlear implant performance in postlingually deaf adults. *Hum Brain Mapp* 42, 233–244. doi: 10.1002/hbm.25219.

Tamati, T. N., and Moberly, A. C. (2021). Talker Adaptation and Lexical Difficulty Impact Word Recognition in Adults with Cochlear Implants. *Audiology and Neurotology,* 1–10. doi: 10.1159/000518643.

Tamati, T. N., Ray, C., Vasil, K. J., Pisoni, D. B., and Moberly, A. C. (2020). High- and Low-Performing Adult Cochlear Implant Users on High-Variability Sentence Recognition: Differences in Auditory Spectral Resolution and Neurocognitive Functioning. *J Am Acad Audiol* 31, 324–335. doi: 10.3766/jaaa.18106.

Tamati, T. N., Vasil, K. J., Kronenberger, W. G., Pisoni, D. B., Moberly, A. C., and Ray, C. (2021). Word and Nonword Reading Efficiency in Postlingually Deafened Adult Cochlear Implant Users. *Otol Neurotol* 42, e272–e278. doi: 10.1097/MAO.0000000000002925.

Tinnemore, A. R., Gordon-Salant, S., and Goupell, M. J. (2020). Audiovisual Speech Recognition With a Cochlear Implant and Increased Perceptual and Cognitive Demands. *Trends Hear* 24. doi: 10.1177/2331216520960601.

Versfeld, N. J., Daalder, L., Festen, J. M., and Houtgast, T. (2000). Method for the selection of sentence materials for efficient measurement of the speech reception threshold. *J Acoust Soc Am* 107, 1671–1684. doi: 10.1121/1.428451.

Völter, C., Götze, L., Falkenstein, M., Dazert, S., and Thomas, J. P. (2017). Application of a computer-based neurocognitive assessment battery in the elderly with and without hearing loss. *Clin Interv Aging* 12, 1681–1690. doi: 10.2147/CIA.S142541.

Völter, C., Oberländer, K., Carroll, R., Dazert, S., Lentz, B., Martin, R., et al. (2021). Nonauditory Functions in Low-performing Adult Cochlear Implant Users. *Otol Neurotol* 42, e543–e551. doi: 10.1097/MAO.0000000000003033.

Walia, A., Shew, M. A., Kallogjeri, D., Wick, C. C., Durakovic, N., Lefler, S. M., et al. (2022). Electrocochleography and cognition are important predictors of speech perception outcomes in noise for cochlear implant recipients. *Sci Rep* 12. doi: 10.1038/s41598-022-07175-7.

Wazen, J. J., Kellermeyer, B., Lange, L., Rende, S., Ortega, C., and Rosenberg, S. (2020). Predicting Speech Outcomes after Cochlear Implantation in Older Adults Using the Self-administered Gerocognitive Examination Test. *Otology and Neurotology* 41, E28–E35. doi: 10.1097/MAO.0000000000002425.

Zekveld, A. A., George, E. L. J., Kramer, S. E., Goverts, S. T., and Houtgast, T. (2007). The Development of the Text Reception Threshold Test: A Visual Analogue of the Speech Reception Threshold Test. *Journal of Speech, Language, and Hearing Research* 50, 576–584. doi: 10.1044/1092-4388(2007/040).

Zelazo, P. D., Anderson, J. E., Richler, J., Wallner-Allen, K., Beaumont, J. L., Conway, K. P., et al. (2014). NIH toolbox cognition battery (CB): Validation of executive function measures in adults. *Journal of the International Neuropsychological Society* 20, 620–629. doi: 10.1017/S1355617714000472.

Zhan, K. Y., Lewis, J. H., Vasil, K. J., Tamati, T. N., Harris, M. S., Pisoni, D. B., et al. (2020). Cognitive Functions in Adults Receiving Cochlear Implants: Predictors of Speech Recognition and Changes after Implantation. *Otology and Neurotology* 41, e322–e329. doi: 10.1097/MAO.0000000000002544.

Zhou, X., Seghouane, A. K., Shah, A., Innes-Brown, H., Cross, W., Litovsky, R., et al. (2018). Cortical Speech Processing in Postlingually Deaf Adult Cochlear Implant Users, as Revealed by Functional Near-Infrared Spectroscopy. *Trends Hear* 22. doi: 10.1177/2331216518786850.

Zucca, M., Albera, A., Albera, R., Montuschi, C., Gatta, B. della, Canale, A., et al. (2022). Cochlear Implant Results in Older Adults with Post-Lingual Deafness: The Role of "Top-Down" Neurocognitive Mechanisms. *Int J Environ Res Public Health* 19. doi: 10.3390/ijerph19031343.
